# Curcumin Delivery Systems: From Importance and Microencapsulation Techniques to Molecular Docking and Dynamics for Rational Formulation Design

**DOI:** 10.1007/s41061-026-00553-z

**Published:** 2026-05-01

**Authors:** Romică Crețu, Simona Butan, Veronica Filimon, Alexandra Virginia Bounegru, Aurel Tăbăcaru

**Affiliations:** 1 Department of Chemistry, Physics and Environment, Faculty of Sciences and Environment, “Dunărea de Jos” University of Galati, 111 Domneasca Street, 800201 Galati, Romania; 2 Cross-Border Faculty, “Dunarea de Jos” University, 111 Domneasca, 800201 Galati, Romania

**Keywords:** Curcumin, Microencapsulation, Biomacromolecular carriers, *In silico* modeling, Structure–function relationships, Bioavailability enhancement

## Abstract

Curcumin, a polyphenolic compound from *Curcuma longa*, has many biological effects, including antioxidant, anti-inflammatory, anticancer, and neuroprotective properties. However, its use in food, pharmaceutical, and biomedical systems is limited owing to poor water solubility, chemical instability, fast metabolism, and very low oral bioavailability. To address these issues, various formulation strategies have been created. Microencapsulation is one of the most effective methods for improving the stability, bioaccessibility, and controlled release of curcumin. At the same time, computational tools such as molecular docking and molecular dynamics simulations have become more important for understanding curcumin–carrier interactions and predicting formulation stability at the molecular level. Although both experimental encapsulation techniques and in silico modeling are well-established, research in these areas often occurs separately, leading to fragmented understanding of curcumin delivery systems. This review offers a detailed analysis of curcumin research by connecting its physicochemical properties and degradation pathways with microencapsulation strategies and computational modeling. Key encapsulation techniques such as spray drying, ionotropic gelation, complex coacervation, and nanostructured delivery systems are examined in terms of their mechanisms, benefits, drawbacks, and uses. Additionally, recent progress in molecular docking and molecular dynamics simulations is discussed to emphasize their growing role in helping choose carriers and design formulations. By linking formulation science with predictions at the molecular level, this review presents a framework to promote the development of effective, stable, and bioavailable curcumin-based delivery systems for food, pharmaceutical, and biomedical purposes.

## Introduction

Curcumin, the main bioactive polyphenolic compound isolated from the rhizome of *Curcuma longa*, has been extensively investigated owing to its wide range of biological activities, including antioxidant, anti-inflammatory, anticancer, neuroprotective, and antimicrobial effects [1–7]. These properties are associated with curcumin’s ability to regulate multiple molecular targets and signaling pathways, positioning it as a promising candidate for applications in food, pharmaceutical, and biomedical fields. However, despite strong experimental evidence supporting its bioactivity, the practical implementation of curcumin into effective products is highly limited.

The major limitation affecting the use of curcumin is its unfavorable physicochemical and pharmacokinetic profile. Curcumin exhibits extremely low aqueous solubility, pronounced chemical instability, and rapid metabolic transformation, resulting in very low oral bioavailability [1, 2]. In particular, curcumin undergoes rapid degradation under neutral and alkaline conditions, is sensitive to light and oxidative environments, and is extensively metabolized after absorption, leading to systemic concentrations insufficient to exert therapeutic effects [2, 3]. These intrinsic limitations represent a significant hurdle in the development of curcumin-based formulations and require advanced technological intervention.

Among the strategies proposed to overcome these challenges, microencapsulation has emerged as one of the most effective and versatile approaches [3–7]. By incorporating curcumin into protective micro- or nanostructured matrices, encapsulation technologies can significantly improve the physicochemical stability, enhance the apparent solubility, protect the compound against environmental degradation, and enable controlled release during gastrointestinal transit. Over the past decade, a wide range of encapsulation techniques have been investigated, including spray drying, ionotropic gelation, complex coacervation, and nanostructured delivery systems, using diverse polymeric, protein-based, and lipid-based carriers. The growing number of published studies in this area reflects the recognition of microencapsulation as an important enabling technology for improving the bioaccessibility and bioavailability of curcumin [6, 7].

**Fig. 1:**
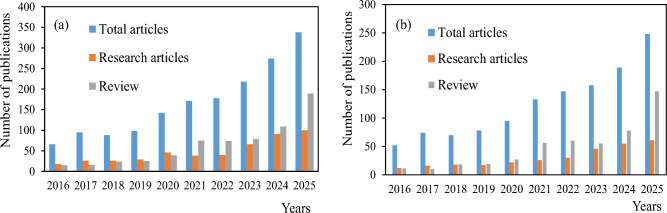
Recent research trends regarding the chronological profile of the impact of curcumin microencapsulation on its stability (**a**) and bioavailability (**b**)

Over the past decade, there has been significant increase in research related to curcumin encapsulation, reflecting increasing efforts to overcome its intrinsic limitations in terms of stability and bioavailability. This trend is depicted in Fig. 1, which presents the chronological evolution of publications addressing curcumin microencapsulation with respect to stability and bioavailability, based on database searches performed over the last 10 years. The pronounced increase in both research and review articles highlights not only the continued scientific interest in curcumin delivery systems but also the growing diversity of encapsulation strategies proposed to address these challenges. At the same time, the rapid expansion of the literature underlines the need for integrated and critical analyses able to link formulation technologies with mechanistic understanding and predictive tools.

In parallel with experimental formulation research, computational approaches such as molecular docking and molecular dynamics simulations have gained increasing relevance for the study of curcumin at the molecular level. These in silico tools provide valuable insights into curcumin-carrier interactions, conformational stability, and environmental response, and have been widely applied to investigate curcumin binding to proteins, polymers, and lipid assemblies. However, these computational studies are often conducted independently from experimental formulation development, limiting their direct impact on the rational design of encapsulation systems. Despite the considerable progress achieved in the development of curcumin delivery systems, formulation strategies remain largely empirical, frequently relying on iterative adjustments of carrier composition, processing conditions, and stabilization approaches [8]. This trial-and-error paradigm often leads to inconsistent outcomes in terms of encapsulation efficiency, physicochemical stability, and bioavailability, particularly when curcumin is exposed to complex environmental conditions during processing, storage, or gastrointestinal transit. In this context, computational modeling has emerged as a valuable complementary approach for elucidating the molecular mechanisms governing curcumin encapsulation and release. Molecular docking studies can provide insight into the affinity and binding modes between curcumin and biomacromolecular carriers, while molecular dynamics simulations enable the evaluation of conformational stability, structural organization, and environmental responsiveness of encapsulated systems [9]. Such molecular-level information can support the rational selection of carrier materials, predict formulation performance under variable physicochemical conditions, and reduce the need for extensive empirical optimization. Therefore, the integration of in silico modeling with experimental encapsulation technologies represents an important step toward mechanism-driven formulation design. By correlating molecular interaction patterns with macroscopic stability and release behavior, this combined strategy offers new opportunities for developing curcumin delivery systems with improved functional performance, reproducibility, and application relevance in the food, pharmaceutical, and biomedical fields.

**Fig. 2:**
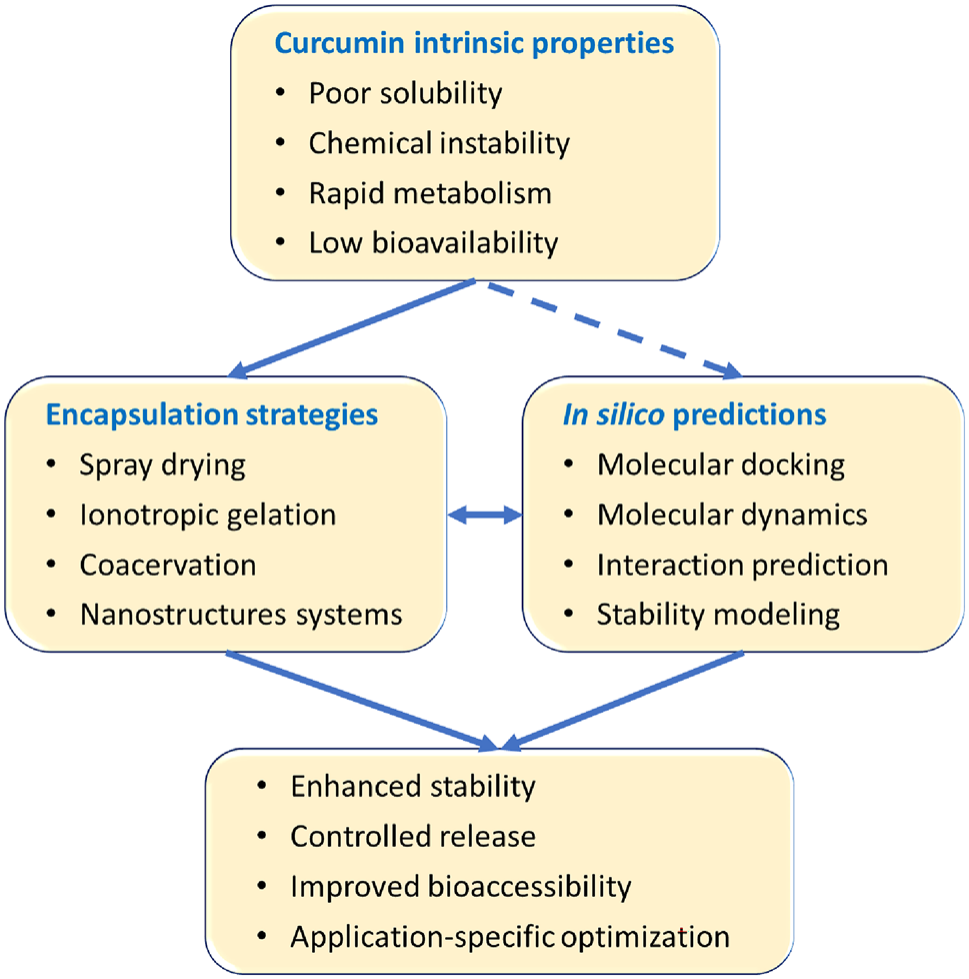
Conceptual framework illustrating the iterative integration of curcumin properties, encapsulation strategies, molecular-level prediction, and the emerging improved properties (the dashed arrow indicates partial correlation with in silico modeling, particularly for chemical instability)

Despite the substantial body of literature individually addressing the bioactivity of, encapsulation technologies for, and computational modeling of curcumin, the available research is fragmented. A comprehensive framework that systematically links the intrinsic physicochemical constraints of curcumin to microencapsulation strategies and molecular-level predictive tools is still lacking. The present review addresses this gap by critically integrating experimental encapsulation approaches with molecular docking and molecular dynamics simulations. By correlating formulation mechanisms, stability and bioavailability results, and in silico predictions, this work aims to support the rational, mechanism-driven design of efficient and stable curcumin delivery systems for food, pharmaceutical, and biomedical applications (Fig. 2).

## Curcumin: Structure, Biological Relevance, and Intrinsic Limitations

### Chemical Structure and Physicochemical Properties

Curcuminoids are polyphenolic compounds found in the rhizome of *Curcuma longa* L. (turmeric), a plant with about 100 species that vary in chemical composition [10]. The curcuminoid family includes three main members: curcumin (about 77% of total curcuminoids), desmethoxycurcumin (DMC, about 17%), and bisdemethoxycurcumin (BDMC, about 3%). These compounds give turmeric its characteristic yellow color and are responsible for its broad spectrum of biological activity [11]. Curcumin accounts for 60–70% of the total curcuminoids in fresh turmeric rhizome and, together with desmethoxycurcumin, bisdemethoxycurcumin, and cyclocurcumin, form the famous “Indian saffron” that is responsible for the intense color [10].

**Fig. 3:**
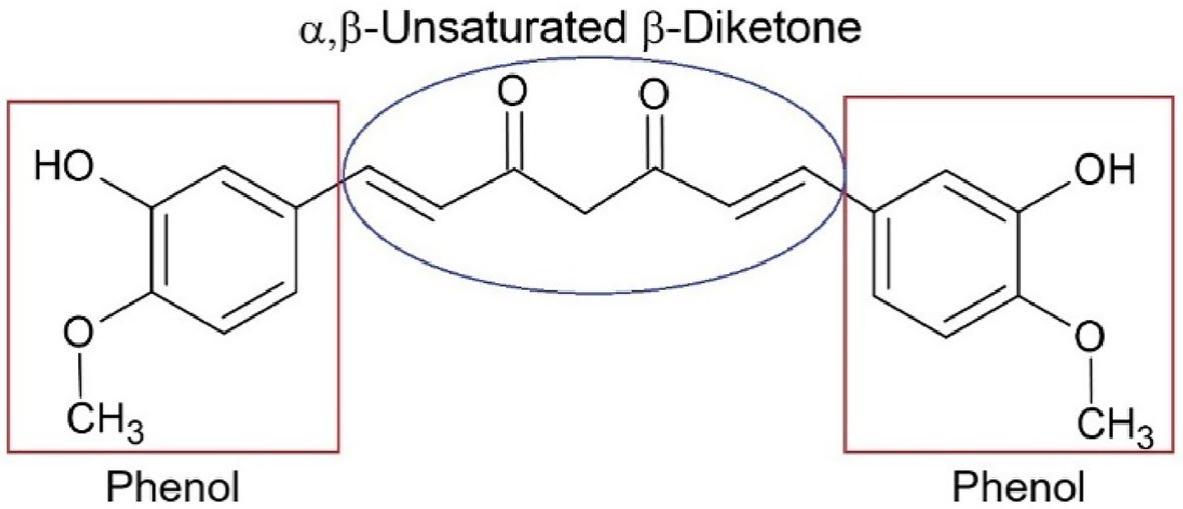
Chemical structure of curcumin

Chemically, curcumin is a diarylheptanoid, formally named 1,7-bis-(4-hydroxy-3-methoxyphenyl)-1,6-heptadiene-3,5-dione (Fig. 3). Its molecular structure is symmetrical, and it is also called diferuloylmethane, because it has two ferulic acid residues joined by a methylene bridge. This seven-carbon diketone skeleton is what gives the molecule its unique chemical behavior, sensitivity to the environment, and pharmacological effects.

Curcumin is an amphiphilic molecule, containing both hydrophilic and hydrophobic moieties. This duality allows it to be partially soluble in both water and organic solvents, although its central polarity controls how it interacts with different environments. The most dynamic structural feature of curcumin is keto–enol tautomerism (equilibrium between diketone and ketoenol forms). In most conditions, the ketoenol form predominates. However, the environment in which curcumin is found influences its conformation. In nonpolar solvents, it prefers a closed conformation (*cis*-enol) stabilized by intramolecular hydrogen bonds (IMHB). In polar solvents, it adopts an open conformation, forming intermolecular hydrogen bonds with the solvent [12, 13]. This tautomeric behavior contributes to curcumin’s ability to interact with a wide range of biomolecules, including proteins, lipids, and polysaccharides, but also underlies its physicochemical instability and limited aqueous solubility. This feature has major impacts on formulation development, as it limits its dissolution, absorption, and homogeneous dispersion in biological environments, thereby necessitating the use of advanced delivery systems.

**Fig. 4:**
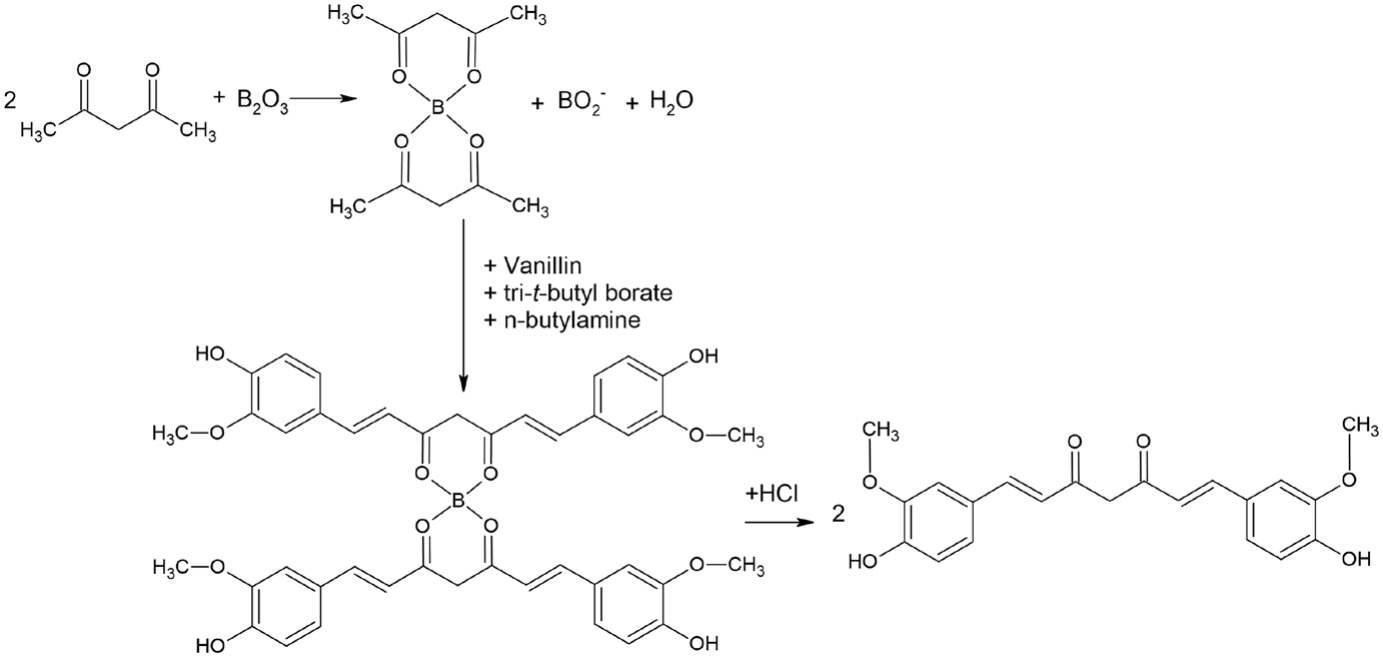
Pabon synthesis of curcumin [12]

Regarding its production, although curcumin was initially synthesized in the laboratory by the Lampe method, the most widespread and used method remains the Pabon method (Fig. 4) [12], which involves an aldol condensation between vanillin and acetylacetone.

Research also focuses on the synthesis of derivatives (compounds that maintain the seven-atom diketone bridge, such as glycosylated or silylated ones) and analogs (compounds with structural variations, classified into natural and synthetic), with the aim of improving the poor absorption of the molecule. Structural modifications are varied, from the creation of heterocycles at the central bridge (such as pyrazoles or pyrimidines, with anticancer activity), to the incorporation of ferrocenyl, or the use of cyclodextrins to enhance its solubility and bioavailability in vivo.

### Biological Activity and Molecular Mechanisms

Curcumin shows remarkable therapeutic potential owing to its pleiotropic (pluripotent) nature, acting on over 30 distinct proteins, including nuclear factor kappa-light-chain-enhancer of activated B cells (NF-κB), p53, and thioredoxin reductase, and forming complexes with divalent metal ions. Being an α,β-unsaturated β-diketone, the biological activity of curcumin is defined by three main functional groups: the hydroxyl group (OH-phenolic), which gives the molecule antioxidant properties, being the most vulnerable point to free radical attack and acting as a strong hydrogen donor, the ketone groups, and the C = C double bonds, which ensure its anti-inflammatory and anticancer action [12].

The anticancer effect of curcumin is manifested by its ability to inhibit the growth of cancer cells, acting by suppressing the formation of new blood vessels (angiogenesis) and inducing programmed cell death (apoptosis), according to in vitro and in vivo studies on various types of cancer (prostate, breast, and colon) [1]. Its mechanisms are specific, depending on the type of neoplasm. For example, in prostate cancer, curcumin regulates proliferation by attacking epidermal growth factor receptors and suppresses cell motility and bone metastasis [14]. In breast cancer, it induces apoptosis by suppressing the expression of NF-kB, cyclin D, and matrix metalloproteinase-1 (MMP-1) [15]. In colon cancer, it reduces microRNA (miR)-21 activity by inhibiting cells in the G_2_/M phase, thus affecting progression and metastasis [16].

On the other hand, curcumin also exerts a significant immunomodulatory effect by regulating the balance of pro- and anti-inflammatory cytokines and modulating the activity of immune cells (such as lymphocytes, macrophages, and natural killer (NK) cells), often acting by inhibiting the transcription factor NF-κB and other signaling pathways associated with inflammation [17–20]. Research confirms its ability to modulate both innate and adaptive immune responses [21, 22]. Curcumin also exhibits analgesic effects that extend beyond simply reducing inflammation [23–25]. Clinical evidence shows that curcumin supplementation significantly reduces chronic pain, with benefits gradually appearing over 8–12 weeks [26]. Very recent research actually confirmed the efficacy of curcumin in pain management, when formulated for improved bioavailability [27].

The antioxidant effect of curcumin is demonstrated by a mechanism of action that includes free radical scavenging by hydrogen donation, singlet oxygen inhibition, and chelation of iron ions (Fe^3+^ and Fe^2+^). Specifically, the β-diketone structure allows curcumin to chelate transition metal ions, preventing metal-catalyzed oxidative damage, and enzymatically, it stimulates endogenous defenses by increasing the expression and activity of superoxide dismutase, catalase, and glutathione peroxidase [28]. Comparative studies show that this bioactive compound is extremely effective, with a much lower concentration (20 mM) required to inhibit lipid oxidation compared with recognized antioxidants such as butylated hydroxyanisole, Trolox, or tocopherol [29].

Curcumin has also shown neuroprotective properties, relevant for the prevention of neurodegenerative diseases and the improvement of cognition [30, 31]. In the treatment of Alzheimer’s disease, curcumin acts by inhibiting the formation of β-amyloid plaques and by chelating copper(I), owing to its structure with phenolic nuclei and enol groups that disrupt protein aggregation/clumping [32]. It also increases the expression of brain-derived neurotrophic factor (BDNF), promoting neurogenesis and synaptic plasticity [33].

Of particular importance is the antiviral activity of this compound, by inhibiting the replication of RNA and DNA viruses through mechanisms such as blocking attachment to cells or interfering with viral proteins. Recent studies highlight its efficacy against viruses such as human immunodeficiency virus (HIV), Zika, influenza A, and severe acute respiratory syndrome coronavirus 1 (SARS-CoV-1), although low bioavailability limits clinical application, being improved by derivatives and nanoparticles [34].

However, it is important to emphasize that many of the reported biological effects of curcumin are observed at relatively high concentrations under controlled experimental conditions. In vivo, such concentrations are challenging to achieve and maintain owing to rapid degradation, limited absorption, and extensive metabolism. Many studies conducted in the last decade have shown that, under the action of factors such as pH, temperature, light, and the presence of metal ions, curcumin can rapidly degrade following an extremely complex process. Such substantial chemical changes can have a significant negative impact on its biological activity [35]. According to studies conducted by Schneider et al. [36], the degradation of curcumin in vitro occurs rapidly through an auto-oxidative transformation. Various reactive chemical species are thus obtained. Moreover, results regarding the behavior of curcumin showed that its decomposition is dependent on pH and temperature, occurring faster in neutral–basic conditions. On the other hand, curcumin can easily decompose at high temperatures, although recent studies [37] have shown that, when microencapsulated, curcumin is effectively protected from decomposition under heat treatment according to differential scanning calorimetry (DSC) analysis [38]. Furthermore, Huang et al. [39] showed in their studies that the degradation rate of curcumin is reduced by microencapsulation, according to the technique used, associated with reasonable thermal stability (between 40–100 °C). However, when curcumin was microencapsulated in porous starch using a spray dryer and subsequently subjected to heating, increasing the temperature to 170 °C caused significant changes, with the encapsulated particles suffering serious collapse. Other authors have shown in their studies that the level of curcumin decreases with the time of heat exposure of curcumin extract microcapsules, this aspect being dependent on the type of matrix used [40]. As a result, the intrinsic bioactivity of curcumin does not directly translate into consistent therapeutic efficacy, thus highlighting a significant gap between experimental potential and practical application.

### Chemical Instability, Bioavailability Constraints, and Pharmacokinetic Limitations

Despite the remarkable pharmacological potential, clinical use remains limited by certain challenges, such as poor water solubility, chemical instability, and extremely low oral bioavailability [41, 42]. Curcumin exhibits pronounced instability under physiological conditions, especially at the alkaline pH that is characteristic of the intestinal lumen. Degradation studies reveal pH-dependent stability, with half-lives ranging from 100–200 min at acidic pH, but decreasing dramatically to 1–9 min at physiological intestinal pH (7.2–8.0) [41, 43].

The degradation mechanism involves nucleophilic attack on the β-diketone group, leading to the formation of degradation products (ferulic acid and vanillin) that have significantly reduced biological activities compared with curcumin. In addition to this pH sensitivity, curcumin is highly sensitive to photodegradation, oxidative stress, and thermal degradation, factors that are particularly relevant during processing, storage, and gastrointestinal transit [44–46]. These instability pathways are not only detrimental to the efficacy of curcumin, but also complicate its formulation and handling. Exposure to light, oxygen, or elevated temperature during manufacturing can cause significant loss of activity, underscoring the need for protective strategies capable of preserving the integrity of curcumin throughout the product lifecycle.

In addition to chemical instability, curcumin exhibits extremely low oral bioavailability, which further limits its clinical and nutritional applications. Absorbed curcumin undergoes extensive phase II conjugation reactions, mainly glucuronidation and sulfation. The resulting curcumin glucuronides and sulfates exhibit dramatically lower biological activity than the parent curcumin. The mechanisms underlying this poor bioavailability are diverse [41, 42]. First, the limited solubility of curcumin in water prevents dissolution in gastrointestinal fluids [47]. Second, curcumin exhibits limited permeability through intestinal epithelial membranes [48]. Recent studies by Liu et al. suggest that novel formulation approaches can simultaneously improve the solubility and permeability of curcumin [49]. Third, curcumin serves as a substrate for efflux transporters (P-glycoprotein and breast cancer resistance protein (BCRP)), which actively pump absorbed curcumin back into the intestinal lumen [42]. Fourth, extensive first-pass metabolism dramatically reduces the bioavailability, with a large portion undergoing conjugation soon after absorption [41]. As a result, conventional curcumin formulations often fail to achieve therapeutic levels in target tissues.

These pharmacokinetic limitations explain why curcumin, despite its potent molecular activity, exhibits inconsistent efficacy in clinical studies. Overcoming these barriers requires formulation strategies that not only enhance its solubility and stability, but also control its absorption, metabolism, and release profiles.

### Implications for Encapsulation Strategies and In Silico Approaches

The intrinsic physicochemical properties, instability mechanisms, and pharmacokinetic barriers related to curcumin collectively define the requirements for effective delivery systems. Encapsulation strategies should provide a hydrophobic microenvironment to solubilize curcumin, protect reactive functional groups from degradation, and enable controlled release under physiological conditions. Also, the carrier materials must be biocompatible, scalable, and suitable for the intended application, whether food, pharmaceutical, or biomedical.

At the molecular level, the interaction between curcumin and encapsulating materials plays a decisive role in determining the encapsulation efficiency, stability, and release behavior. In this context, molecular docking and molecular dynamics simulations represent powerful tools to investigate curcumin–carrier interactions, predicting the binding affinity, conformational stability, and environmental response. These in silico approaches can complement experimental studies by providing mechanistic insights and guiding rational materials selection.

By integrating an understanding of the structure and limitations of curcumin with encapsulation technologies and molecular-level modeling, it becomes possible to move from empirical formulation development toward a predictive, mechanism-driven design strategy. The following sections examine how different microencapsulation techniques address these challenges and how computational tools can further enhance formulation optimization.

## Encapsulation Techniques to Improve the Stability and Bioaccessibility of Curcumin

**Fig. 5:**
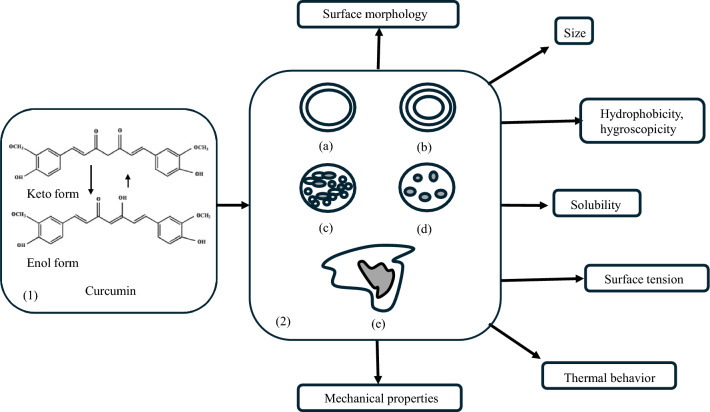
Keto–enol tautomerism of curcumin (1), different types of microcapsules (2): monolayer (**a**); multilayer (**b**); matrix (**c**); multicore (**d**); irregular (**e**) and fundamental elements of related research

The efficiency of curcumin encapsulation techniques has a direct impact on the physicochemical, biological, and functional stability of the resulting formulations. In previous reviews on curcumin, various encapsulation techniques were described in detail [50–55]. In addition, aspects related to the ability of these techniques to result in optimized stability and bioavailability have been analyzed [56]. Thus, Efstathia et al. reported, on the one hand, the degree of photochemical stability of microencapsulated curcumin and, on the other hand, the stability of curcumin microcapsules during storage at constant temperature and humidity when protected from light [57]. According to Choudhury et al., any bioactive compound can be protected from such harmful conditions using a method that has been widely researched during the last decade, viz. encapsulation [58]. Microcapsules produced using various techniques have different sizes that depend on the techniques used, an aspect that has a direct impact on their stability. According to the same authors, capsules with a simple spherical shape surrounded by one or more layers of material, and even capsules with irregular shapes, can be obtained in this way. In addition, advanced research on some crucial properties of these microcapsules is necessary to characterize and determine their long-term stability (Fig. 5).

In light of these research directions, recent scientific advances have increasingly explored the impact of some parameter changes, such as pH, on the final properties of curcumin microcapsules. This aspect, associated with the correct choice of encapsulating agents, may lead to better understanding of how the stability of curcumin microcapsules can be improved. Thus, by identifying the optimal pH values, a potential strategy to increase the efficiency of curcumin microencapsulation can be developed. Also, the yields of such curcumin microcapsules are dependent on pH changes. According to studies by Kumavat et al., the stability of curcumin is better at acidic pH but decreases as the pH increases [59]. On the other hand, the use of composite material associations in the microencapsulation process, such as whey protein isolate (WPI)–β-cyclodextrin (βCD), leads to curcumin microcapsules with superior stability that present better physicochemical characteristics than single-coated materials [60]. This has a direct impact on the morphology of these microcapsules, whose surface characteristics depend on the formulation conditions. In this context, Zhu et al. showed that the development of nanoemulsions with curcumin encapsulation using quaternized chitosan leads to a promising delivery system, characterized by superior stability in terms of gastric digestion [56]. Such a nanoemulsion system demonstrates high storage stability, while presenting high antimicrobial and antioxidant properties, makes it suitable for applications in functional food and beverage systems.

Furthermore, recent studies show that a key factor regarding the encapsulation performance of curcumin lies in the correct choice of the pH of the solution in which the hydrophobic molecules are dissolved [61]. Indeed, this is the case for compounds with a polyphenolic structure solubilized in alkaline solutions, and thus, the structural changes achieved as a result of different pH values can significantly accelerate the solubilization of curcumin in formulations such as emulsions. By dissolving curcumin crystals in an alkaline solution, followed by rapid acidification, smaller curcumin particles result, a phenomenon directly correlated with the curcumin concentration. By using appropriate research methods, such as bright-field and fluorescence microscopy, it has been shown that, during this process, a transition from crystallized structures to smaller structures occurs, with the hydrophobic curcumin molecules migrating into the lipid phase of the emulsions.

The positive impact of curcumin microencapsulation is also reflected in the sensory stability of the obtained formulation. In this context, another possibility for monitoring the stability of a colloidal system containing curcumin is to associate it with the color stability of curcumin microcapsules. Such an approach consists in using a technique based on the CIELAB color space as defined by the Commission Internationale de l’Éclairage (CIE, International Commission on Illumination). Regarding the analysis of the color stability of microencapsulated curcumin, only a few studies have been reported in the literature on the importance of correlating it with the results obtained by using various encapsulation methods. Recent scientific research [62] shows that, by encapsulating substances with antioxidant, anti-inflammatory, and anticancer properties such as curcumin [63, 64] in niosomes, improved antioxidant capacity can be obtained, while at the same time colored niosomes were also obtained. Such colloidal systems are characterized by sufficiently good stability in terms of the target applications. These studies show that the particle size of curcuminoids decreased with addition of cholesterol, which was associated with higher stability. Further studies also showed that addition of cholesterol during the formulation process was sufficient to maintain the stability of such curcumin niosomes. Furthermore, this stability is supported by the extremely narrow range of color parameters. However, the color stability of curcumin depends on the microencapsulation techniques applied to the curcuminoids.

Comparative analysis across encapsulation strategies indicates that no single approach provides universal stabilization under all conditions. Instead, stabilization results depend on the match between the properties of curcumin, the carrier characteristics, and the intended application. Systems optimized for storage stability may differ from systems designed to protect curcumin during gastrointestinal transit or for controlled release at target sites. As a result, the rational selection of encapsulation strategies requires comprehensive understanding of both degradation mechanisms and carrier–curcumin interactions. In this context, insights from molecular docking and molecular dynamics simulations provide valuable support for the interpretation and prediction of stability results. By elucidating the strength and persistence of curcumin–carrier interactions, in silico approaches can help explain the observed differences in stability between encapsulation systems and guide the design of carriers that maximize the protective effects. This integration of experimental and computational methods reinforces the need for a mechanism-driven approach to curcumin stabilization.

Microencapsulation has a profound and measurable impact on the stability of curcumin, transforming an intrinsically unstable compound into a viable functional ingredient for food, pharmaceutical, and biomedical applications. The effectiveness of stabilization is strongly dependent on the system design, material selection, and encapsulation architecture, which emphasizes the importance of tailoring delivery systems to specific environmental challenges and application requirements. When combined with molecular-level insights provided by in silico modeling, microencapsulation strategies can be rationally optimized to maximize the safety and functional performance of curcumin.

### Encapsulation by Spray Drying

**Fig. 6:**
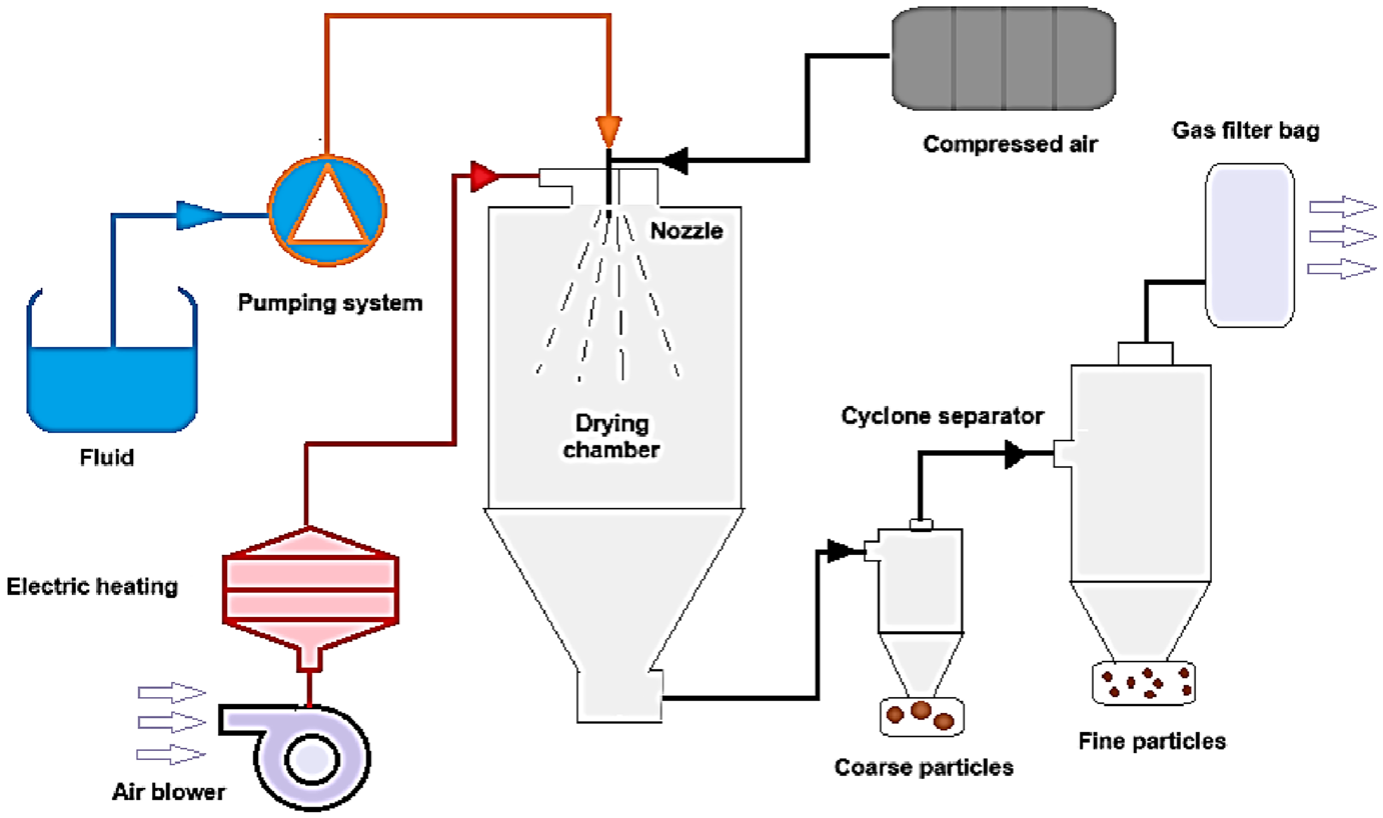
Schematic diagram of the spray-drying process

Spray drying is one of the most widely applied technologies for curcumin encapsulation owing to its industrial scalability, cost-effectiveness, and ability to generate stable powdered formulations. In recent years, research has increasingly focused on optimizing formulation variables, such as the wall material composition, emulsion stability, and processing temperature, to minimize the degradation of curcumin and enhance the encapsulation efficiency [64, 65]. This method produces microcapsules whose shape and morphology are influenced by the wall material (polysaccharides, lipid proteins, or combinations thereof in different proportions), the dispersion concentration, and the operating conditions of the drying device [66, 67]. The general workflow of the spray-drying encapsulation process is illustrated schematically in Fig. 6, while the following discussion focuses on the formulation-dependent performance, optimization strategies, and technological limitations specifically relevant to curcumin delivery systems.

For example, the use of hydrolyzed collagen as a wall material has demonstrated a remarkable ability to enhance curcumin bioavailability, while simultaneously maintaining high antioxidant and cyclooxygenase-2 (COX-2) inhibitory activity, highlighting the clear advantages of protein-based matrices over systems relying exclusively on carbohydrates [68]. These findings suggest that specific protein–curcumin interactions contribute not only to the protection of the bioactive compound during processing but also to its efficient in vivo absorption, thereby reinforcing the role of proteins as superior functional carriers.

A comparable approach, based on a combination of whey proteins and carbohydrates (maltodextrin and arabic gum), exhibited favorable behavior under gastrointestinal conditions, characterized by increased resistance to the gastric environment and controlled release at the intestinal level [69]. Unlike exclusively protein-based systems, these hybrid formulations provide an efficient balance between structural stability and biological functionality; however, their performance is strongly dependent on the formulation and processing conditions. Conversely, the use of arabic gum as the sole wall material allowed for a simplification of the technological process, but required relatively higher concentrations to achieve satisfactory encapsulation efficiency [70], underscoring the limitations of individually applied polysaccharides in curcumin protection.

The expansion of the range of carbohydrates employed, through the incorporation of pectin, starch, or d-mannitol, led to systems with improved stability and controlled release over extended periods [71]. Notably, starch–gelatin-based microcapsules exhibited superior performance compared with maltodextrin–gelatin systems, in terms of both their long-term stability and the curcumin retention under wide temperature variations [72]. This behavior was attributed to the amphiphilic nature of the modified starch, which promotes stable emulsification and the formation of more compact and less porous structures compared with the predominantly hydrophilic maltodextrin.

The role of proteins as advanced encapsulating materials was further confirmed by direct comparisons between whey protein isolates and soy protein isolates, with the former demonstrating significantly higher affinity for curcumin and superior encapsulation efficiency [73, 74]. These differences highlight that the selection of wall materials should not be based solely on economic or technological considerations but must also account for molecular compatibility and the ability to effectively stabilize the bioactive compound.

Despite its advantages, conventional spray drying presents notable technological limitations, such as the risk of nozzle clogging and the thermal degradation of curcumin. To address these drawbacks, alternative strategies have been proposed, including the use of Pickering nanoemulsions stabilized by protein nanoparticles, which resulted in microparticles with improved morphology and enhanced stability [75]. Nevertheless, the increased complexity of these formulations may limit their applicability at an industrial scale.

In this context, electric-field-based techniques, such as electrospraying, have attracted considerable interest owing to their ability to reduce thermal stress on curcumin. This method enables the production of smaller and more homogeneous particles, with high encapsulation efficiency and superior preservation of antioxidant activity compared with conventional spray drying [76–79]. However, the process stability remains sensitive to variations in operational parameters, which may affect the reproducibility and morphological control of the particles [77]. Through careful optimization of processing conditions, results comparable to or even superior to those obtained using conventional spray drying have been achieved, including enhanced bioaccessibility and stability within the digestive tract [80].

Nano-spray drying represents a significant evolution of the conventional technology, offering more precise control over the particle size, distribution, and morphology, as well as improved yields [81, 82]. Compared with conventional spray drying, this method allows processing at lower temperatures, thereby reducing curcumin degradation and improving the stability of the final product. Studies employing nano-spray drying have demonstrated efficient curcumin encapsulation and controlled release, although parameter optimization remains a critical and labor-intensive step [83].

Furthermore, the applicability of this technology has been extended, and it is considered suitable for nonfood domains, such as the coating of dental implants with curcumin nanoparticles, where reduced processing temperatures contributed to preserving the integrity of the bioactive compounds and improving the surface functional properties [84, 85].

A comparative analysis of curcumin encapsulation techniques based on electrospraying and spray drying (nano/electrostatic) reveals the importance of optimizing the specific parameters of each method and the encapsulation materials. For a more detailed understanding of the impact of these variables, Table 1 presents the key parameters and the results obtained for curcumin microcapsules, highlighting the efficiency and final properties of each product. The reported experimental data reveal that classical spray drying remains a preferred method at industrial level, owing to its scalability and low cost, but it imposes certain quality compromises, mainly due to the need to operate at high input temperatures (150–190 °C). This thermal stress poses a major risk for the degradation of curcumin and reduces its antioxidant activity. On the other hand, more advanced techniques, such as electrospraying and nano-spray drying, offer greatly superior performance by operating at much lower temperatures (even below 90 °C), ensuring maximum thermal protection and high encapsulation efficiency (over 80%). These methods also produce small, spherical nanoparticles with homogeneous dimensions (below 500 nm), improving the bioaccessibility and mechanical stability of the final product. However, the disadvantage of newer approaches is related to their industrial viability, as these methods require laborious optimization and may suffer from scalability and operational stability limitations, such as flux instability at high voltages in electrospraying, which makes them less useful for mass production at the present time.

**Table 1:** Comparison of curcumin encapsulation parameters for spray drying

Spray-drying technology	Spray-drying conditions	Wall material	Core material	Zeta potential (mV)	Yield (%)	Loading capacity/loading efficiency (%)	Encapsulation efficiency (%)	Particle diameter/size	Ref.
Spray drying	Inlet temp. 160 °C; flow rate 7 mL/min; airflow 473 L/h; Brix 36.7	Hydrolyzed collagen (30% w/v)	Nanoemulsion of curcumin (20% Tween 80, 0.0052 M MgCl_2_)	–	45	–	58	215 nm	[68]
Spray drying (co-current, nozzle atomizer)	Inlet temp. 180 °C; feed rate 5 mL/min; outlet temp. 80–85 °C	Whey protein blend (WPC-80) + maltodextrin (MD) + gum Arabic (GA) + skim milk	Curcumin dissolved in buffalo butter core: wall material solids ratio of 1:2 and protein:carbohydrates ratio of 1:2	9.27	–	–	97.59	579.55 nm	[69]
Spray drying	Feed flow rate 6 mL min^−1^; drying air flow rate 420 L h^−1^, inlet/outlet air temperatures 170/80 °C; nozzle diameter 1.5 mm	Modified starch 30% + gelatin 1%	Turmeric oleoresin (15% of the weight of the wall material)	–	40	–	72.3	< 2 μm to > 20 μm	[72]
Microfluidic-jet spray drying	Inlet air temperature 220 °C; outlet air temperature 90 °C; feed rate pressure 5–8 psi; nozzle orifice diameter 100 μm	Whey protein isolate (WPI) or soy protein isolate (SPI) solutions	Curcumin dissolved in anhydrous ethanol (30%)	–	–	9.6 3.6	97.6 77.1	39.6 μm 71.1 μm	[74]
Electrospraying	Maximum voltage 30 kV; fixed shell pump rate 0.2 mL/h; cylindrical collector rotation at 5 RPM	Milk protein isolate	Curcumin dissolved in ethanol	–	–	–	80.7	232 nm	[78]
Spray drying	Inlet temperature 120 °C; feed flow rate 4 mL/min; spray gas flow rate 670 L/h; aspiration rate 75%	2% cetyltrimethylammonium bromide-modified cellulose nanocrystals (CNC-CTAB)	Curcumin dissolved in ethanol at a 1:3 ratio	−17.73	81.97	28.63	82.61	7.33 µm	[79]
Spray drying	Inlet temperature 150 °C; air flow rate 120 L/min	Chitosan and Tween 20	Turmeric	–	–	–	Almost 100%	285 ± 30 nm	[83]
Nano-spray drying	Inlet temperature 75 °C; gas flow rate 100 L/min; spray rate 50%; feed rate 26.39 mL/h	–	Curcumin dissolved in an acetone:ethanol mixture	–	–	–	–	∼ 500 nm	[84]
Spray drying	Inlet temp. 150 °C; flow rate 15 mL/min; air blower 35 Hz; nozzle 0.7 mm	Chitosan and mannitol (1:3 w/w ratio)	Turmeric extract in 96% ethanol	+30.5 ± 0.2	56.40%	–	85.25	18.62 ± 0.76 µm	[86]
Vacuum spray drying	Inlet temp. 90 °C; pressure 0.01 mPa	Jelly fig pectin (0.75% w/w)	Turmeric	–	70.02 ± 1.96%	5.45 ± 0.14	91.56 ± 0.80	1.68 ± 0.41 to 2.98 ± 0.68 µm	[87]
Nano-spray drying	Inlet temp. 90 °C; feeding rate 25 mL/h; nitrogen flow 90 L/min; mesh spray pore size 7.0 mm	SD/P4 (lecithin:cholesterol:SA (m/m/m) 1:1:1, Polaxomer 188—0 mg, β-cyclodextrin 1 g) SD/P6 (lecithin:cholesterol:SA (m/m/m) 1:1:1, Polaxomer 188—6 mg, β-cyclodextrin 1 g)	Turmeric	+31.35 ± 4.31 to 34.95 ± 3.18	70.23 ± 2.45 to 76.60 ± 0.56	1.68 ± 0.06 to 1.78 ± 0.20	92.65 ± 2.90 to 92.40 ± 3.54	126.40 ± 3.25 to 150.65 ± 4.31 nm	[88]
Spray drying	Inlet air temperature 190 °C; feed pump rate 10 mL/min; drying air flow rate 473 L/h; aspiration ratio 24 m^3^/h; nozzle diameter 0.7 mm	Protein isolates, gum Arabic, and maltodextrin	Turmeric oil extract	–	87.38 ± 0.04	–	90.72	–	[89]
Spray drying	Inlet temp. 120° C; feed flow 0.23 L/h; atomizer nozzle diameter 0.7 mm; air flow rate 45 L/min; atomized air pressure 5.3 m^3^/min; outlet temp. 90 °C	Eudragit^®^ L100 (poly(methacrylic acid-co-methyl methacrylate) 1:1). Lactose (used as a drying aid for the spray-drying process)	Curcumin, medium-chain triglyceride, sorbitan monostearate (Span 60^®^)	-15 ± 0.42	87	–	98.9 ± 0.01	136 ± 0.96 nm	[90]
Spray drying	Inlet drying air temp.185 °C; feed rate 6 mL/min	Resistant starch, maltodextrin, and whey protein isolate	Curcumin at 5% wt. basis of wall material was dissolved in ghee taken at 10% wt. base	–	–	–	82.42	5.6 μm	[91]
Spray drying	Inlet temp. 150 ± 1 °C; outlet temp. 80 ± 2 °C; air flow rate 4 mL/min;air pressure 5 bar	Maltodextrin, gum Arabic, whey protein isolate, and their composites with β-cyclodextrin	Turmeric	–	–	–	–	2 μm	[92]
Spray drying	Air pressure 5–6 bar; aspiration rate 35 m^3^/h (90%); solution feed flow 4 mL/min; inlet air temp. 130 °C; outlet air temp. 63–71 °C	Alginic acid sodium and ethyl cellulose + polyethylene glycol (EC + PEG) blend	Retinoic acid, curcumin, and resveratrol	- 47.1 ± 0.6	27	6.7 ± 0.2	92 ± 4 for curcumin	–	[93]
Spray drying	Emulsion was spray dried at 165 °C	Gum Arabic	Curcumin and soy lecithin	–	–	–	65 ± 1	4.3 ± 2.0 µm	[94]

This comparative analysis of current spray-drying strategies suggests that a hybrid approach may represent an effective solution, notably through the application of vacuum spray drying combined with amphiphilic wall materials (e.g., modified starch/whey protein) and advanced preconcentration techniques such as Pickering nanoemulsions, enabling low-temperature processing while maintaining industrial feasibility. The trade-off between product quality and industrial feasibility therefore represents a key consideration when selecting spray-drying strategies for curcumin delivery systems.

On the other hand, according to the studies reported by Cano-Higuita et al., during storage of curcumin capsules, the color change was significantly greater (*P* < 0.05) in the spray-dried product than in the lyophilized product [95]. During storage of microcapsules obtained by spray drying, the degradation of curcumin was initially associated with the loss of pigment present on the surface of the particles, but after 1 month, the curcumin content remained almost constant, as a result of the inhibition of degradation by the encapsulation matrix. Also, in the case of microcapsules obtained by freeze drying, the degradation kinetics of curcumin was determined.

The reviewed studies show that the choice of formulation ingredients is the factor determining the stability of microencapsulated curcumin. In this context, for example, in the case of the spray-drying techniques, significant efficiency in preventing the change of trichromatic parameters in the case of microcapsules was obtained when a ternary mixture of gum Arabic, maltodextrin, and modified starch was used. In the case of other techniques, such as the freeze-drying technique, the use of pure gum Arabic led to the smallest color changes. The use of the spray-drying technique led to the highest luminosity (L*) value, indicating that the samples containing microencapsulated curcumin were significantly lighter than the microparticles obtained by the freeze-drying technique. The chroma (C*), one of the essential parameters in color monitoring, showed a small decrease only in the samples with gum Arabic that were prepared by the spray-drying technique. Thus, it should be considered that the type of technique used for the microencapsulation of curcumin is extremely important in terms of obtaining a stable color and, therefore, a more stable system. All these aspects are supported by recent studies showing that the color profile reflects very well the brightness and saturation levels of curcumin microcapsule particles [60]. Moreover, according to these authors, high values of the yellowness indicate that the microcapsules have a significant yellow color, which is associated with higher availability of curcumin in the case of microcapsules. However, this depends on the type of coating material used in the microencapsulation technique. Such results support the fact that microcapsules resulting from complexation with various materials show excellent stability, associated with the stability of their yellow color to sunlight, pH variations, temperature changes, and long-term storage, compared with the situation when natural dyes without complexation are used.

Spray drying is therefore a versatile and reliable method for curcumin encapsulation that can yield stable, free-flowing powders with customized release characteristics. This method can significantly enhance the stability and bioaccessibility whenof curcumin combined with suitable carrier materials and optimized processing conditions, However, its limitations underline the need for complementary or hybrid approaches, as well as predictive tools, to further improve formulation design. In this context, insights from molecular-level studies may assist in selecting compatible wall materials and improving the encapsulation performance, which would lessen the need for empirical optimization.

In comparison with polymer-network-based methods, such as ionotropic gelation or coacervation, spray drying provides more limited control over the microcapsule architecture and release profiles. Moreover, in situations where thermal stress and particle porosity become limiting factors, ionotropic gelation may represent a milder alternative. Nevertheless, the advantages of spray drying in terms of reproducibility, yield, and scalability continue to position it as a reference technology for industrial applications.

### Encapsulation by Ionotropic Gelation

Ionotropic gelation has been widely explored as a mild encapsulation strategy for curcumin, because it enables the formation of micro- and nanoscale delivery systems under aqueous and relatively low-energy processing conditions [96, 97]. Recent studies have demonstrated that the encapsulation performance is strongly influenced by the polymer composition, crosslinking density, and drying methodology, which together determine the particle stability, encapsulation efficiency, and release kinetics.

Of particular interest is chitosan, a cationic biopolymer that, at pH below 6, effectively interacts with multivalent anions (such as tripolyphosphate, alginate, or hyaluronate), leading to the formation of micro- and nanometric particles [98]. The properties of these structures depend on the gelation conditions, such as the ratio of the polymer to anionic agent or the homogenization method, influencing the size, rigidity, and stability of the particles [99, 100]. Owing to these properties, chitosan is frequently used for the encapsulation of bioactive molecules, such as curcumin. Using ionotropic gelation, curcumin can be incorporated into chitosan particles or chitosan–alginate hybrid systems, which leads to increased stability and bioavailability and expanded application possibilities in the pharmaceutical and food fields.

For example, the study by Wiratantri et al. [101] demonstrated the effectiveness of ionotropic gelation in the formulation of chitosan and alginate-based microcapsules by optimizing the matrix composition using a simplex lattice experimental design. This approach allowed the production of stable particles with nanometric dimensions and high encapsulation efficiency, while maintaining the antioxidant activity of curcumin. The results suggest that electrostatic interactions between polymers contribute significantly to the stabilization of the bioactive compound, improving both the dispersibility and the protection against degradation.

**Fig. 7:**
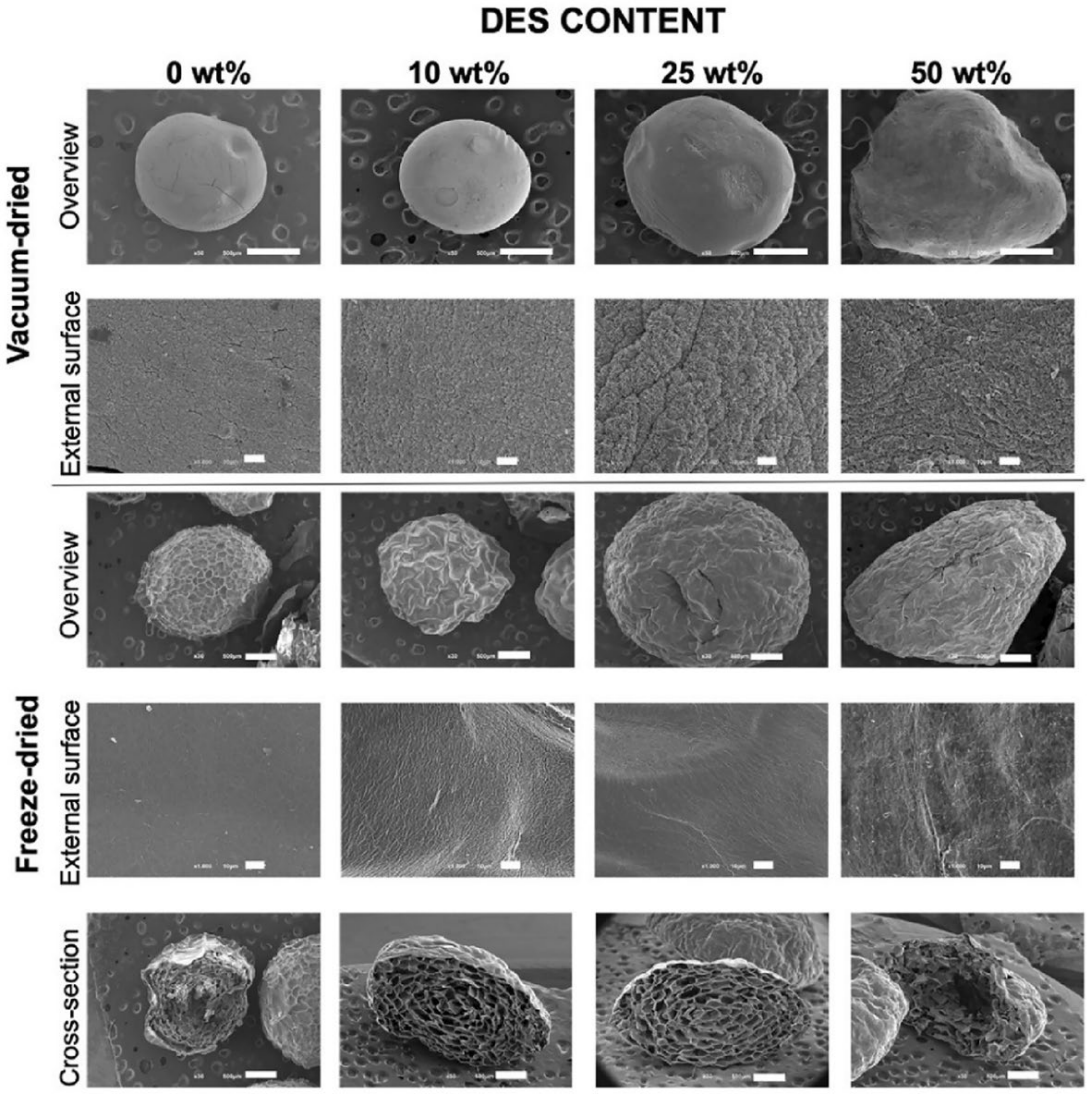
Scanning electron microscopy (SEM) images of vacuum-dried and freeze-dried hydrogel beads with varying DES–curcumin content [102]

A complementary approach was proposed by Silva et al. [102], who highlighted the advantages of using deep eutectic solvents (DES) to increase the solubility of curcumin and facilitate its incorporation into hydrogel microcapsules obtained from chitosan and alginate. This strategy allowed the avoidance of organic solvents and emulsifying agents, representing an important advance from a green chemistry perspective. In addition, it was demonstrated that the drying method significantly influences the final properties of the microcapsules: compared with lyophilization, vacuum drying led to more compact structures with superior mechanical stability and an increased capacity to protect curcumin against photoinduced degradation (Fig. 7). These characteristics were associated with more prolonged release of the active compound and expanded therapeutic potential.

The association of chitosan with polyanionic agents, such as sodium tripolyphosphate (S-TPP), has demonstrated considerable efficiency in the nanoencapsulation of curcumin via the ionotropic gelation technique [103]. Stabilization of the chitosan network through electrostatic interactions, without the use of organic solvents, led to the formation of homogeneous and stable nanoparticles, thereby optimizing both the encapsulation process and the release of the active compound. However, increasing the content of *Curcuma longa* essential oil resulted in a progressive increase in the particle size and a decrease in the encapsulation efficiency, despite a higher loading capacity. This highlights a limitation of the method, being particularly relevant for applications requiring strict dose control.

A different approach was proposed by Aguilera et al. [104], who combined nanoprecipitation with ionotropic gelation for the formulation of curcumin-loaded microcapsules based on chitosan and S-TPP. This strategy enabled efficient initial dispersion of curcumin, followed by particle stabilization through electrostatic interactions, yielding microcapsules with very high encapsulation efficiencies and long-term stability in solution. Compared with the conventional ionotropic gelation method, this approach provides improved control over particle size and a more balanced release profile of curcumin, although it involves greater technological complexity.

Even higher encapsulation efficiencies were reported by Gomathi et al. [105], through the introduction of histidine as an additional functional agent in chitosan–TPP-based systems. The role of histidine in nanoparticle stabilization is attributed to its imidazole group, which facilitates both electrostatic interactions and hydrogen bonding within the chitosan matrix [106]. This structural modification resulted in nanoparticles with enhanced stability, controlled release, and improved curcumin bioavailability, features that are particularly relevant for anticancer applications. Moreover, histidine-mediated facilitation of cellular internalization via endocytosis represents a clear functional advantage over unmodified systems, as confirmed by in vitro results [105].

In a comparative study, Schlichting et al. [107] investigated nanoparticles obtained from chitosan and hydroxypropyl methylcellulose acetate succinate using the same encapsulation technique but without additional modifying agents. Although the system exhibited very high encapsulation efficiency and good stability, the slightly larger particle size may limit cellular penetration. In comparison with histidine-modified nanoparticles, the absence of a functional agent may reduce the bioavailability of curcumin, despite the favorable structural stability.

Beyond oncological applications, curcumin-loaded microcapsules have demonstrated significant potential in tissue regeneration and epithelialization. A 2022 study [108] highlighted the effectiveness of a composite hydrogel based on chitosan succinate and fish collagen, which provides a porous and bioactive matrix conducive to absorption and tissue regeneration. Compared with other similar systems, the combination of the two biopolymers enabled prolonged curcumin release, while fish collagen actively contributed to stimulating natural healing processes.

**Fig. 8:**
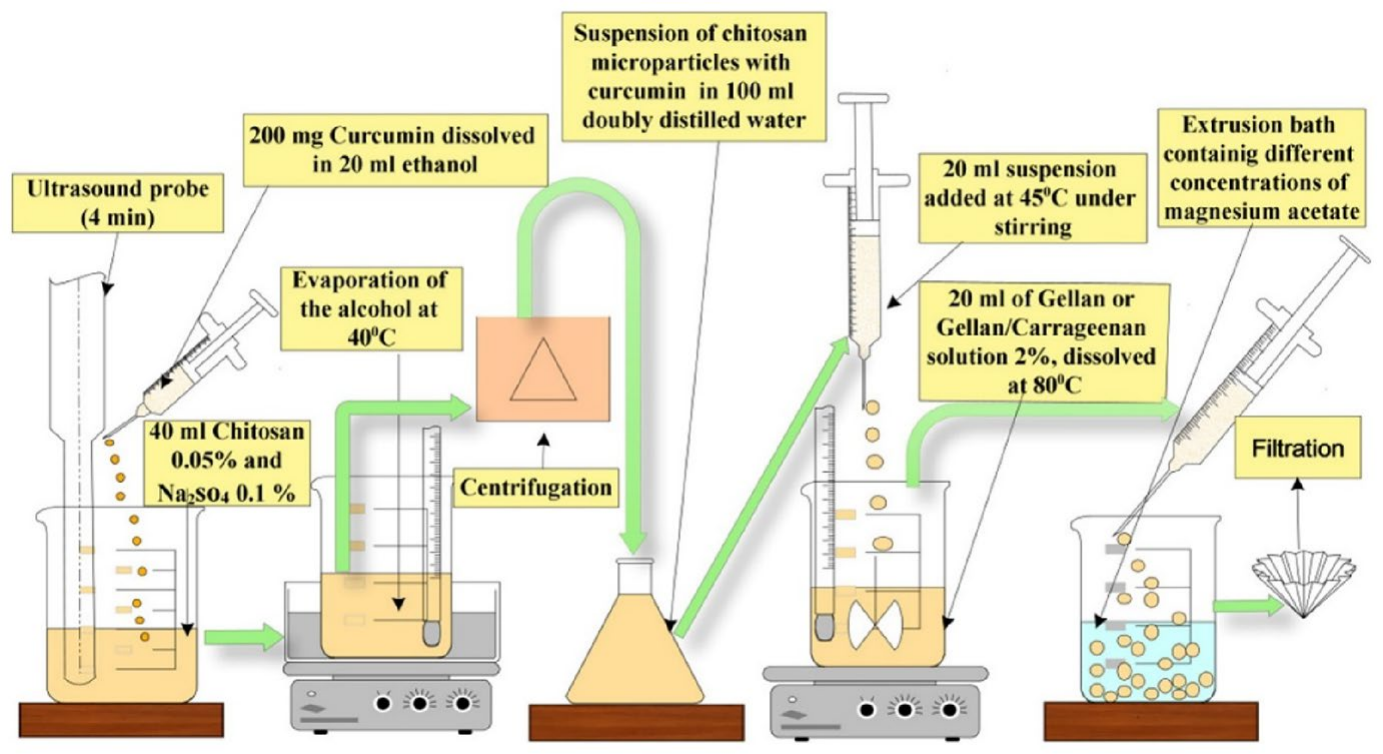
Schematic representation of the process of obtaining polysaccharide composites CompPs with immobilized curcumin [109]

Another complex system was developed for targeted delivery of curcumin to the colon [109], using natural polymers through polyelectrolyte complexation and ionotropic gelation. The presence of *ι*-carrageenan significantly influenced the porosity and release behavior, promoting curcumin release at neutral pH while protecting the compound in the gastric environment. These findings emphasize the importance of polymer selection based on the biological target, rather than solely on encapsulation efficiency. The microcapsule preparation scheme is shown in Fig. 8.

Although chitosan is among the natural polymers that are most widely used for ionotropic gelation, other materials such as alginate have proven to be suitable alternatives [110, 111]. A recent comparative study [112] showed that alginate-based nanoparticles provide considerable protection of curcumin in the gastric environment and pH-dependent controlled release, although their colloidal stability is lower. While both systems significantly enhanced the bioavailability of curcumin, alginate was found to be more suitable for food and pharmaceutical applications requiring gastric protection.

Similarly, Nguyen et al. [113] demonstrated that pectin is an effective carrier for curcumin microencapsulation, and that the use of different surfactants significantly influences the physical form and release behavior of the active compound. Although certain surfactants promoted rapid release by transforming curcumin into an amorphous form, antioxidant activity was preserved, indicating that compositional modifications can subtly modulate the solubility and bioavailability.

Finally, the applicability of curcumin microcapsules has been extended to the cosmetic field, where Ramya et al. [114] demonstrated that microparticles obtained by ionotropic gelation can be used as biodegradable alternatives to microplastics, while preserving the antimicrobial properties of curcumin. These findings highlight the versatility of ionotropic gelation techniques and their potential applications across multiple domains, ranging from therapy and nutrition to sustainable cosmetic products.

For a better understanding of the influence of materials and process conditions on the obtained microcapsules, Table 2 presents the main parameters related to the polymer, crosslinking agents, and homogenization conditions, as well as their effects on the characteristics of the final product.

**Table 2:** Comparison of important parameters for curcumin microencapsulation by ionotropic gelation

Polymer(s) used (conc. %)	Cross-linking agent	Zeta potential (mV)	Homogenization/centrifugation* speed and duration	Particle size	Yield (%)	Encapsulation efficiency (%)	Ref.
Chitosan 1.35% and alginate 2.145%	TPP 1.5%	−46.4	350 rpm for 40 min	551.1 nm		71. 828	[101]
Sodium alginate and chitosan	Calcium chloride 0.1 M	–	11,000 rpm for 60 min	4.69 ± 0.57 to 5.30 ± 0.38 mm for 0 wt % and 50 wt% content		–	[102]
Chitosan (16 mg dissolved in 8 mL acetic acid) Poloxamer 407 (0.5 g)	TPP (1 mL of 2 mg/mL solution)	–	–	122 ± 2 nm		96	[104]
Chitosan (50 mg in 10 mL of (2% v/v) acetic acid) and histidine (50 mg dissolved in 10 mL of double distilled)	TPP	–	12,000 rpm for 45 min	127.86 ± 44.344 nm		98.4	[105]
Chitosan 0.1% w/v	Hydroxypropylmethylcellulose acetate succinate 0.1% w/v	+16.6 ± 1.5	6000 rpm for 60 min	206 ± 1 nm		99.97	[107]
*N*-succinyl chitosan (10 mg/mL)	TPP (0.5%, 20 mL)	+14 + 19.7	10,000 rpm for 5 min	∼47 nm (^Z^-average diameter)		96.2 ± 2.3	[108]
Gellan, *ι*-carrageenan, and chitosan	Magnesium acetate Na_2_SO_4_ 0.1%	–	6000 rpm	2–3 μm		85.71—97.25	[109]
Alginate 2% (m/v) and chitosan; additional material: A-type zeolites	Calcium chloride 10% (m/v)	–	800 rpm	1.5–1.7 mm	51	–	[110]
Sodium alginate 3.5% (w/v), polyvinyl alcohol 3.5% (m/v)	Calcium chloride 5% (m/v)	–	–	1014.0 ± 0.3 to 1333.0 ± 0.3 mm	53.26 ± 2.52 to 66.57 ± 2.14	87.18 ± 2.14 to 92.35 ± 2.00	[111]
Alginate 1.0% (w/v) or chickpea protein	Calcium chloride 1.5% w/v	−15.40 ± 8.13 mV to 10.80 ± 6.56 mV	14,000 rpm for 20 min	522.9 to 867.4 nm	0.43 ± 0.001 to 0.32 ± 0.001	90.72 ± 0.35% to 93.02 ± 0.54%	[112]
Chitosan (0.02% w/v) and sodium alginate (0.01–0.06% w/v)	Tripolyphosphate calcium chloride	+12.05	6000 rpm for 45 min	272.9 nm		86.08–91.41	[116]
Quaternized aminated chitosan (0.2%; w/v)	Tripolyphosphate (0.1%; w/v)	+48.3	*12,000 rpm for 20 min	162 ± 9.10 nm		94.4 ± 0.91	[117]
Chitosan (0.5 g) dissolved in 50 mL of an acetic acid solution (2%, v/v)	Tripolyphosphate 2 wt%, tripolyphosphate 5 wt%, or NaOH in EtOH 4 wt%	–	550 rpm for 30 min 1 h (rt)	1 mm 2 mm		95.9 91.4 99.8	[118]
Chitosan (0.5% w/w)	TPP solution (0.125% w/v)	+25.8	*10,000 rpm for 1 h	229 nm		59.3 ± 2.98	[119]
Chitosan 5 mg, chitosan 6 mg	TPP 0.5 mg, TPP 1 mg	+ 37.7	*12,000 rpm for 30 min	481.7 nm 761.5 nm		45 65	[120]
Sodium alginate 2%, chitosan 1%	Calcium chloride 0.2 M	–	500 rpm for 15 min	–		89.86	[121]

Ionotropic gelation proves to be a versatile and safe method for curcumin encapsulation, as it uses natural, biocompatible polymers, and nontoxic crosslinking ions, which explains why the reported encapsulation efficiency frequently exceeds 90% and the stability of the particles is maintained over the long term. The size, stability, and encapsulation efficiency of curcumin are influenced by the concentration of polymers (chitosan, alginate, and pectin), the type and concentration of crosslinking agent (CaCl_2_, TPP, sodium sulfate), the polymer-to-curcumin ratio, as well as process parameters such as pH, speed, and duration of homogenization, the drying method (lyophilization versus vacuum drying), or even the use of copolymers and additives (histidine, surfactants, and DES). These factors determine the degree of porosity, zeta potential, mechanical strength, and curcumin release rate, which explains why the same materials can generate microcapsules with different performances depending on the processing method used. The association of chitosan with other polymers (alginate, histidine, and collagen), or with complementary techniques (nanoprecipitation or use of eutectic solvents) results in significant improvements in terms of size, dispersion, and release control, confirming that the method enables adaptation of the final characteristics of the microcapsules to diverse applications, from anticancer therapy to cosmetics.

In addition, recent studies have shown that the microencapsulation of curcumin by spherification leads to the maintenance of color intensity and the reduction of discoloration phenomena induced by various factors, such as those mentioned above [115]. Thus, it can be concluded that, by using this technique, the incorporation of curcumin can be achieved without affecting the visual properties, and the trichromatic parameters (L*, A*, B*) in the CIELAB system constitute an indicator of the encapsulation efficiency and the physical and chemical stability of the formulations obtained under ambient conditions.

Despite its many advantages, ionotropic gelation is not without limitations. The relatively high water content of gelled systems can affect the long-term stability, and if the surface charge is insufficient to ensure colloidal repulsion, particle aggregation may occur. Furthermore, scale-up can be challenging owing to sensitivity to mixing conditions and ion diffusion rates. When more rigorous interfacial control and a well-defined core–shell architecture are required, beyond what ionotropic gelation can provide, complex coacervation emerges as a relevant option. These limitations have motivated the exploration of hybrid strategies that combine ionotropic gelation with complementary techniques, such as nanoprecipitation, multilayer coating, or controlled drying methods, to increase the stability and reproducibility. In this way, more stable curcumin-containing nanoparticles with precise release control and cellular targeting can be obtained, thus transforming curcumin from an unstable compound into a functional nanomaterial.

Ionotropic gelation thereby represents a highly versatile and gentle encapsulation approach for curcumin, offering excellent protection, high encapsulation efficiency, and tunable release characteristics. Its effectiveness strongly depends on the polymer selection and formulation design, highlighting the importance of understanding the molecular-level interactions between curcumin and carrier materials. In this context, molecular docking and molecular dynamics simulations can provide valuable insights into polymer–curcumin affinity and network stability, supporting the rational optimization of ionotropically gelled delivery systems.

### Encapsulation by Coacervation

**Fig. 9:**
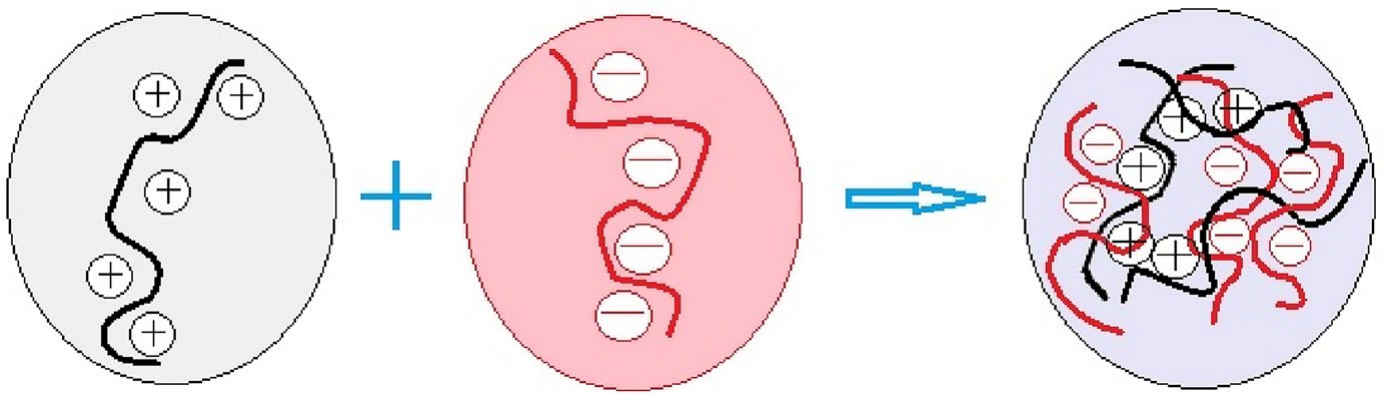
Formation of polyelectrolyte complex by cation–anion interaction

**Fig. 10:**
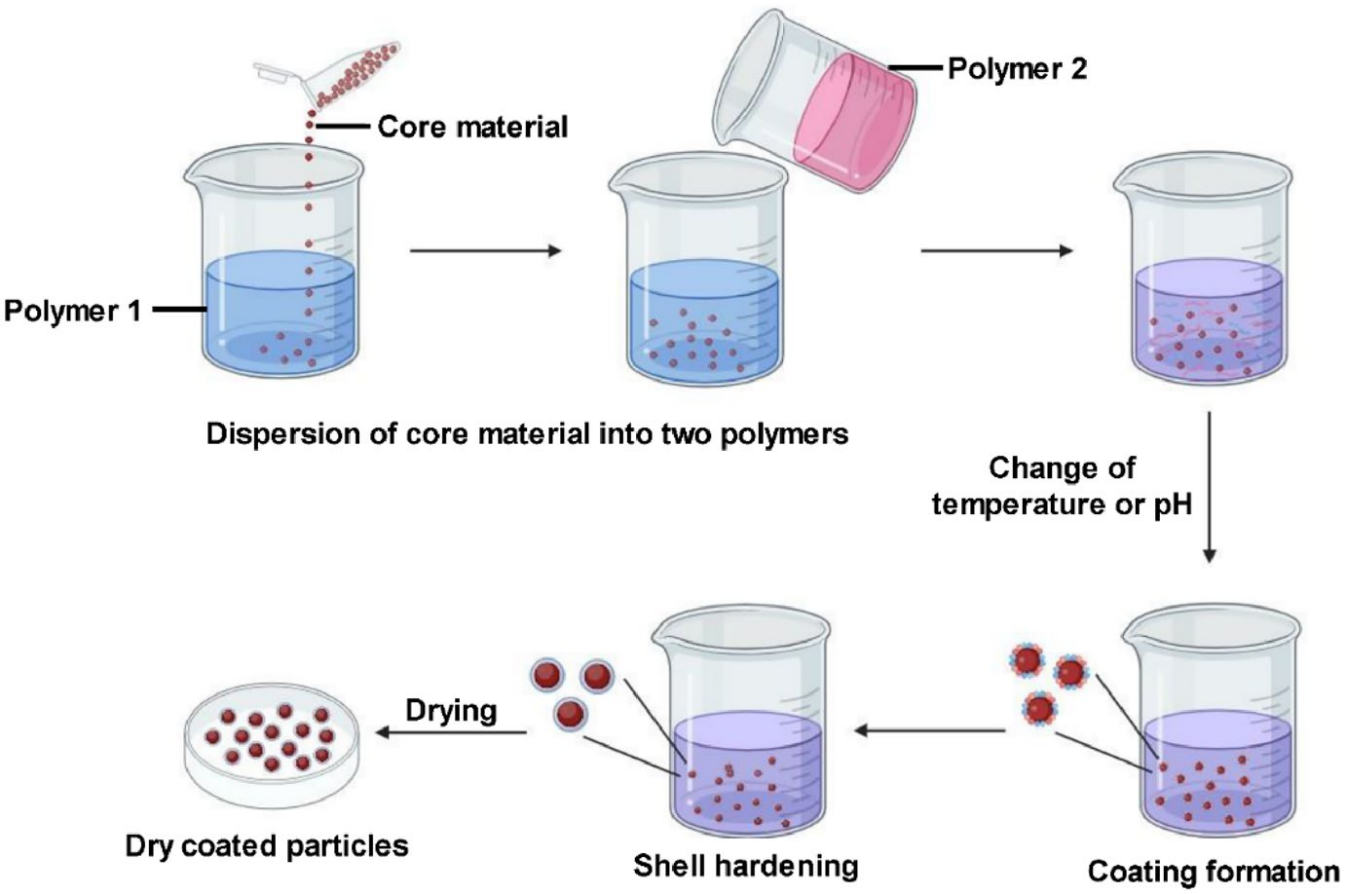
Experimental routes of complex coacervation [122]

Coacervation-based encapsulation has attracted increasing interest for curcumin delivery because it enables the formation of polymer-rich phases capable of efficiently entrapping hydrophobic molecules under relatively mild aqueous conditions [58, 122]. The general principles of coacervate formation and the typical processing routes are illustrated schematically in Figs. 9 and 10, while the following discussion focuses on the formulation-dependent performance and recent advances in curcumin stabilization. Although early descriptions of coacervation were based on classical electrostatic models, contemporary studies indicate that the encapsulation efficiency, structural stability, and release behavior are strongly influenced by the polymer composition, environmental conditions, and post-formation stabilization strategies applied. In particular, complex coacervation systems involving proteins and polysaccharides have demonstrated improved protection of curcumin against degradation and enhanced control over its release profile. However, the sensitivity of coacervate formation to pH, ionic strength, and polymer ratio remains a critical limitation that requires careful optimization when designing delivery systems for specific pharmaceutical or food applications.

Complex coacervation is widely employed in pharmaceutical formulations to obtain micro- and nanoscale encapsulated systems with controlled properties. Applied to curcumin, this technique enables the fine regulation of biopolymer interactions and release behavior under physiological conditions, thereby enhancing the stability and bioavailability. Recent studies further reveal marked differences between polymer–polymer, protein–protein, and protein–polysaccharide coacervates, particularly in terms of their encapsulation efficiency, structural stability, and biological functionality.

For example, chitosan–alginate-based systems [123] demonstrate that electrostatically driven interactions between oppositely charged biopolymers are sufficient to form stable coacervates, achieving satisfactory encapsulation efficiencies and sustained, pH-dependent release of curcumin. The main advantage lies in their formulation simplicity and the exclusive use of natural polymers, making them particularly attractive for food applications. However, their pronounced pH sensitivity and extensive swelling under alkaline conditions may limit precise dose control, especially in pharmaceutical applications that require tightly regulated release kinetics.

**Fig. 11:**
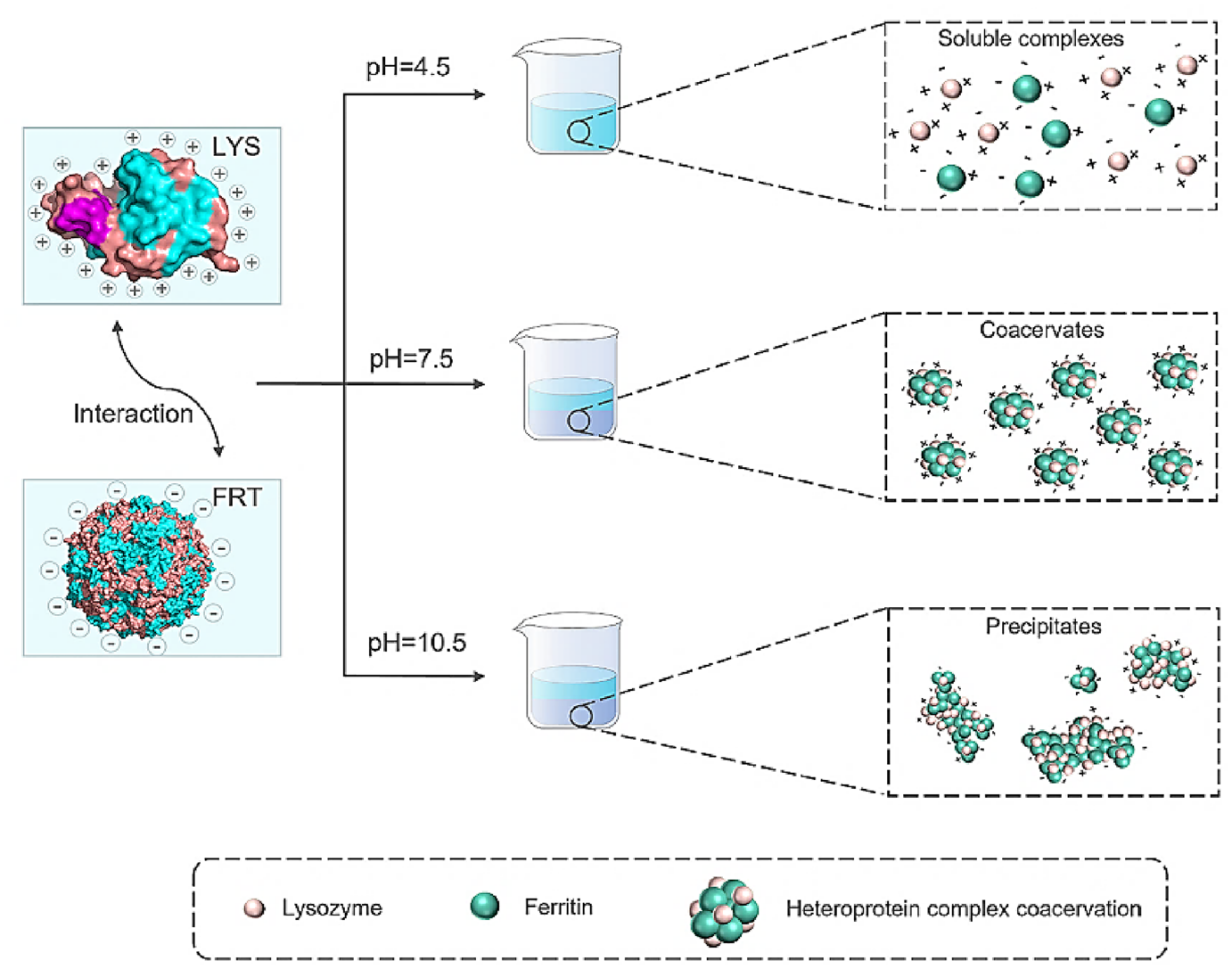
Schematic representation of the mechanism underlying the interaction between ferritin (FRT) and lysozyme (LYS) in different phases [125]

In contrast, multicomponent systems based on chitosan and caseinate [124] provide more precise control over the particle architecture and enable co-encapsulation of both hydrophilic and hydrophobic compounds. While this approach offers clear functional advantages, it is associated with increased technological complexity, which may hinder reproducibility and large-scale production. Consequently, improvements in encapsulation efficiency and stability must be carefully weighed against process feasibility. Protein–protein coacervates, exemplified by ferritin–lysozyme systems [125], are distinguished by their ability to confer exceptional stability to curcumin against degradation factors such as heat and ultraviolet (UV) radiation. These systems are particularly promising for pharmaceutical and nutraceutical applications with stringent stability requirements. Nevertheless, their strong dependence on precise pH conditions and on the native structure of the proteins, together with the cost of purified protein components, may limit their broader industrial applicability. Figure 11 illustrates the mechanism of interaction between ferritin and lysozyme, highlighting that, at low pH, the disrupted protein structure prevents complex formation, while at high pH, the opposite charges favor electrostatic complexation. At pH 7.5, the two proteins form a heteroprotein coacervate stabilized by electrostatic, hydrophobic, and hydrogen-bonding interactions.

Protein–polysaccharide systems [126, 127] offer an attractive balance between stability, efficiency, and practical applicability. Compared with purely protein-based coacervates, they exhibit enhanced resistance to gastrointestinal digestion and greater compatibility with real food matrices, such as baked products. Their performance, however, is highly dependent on the formulation parameters and post-coacervation stabilization methods used, requiring careful optimization for each specific application. A distinct direction is represented by Pickering emulsion gels stabilized by coacervates [128, 129], which introduce an additional functional dimension by modulating the lipid digestion and increasing the bioaccessibility of curcumin. These systems extend beyond the conventional protective role of microcapsules. However, their structural complexity and the need for precise interfacial control may restrict short-term industrial implementation. On the other hand, induced coacervation of short peptides [129] opens new perspectives for the development of minimal, easily tunable systems with intrinsic antioxidant properties. Although conceptually promising, these approaches are in the early stages and require further validation with respect to safety, long-term stability, and practical applicability.

A comparative analysis of the available studies indicates that no universally optimal coacervation system exists for curcumin encapsulation. The choice of strategy should be guided by the intended application, balancing encapsulation efficiency, stability, release control, and technical feasibility. This methodological diversity underlines the adaptability of complex coacervation as a delivery platform, while also highlighting the need for a critical, context-dependent approach in the design of curcumin delivery systems. To synthesize and compare the efficiency of the different coacervation methods applied in the reviewed studies, with a focus on the materials used and the key results obtained, they are summarized in Table 3.

**Table 3:** Variation of parameters and performance of coacervation encapsulation

Coacervation type	Materials forming the coacervate	Optimal pH for coacervation	Zeta potential (mV)	Encapsulation efficiency (%)	Loading capacity/loading efficiency (%)	Yield (%)	Particle size	Ref.
Complex coacervation	Caseinates, chitosan	5.0	+28	96.2	4.8%	–	582 nm	[124]
Complex coacervation	Ferritin, lysozyme	7.5	~ 0	28.66 ± 0.87	–	–	82.2–188.3 nm	[125]
Complex coacervation	Zein hydrolysates, chitin nanocrystals	5.0	+15–30	~ 90	–	–	–	[127]
Complex coacervation	Ovalbumin, pectin	2.0	~ 0	–	–	90.9	21.3 ± 2.3 μm	[128]
Simple coacervation	Tailoring peptide, buffer buffer, NaCl	10.5 4.8	–	96	–	–	20 μm 50 μm	[129]
Complex coacervation	Carboxymethylated tara gum, lysozyme	5.0	positive	74.86	0.26 ± 0.06%	–	–	[130]
Complex coacervation	Succinyl mung bean protein Arabica gum	3.0	-16.1 ± 0.86	99.79 ± 0.03	24.94 ± 0.05 μg⋅mg	–	4026.66 ± 106.34 nm	[131]
Complex coacervation	Pennisetin, casein	5.0	–	68	17%	–	2.5 to 5.27 μm	[132]
Complex coacervation	Bovine serum albumin and poly-d-lysine with low and high molecular weights, NaCl	7.0	+55 ± 2.9 mV	60	22 mg/mg	–	316 ± 43 nm	[133]
Complex coacervation	Gelatin B, chitosan, formaldehyde for cross-linking	5.5	–	82.69 ± 0.93	16.21 ± 0.22%	87.89 ± 2.57	40.498 ± 0.175 µm	[134]
Complex coacervation	*Lallemantia iberica* seed gum, chitosan	4–7	+31.6 ± 2.3	97.3	–	–	150.8 ± 29.9 nm	[135]
Complex coacervation	Zein, chitosan	4.0	–	95	8.10%	–	< 400 nm	[136]
Complex coacervation	Lysozyme, tripolyphosphate	9.0	~ 0	80–90	–	81	Microsized droplets	[137]
Complex coacervation	β-Conglycinin and lysozyme	6.0 (liquid phase) 7.0–8.0 (solid phase)	~ 0	96.4 98.4	410–486 μg/mg	91.4	Micron-scale precipitates	[138]

Coacervation, especially complex methods, offers significant advantages as demonstrated by reported studies. The formation of a dense and uniform polymeric shell from natural biopolymers, such as proteins and modified polysaccharides, provides superior protection of curcumin and achieves very high encapsulation efficiencies, up to 96%. Another benefit is the controlled release, which can be adjusted according to pH to ensure optimized bioaccessibility (over 80% in some Pickering systems), but also the material versatility, allowing the use of new systems, such as short peptides or nongluten proteins, such as penicetin, which can be synthesized cheaply and self-assemble spontaneously. An important challenge is the dependence of the coacervate formation on process parameters (pH, ionic strength, and polymer ratio), requiring rigorous optimization, since the theoretical mechanism (based on the entropy of counterion release) is not fully modeled. Also, achieving long-term stability often requires post-coacervation steps (crosslinking or solidification), which increase the time and complexity of the process. Future research should also pay attention to formulating coacervates with integrated properties (thermal and mechanical resistance) that eliminate the need for chemical crosslinking, and on the use of fluorescent peptides as dual vehicles that not only encapsulate but also allow for real-time visual tracking of delivery.

Compared with spray drying and ionotropic gelation, coacervation provides superior control over capsule architecture and interfacial properties, but may require more precise control of formulation conditions. Owing to the specific characteristics of the polymer coating, microencapsulation by coacervation generally offers superior color protection of bioactive substances, such as curcumin, compared with the above-mentioned techniques. In the case of coacervation, the matrix acts more effectively as a barrier against oxidation and photodegradation, thus presenting improved color stability. As a result, coacervation is particularly attractive for high-value applications, where precise release behavior and increased protection justify the additional formulation complexity. Like other encapsulation techniques, molecular docking and molecular dynamics simulations can provide valuable insights into polymer–curcumin interactions and interfacial stability, supporting the rational selection of polymer pairs and optimization of coacervate systems.

### Extrusion Encapsulation

**Fig. 12:**
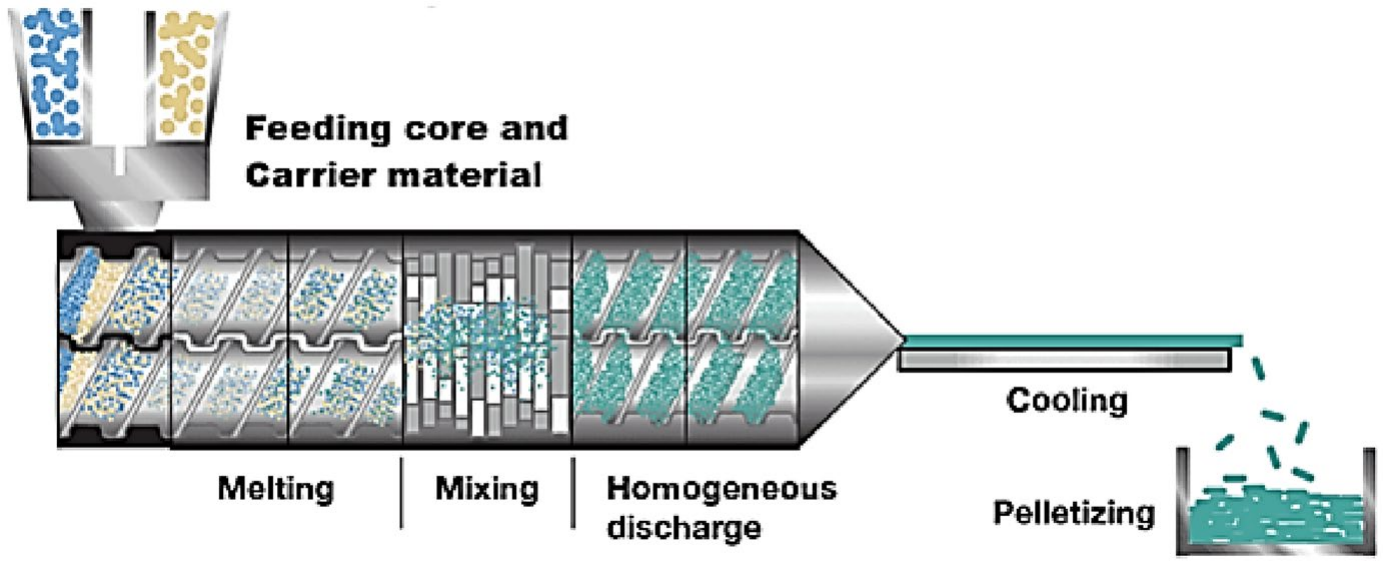
Hot-melt extrusion apparatus for encapsulating bioactive compounds [141]

Extrusion-based encapsulation has been investigated as an alternative strategy for producing robust curcumin delivery systems through the formation of solid matrices under controlled thermal and mechanical conditions [139, 140]. The general operating principle of extrusion technologies is illustrated in Fig. 12 [141]. Recent research has focused primarily on optimizing the processing temperature, matrix composition, and residence time to limit thermal degradation and improve the encapsulation efficiency. Studies employing hot-melt extrusion and related approaches indicate that extrusion can provide high production yield, good structural reproducibility, and the possibility of tailoring the release behavior via matrix design.

Particle formation techniques based on melting processes or supercritical fluids have emerged as effective alternatives for curcumin encapsulation, offering reduced-solvent or even solvent-free approaches, with high loading efficiencies and improved stability. Among these, the particles from gas-saturated solutions (PGSS) process has shown promising results for stabilizing curcumin in solid–lipid particles [142]. This method enabled the production of homogeneous microparticles with high curcumin content and minimal degradation, highlighting its relevance for thermosensitive bioactive compounds. However, the study also indicated that the particle quality largely depends on the formulation choices, particularly the presence of residual organic solvents and auxiliary gases, which can influence the aggregation and encapsulation efficiency. These findings suggest that, although PGSS is a robust method, careful optimization is required to balance particle homogeneity and formulation complexity.

Hot-melt extrusion (HME) represents another widely investigated strategy, particularly for improving curcumin dissolution behavior through amorphization. The work of Althobaiti et al. [143] illustrates the potential of HME to significantly enhance curcumin release when combined with suitable polymeric carriers, while also ensuring short- to medium-term physical stability. However, this approach is hindered by the thermal sensitivity of curcumin and the limited loading capacity of the carrier polymer, as excessive loading compromises system homogeneity. These observations underscore a common trade-off in HME: improvements in bioavailability are often achieved at the expense of formulation flexibility.

Similar conclusions can be drawn from studies using twin-screw extrusion with methacrylate-based carriers [144]. In this case, HME enabled the production of nanoscale curcumin formulations with enhanced bioavailability, confirming the effectiveness of extrusion-based technologies in improving systemic exposure. At the same time, comparisons with alternative processing conditions reported in the literature [145] indicate that the extrusion performance is highly sensitive to the processing intensity and thermal management. While more aggressive processing can further increase the absorption of curcumin, it also raises the risk of thermal degradation and batch-to-batch variability if the temperature control is not sufficiently rigorous.

The parameters used and results obtained within the analyzed studies on different extrusion methods for curcumin encapsulation are presented in Table 5, which shows that no studies were identified using other extrusion methods for curcumin encapsulation apart from melt extrusion and PGSS, possibly owing to the technical complexity and difficulties in monitoring the process.

**Table 5:** Variation of processing conditions and results of extrusion encapsulation methods

Extrusion type	Materials used in extrusion	Extrusion temperature (°C)	Gas pressure in mixing chamber	Screw speed (rpm)	Encapsulation efficiency (%)	Loading capacity/loading efficiency (%)	Yield (%)	Particle size	Ref.
PGSS	Tristearin + soy phosphatidylcholine, dimethylsulfoxide	60 °C	130 bar	Not applicable	–	–	30‒87	< 20 mm	[142]
HME	Soluplus^®^	130 °C 140 °C	Not applicable	75	–	45%	–	–	[143]
HME	Eudragit^®^ EPO	< 135 °C	Not applicable	30	–	–	–	50–150 nm	[144]
HME	Eudragit^®^ EPO	130–160 °C	Not applicable	100	–	–	–	–	[145]
HME	Eudragit RS/RL Porogen	145 °C	Not applicable	80	99.53–100.54	98–101	–	The optimal particle size was the fraction passing through an 80-mesh screen	[146]

Extrusion is a versatile and efficient technique for encapsulating curcumin. The HME method, using a hot twin screw, is well documented and optimized but requires a fine balance between process parameters to avoid the thermal degradation of curcumin. Other screwless methods, such as melt injection, centrifugal extrusion, or electrostatic extrusion, are innovative technologies that could offer additional benefits, but these are less studied owing to the complexity of the equipment and the challenges related to precise process control and stability of the active substance. A concrete and feasible solution to improve curcumin encapsulation could be the development of a hybrid method that combines the flexibility and efficiency of hot extrusion with the advantages of screwless methods, such as melt injection or PGSS. This would involve the use of a twin screw extruder to plasticize the material and homogenize the curcumin, followed by rapid and controlled cooling by injection into a cold antisolvent to prevent thermal degradation and obtain stable, amorphous particles with increased bioavailability. In addition, the integration of a supercritical CO_2_ flow, specific to PGSS, could help in the formation of uniform and well-charged particles.

The reviewed studies thereby indicate that extrusion represents a robust and versatile encapsulation technique for curcumin, being particularly suitable for applications where gentle processing, mechanical stability, and controlled release are prioritized. Its effectiveness depends strongly on the polymer selection and matrix architecture, underscoring the importance of understanding polymer–curcumin interactions at both the macroscopic and molecular levels. Like other encapsulation strategies, insights from molecular docking and molecular dynamics simulations can support the rational selection of polymeric carriers and predict curcumin retention and release behavior within extruded matrices.

### Systems Based on Inorganic Layers (Layered Double Hydroxides (LDH))

When the primary objective is to ensure both chemical and thermal protection while achieving a pH-responsive curcumin release mechanism, inorganic layered double hydroxide (LDH) systems stand out by offering a delivery paradigm distinct from that of conventional organic carriers. The encapsulation method based on LDHs, also known as “anionic clays,” represents an effective solution to avoid the limitations of the low stability and solubility of curcumin. The method is based on the unique crystalline architecture of LDHs. These inorganic compounds have a lamellar structure, similar to that of brucite, but their layers are positively charged owing to the isomorphic substitution of divalent cations, such as Mg^2+^, with trivalent cations, such as Al^3+^. This positive charge of the layers is neutralized by the presence of anions and water molecules in the expandable interlayer space [147, 148].

**Fig. 13:**
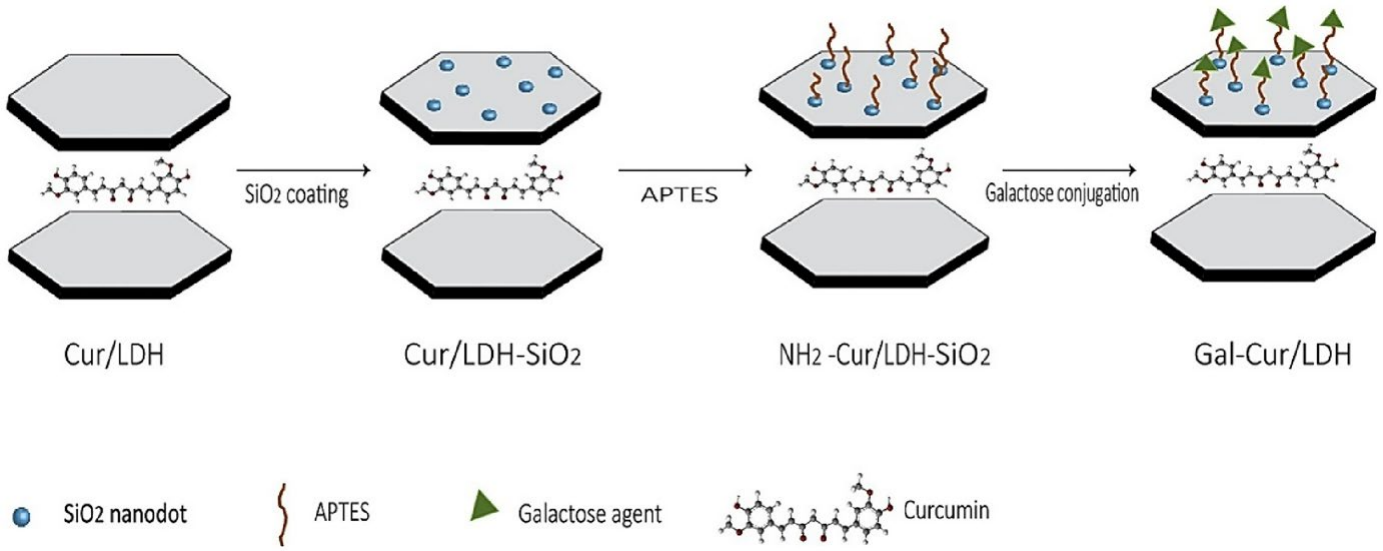
Schematic representation of the preparation of Gal-Cur/LDH nanoparticles, obtained by Cur/LDH synthesis via hydrothermal coprecipitation, followed by SiO_2_ coating to facilitate aminolysis with (3-aminopropyl)triethoxysilane (APTES) and, finally, conjugation of Gal to the resulting NH_2_ groups [147]

The mechanism of curcumin encapsulation in LDH structures is based on its ability to interact with the anionic interlayer spaces of the material. Although curcumin is neutral under physiological conditions, in mildly basic environments it can form anionic species, thereby facilitating intercalation into the LDH structure. This approach was exploited in a study that employed hydrothermally assisted coprecipitation to develop a targeted curcumin delivery system [147]. The resulting system exhibited moderate loading efficiency and good structural stability, while subsequent functionalization with galactose enabled specific recognition of asialoglycoprotein (ASGP) receptors overexpressed on hepatocellular carcinoma cells. From a functional perspective, this strategy highlights the benefit of selective tumor targeting, enhancing therapeutic efficacy while reducing nonspecific cytotoxic effects. However, the fabrication of this system involves multiple sequential steps, including inorganic coating and chemical functionalization, which increases technological complexity and may restrict reproducibility and scalability. The multistep construction of the Gal–Cur/LDH system, encompassing LDH intercalation, silica coating, amine functionalization, and final galactose conjugation, is illustrated schematically in Fig. 13 [147].

A more recent approach aimed to increase the curcumin loading and integrate combination therapy by co-encapsulating curcumin and doxorubicin in functionalized LDH systems [149]. Compared with single-component systems, this strategy achieved slightly higher loading efficiencies and introduced an intelligent, tumor-responsive release mechanism triggered by the acidic tumor microenvironment. The use of Schiff base linkages provided stability under systemic circulation conditions while enabling rapid drug release at the tumor site, representing a clear advantage for oncological applications. However, the incorporation of multiple functional components increases the complexity of the formulation and may amplify the variability in the final system.

In contrast to ligand-based targeting strategies, another study demonstrated that exfoliated LDH nanoparticles loaded with curcumin can retain high therapeutic efficacy even in the absence of a specific targeting ligand [150]. In this case, the release mechanism is primarily driven by nanoparticle degradation under acidic tumor conditions, leading to preferential curcumin release within the diseased tissue. This approach substantially reduces the complexity of the system and suggests that, in certain therapeutic contexts, physicochemical control of nanoparticle stability may be sufficient to achieve optimal therapeutic outcomes.

A distinct direction is represented by “green” mechanochemical methods, which completely avoid coprecipitation and functionalization steps [151]. By reducing the synthesis to a single-step process, these methods offer clear advantages in terms of sustainability and technological simplicity. Unlike LDH systems developed for oncological applications, which are optimized for rapid, pH-triggered release, mechanochemically produced nanohybrids were designed for transdermal applications, exhibiting enhanced thermal stability and slow, sustained release profiles. This distinction underscores the adaptability of the LDH platform and emphasizes that the release mechanism must be carefully aligned with the intended therapeutic purpose.

The analyzed studies demonstrate that LDH can be transformed from a simple carrier material into a functional delivery system through diverse formulation strategies. The differences among these approaches extend beyond synthesis methods to include release activation mechanisms and overall system complexity. Consequently, the optimal strategy should be selected on the basis of the target application, requiring a balance between therapeutic efficiency, release control, and technological feasibility.

Table 6 summarizes how researchers have adapted LDH chemistry (through the choice of M^2+^ cations, functionalization, and addition of ligands) to obtain a wide spectrum of results: from rapid, targeted, and pH-dependent release for cancer therapy, to slow and sustained release for dermatological antimicrobial applications, also highlighting the effectiveness of stabilizing curcumin in the LDH matrix. LDH-based systems offer an efficient solution for encapsulating curcumin, based on an inorganic matrix and strictly pH-controlled electrostatic interactions, overcoming some of the limitations of polymer-based methods. The advantage is that LDHs are not only vehicles but also intelligent delivery platforms. Their lamellar architecture provides superior physical and thermal protection for curcumin, eliminating the risk of thermal degradation as in the case of spray drying. The key innovation is the post-synthetic functionalization (e.g., with galactose or lactose), which transforms LDHs into active targeting systems, ensuring specific recognition of receptors on cancer cells. However, their major limitation is reduced industrial feasibility. LDH synthesis is laborious, difficult to scale, and requires stringent conditions, while the loading efficiency is modest (about 30%). Also, the long-term stability and the potential risk of systemic toxicity of metal cations raise questions. This risk arises from the fact that these materials are sensitive to acidic environments and may release ions, which can be therapeutically beneficial (e.g., pH responsiveness) but may also raise concerns regarding systemic toxicity, oxidative stress, or interference with metal homeostasis, particularly when redox-active ions (such as Cu or Fe) are employed, or when higher doses are administered [152]. In addition, there are studies indicating that aggregation of certain LDH-based formulations may exacerbate systemic toxicity [153], suggesting that colloidal stability and control over biodistribution and clearance are critical determinants of safety. An in vitro study demonstrated that curcumin-loaded LDH nanoparticles can exhibit higher cytotoxicity toward Caco2 epithelial cells compared with free curcumin, depending on the cationic composition of the LDH (e.g., Mg versus Zn), suggesting that the carrier matrix itself influences the biological effects of curcumin [154]. In local in vivo models (e.g., intramuscular implantation or dermal applications), LDH–curcumin nanocomposites have demonstrated biocompatibility and therapeutic potential without evident signs of local inflammation or acute tissue damage. However, these studies did not assess systemic effects or long-term safety [155]. Nevertheless, these risks can be mitigated by selecting cations with more favorable toxicological profiles, restricting the use of redox-active dopants to indications where the therapeutic benefit justifies the risk, applying surface coatings or functionalization strategies to limit uncontrolled ion release, and most critically, optimizing the colloidal stability to prevent aggregation and nonspecific retention [156].

**Table 6:** Synthesis of LDHs, curcumin loading methods, and main characteristics of the materials reported in the literature

LDH synthesis	Curcumin loading into LDH	LDH functionalization	Ligand	Zeta potential (mV)	Loading amount/loading capacity (%)	Encapsulation efficiency (%)	Particle size	Ref.
Mg–Al LDH synthesized by coprecipitation	Incorporated into LDH nanoparticles via coprecipitation intercalation	Functionalization performed with galactose, via SiO _2_ and APTES linking agents, using the coprecipitation hydrothermal treatment method	Galactosidase	+10.0 ± 0.7	31 1.5	–	116.1 ± 35.9 nm	[147]
Al–Cu LDH synthesized by coprecipitation	Loading onto the LDH surface via electrostatic interactions	Functionalization of LDH was achieved by sequential coating with amino-functionalized ZnO nanoparticles using APTMS, followed by grafting of the targeting ligand dialdehyde lactose onto the amine groups via a Schiff base reaction	Dialdehyde lactose	+21.3 ± 0.29	3.62 ± 0.05	36.2 ± 0.5	–	[149]
LDH synthesized by coprecipitation method. X-LDH NPs were made by dispersing the pristine LDH in formamide and stirring mechanically for 72 h	Addition of curcumin to aqueous X-LDH solution resulted in formation of strong bonds	Exfoliation achieved by dispersing LDH plates in formamide and mechanically stirring for 72 h to disassemble them into X-LDH nanosheets, thereby increasing the active surface area	–	+11.37 ± 0.68	27.66 ± 1.52%	~ 110 nm	–	[150]
Mg–Al–NO _3_ LDH was synthesized via a mechanochemical pathway	Curcumin was ground and incorporated with LDH to form a paste, and CC-LDH nanohybrids were obtained after drying	–	–	−23.1	8.2% w/w	–	604 nm	[151]
Mg-LDH and Zn-LDH synthesized by coprecipitation method	Curcumin incorporation into LDH via coprecipitation, which involved adding the curcumin solution to the metal salt solutions under pH control	–	–	−42.8 to 45.4	–	–	8 to 9 nm	[154]
Zn_3_Al-layered double hydroxide (LDH) by coprecipitation	PZn_3_Al-CR(Aq)–coprecipitation PZn_3_Al-CR(Et)–coprecipitation RZn_3_Al-CR(Aq)–reconstruction RZn_3_Al-CR(Et)–reconstruction	Intercalation of the curcumin anion into the interlayer space of the LDH metallic layers	Turmeric	–	–	–	16.66–35.6 nm	[157]

Compared with organic encapsulation techniques such as spray drying, ionotropic gelation, coacervation, or extrusion, LDH-based systems provide superior structural rigidity and resistance to thermal and chemical stress. However, these advantages are accompanied by challenges related to biocompatibility, long-term fate, and regulatory acceptance, particularly for food applications. As a result, LDH-based carriers are more commonly explored in pharmaceutical and biomedical contexts, where inorganic materials are already established in drug delivery and imaging applications. The performance of LDH-based curcumin systems strongly depends on molecular-level interactions between the curcumin, inorganic layers, and interlayer species. Parameters such as the layer composition, charge density, interlayer spacing, and surface functionalization govern the encapsulation efficiency, stability, and release behavior. Understanding these interactions is therefore important for rational system design. In this regard, molecular docking and molecular dynamics simulations provide valuable tools for elucidating curcumin–LDH interactions, predicting binding modes, and assessing structural stability within confined inorganic environments. Such in silico approaches can complement experimental studies by offering mechanistic insights into adsorption, intercalation, and release processes, thereby supporting the optimization of LDH-based delivery systems.

Systems based on layered double hydroxides are a promising inorganic alternative for curcumin encapsulation, particularly in applications requiring enhanced stability and controlled release. Their integration with organic components and predictive molecular modeling approaches can further expand their applicability and facilitate the development of hybrid delivery platforms that combine the advantages of inorganic robustness with organic versatility.

### Emulsion-Based Delivery Systems

As an alternative to solid-matrix-based strategies, which emphasize structural stability and controlled release, emulsion-based systems focus on the prior and optimized solubilization of curcumin within the lipid phase and on enhancing its bioaccessibility. The description of the previous techniques indicates that the incorporation of curcumin into the delivery system is affected by its hydrophobic nature and low solubility, as well as by the complex physiology of the human body. Thus, another strategy has focused on the use of oil-in-water (O/W) [158], water-in-oil (W/O) [159], Pickering [160], or multiple emulsions [161]. This approach involves dissolving curcumin inside an oil droplet, which is subsequently dispersed and stabilized in the aqueous phase by means of emulsifiers or nanoparticles [162]. Although emulsions are, from a thermodynamic point of view, unstable systems that risk separation over time (causing cremation or coalescence), optimizing their structure allows for kinetic stabilization. The diversity of nanotechnology has allowed the creation of several types of emulsified vehicles, from conventional emulsions to nanoemulsions (with radii of 50–200 nm) and Pickering emulsions, which use solid particles (not molecules) to create an irreversible mechanical barrier at the oil–water interface, providing superior physical stability [163, 164].

The success of this method depends on increasing the solubility of curcumin in the oil phase. For this purpose, the preparation of an auto-microemulsifying system has been explored as an effective strategy for encapsulating curcumin within nanometer-scale oil droplets, thereby improving its dispersion in aqueous environments [165]. By optimizing the combination of oil-dissolved curcumin with nonionic surfactants such as Tween 80 and ethanol, spontaneous self-emulsification upon contact with water was achieved, resulting in stable and transparent nanoemulsions. This approach provided a remarkable increase in curcumin solubility, approaching an order of magnitude compared with free oil, while simultaneously protecting the bioactive compound. The simplicity and efficiency of this system make it attractive, although its performance remains strongly dependent on the surfactant selection and formulation balance.

**Fig. 14:**
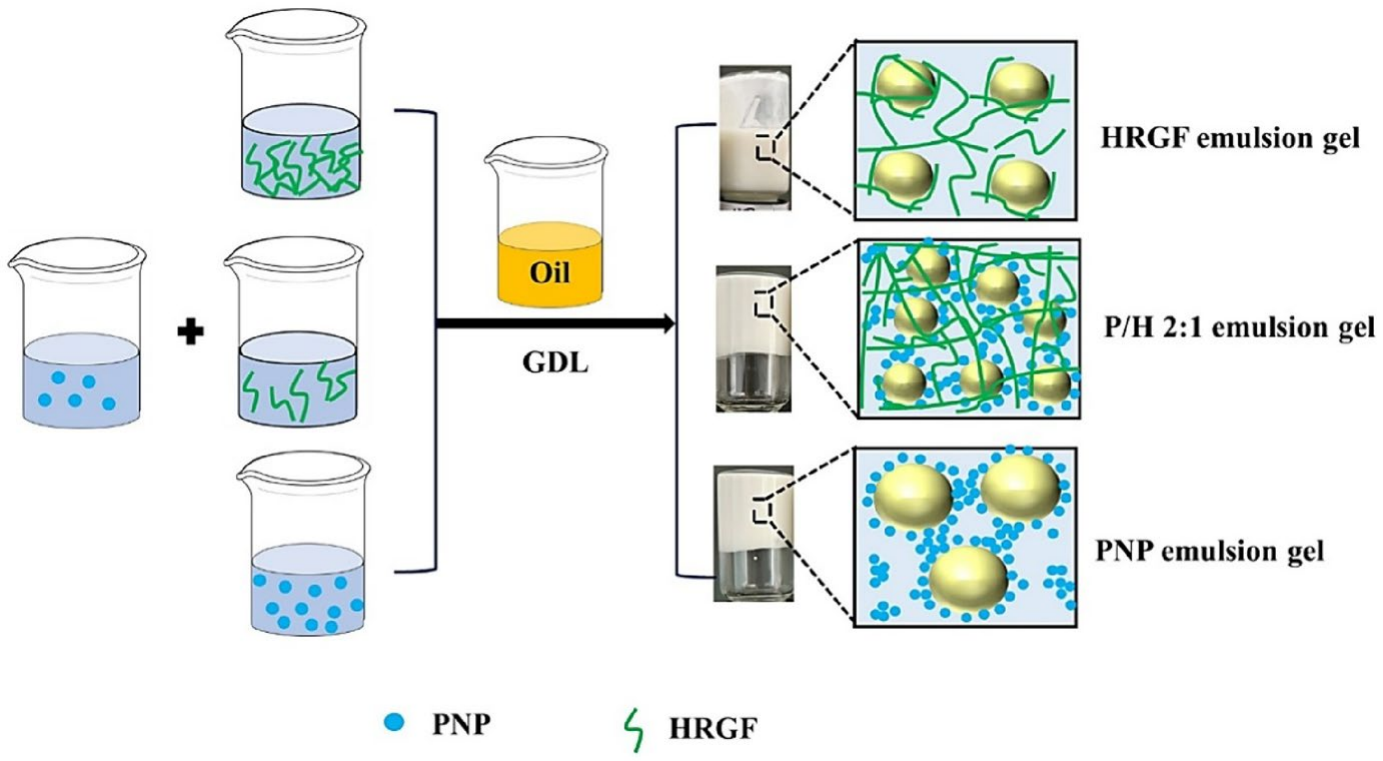
Schematic diagram of the formation mechanism of emulsion gels stabilized by PNP, P/H 2:1, and HRGFs [167]

Emulsion-based systems stabilized with biopolymers offer an alternative route, combining encapsulation efficiency with improved structural stability. In this context, emulsions formulated with debranched starch and lecithin achieved high stability and an encapsulation efficiency of approximately 71% [166], attributed to the ability of short starch chains to interact with hydrophobic molecules and stabilize the dispersed phase. Similarly, systems composed of pea protein nanoparticles (PNP) and hydrolyzed rice glutelin fibers (HRGFs) formed dense three-dimensional networks that effectively preserved the emulsion stability over prolonged periods and minimized curcumin degradation under gastric conditions, resulting in a bioaccessibility of approximately 59% [167]. Figure 14 illustrates the formation mechanism of gelled emulsions stabilized by pea protein nanoparticle (PNP) alone, by a 2:1 P/H mixture, and by hydrolyzed rice glutelin fibrils (HRGFs) alone, highlighting the distinct structure and composition of each type of gel.

On the other hand, the literature indicates that the solubility and stability of curcumin in emulsion systems are significantly influenced by the nature of the lipid phase, particularly the molecular weight and polarity of the oil used, as well as the overall processing conditions [168–170]. In this context, Joung et al. [171] demonstrated that emulsions formulated with short-chain triglycerides provide superior chemical stability of curcumin compared with those based on medium- or long-chain oils. This performance was attributed to the higher solubility of curcumin in short-chain oils, which ensures more effective protection within the internal phase. In contrast, oils rich in unsaturated fatty acids, such as corn oil, may accelerate curcumin degradation, likely owing to their susceptibility to oxidation. These observations highlight the importance of oil selection not only from a technological standpoint, but also from the perspective of the chemical stability of the bioactive compound. An alternative approach for protecting and controlling the release of curcumin is the use of cryogels, which can provide a stable three-dimensional matrix sensitive to pH variations.

The study conducted by Sowasod et al. [172] showed that cryogel systems derived from multilayer emulsions can modulate the swelling and release behavior of curcumin depending on the physiological environment. Specifically, these systems exhibited pronounced swelling under acidic conditions and a more compact structure at neutral or basic pH, promoting more controlled release. Although this strategy offers satisfactory functional control over the release profile, its efficiency depends on a fine balance between the polymer composition and processing conditions, which may limit its practical applicability on a large scale.

Multiple O/W/O emulsions represent another approach explored for curcumin encapsulation owing to their ability to separate the internal phase from the external medium. A recent study [161] demonstrated the feasibility of formulating such systems for curcumin incorporation; however, their medium-term stability remained a major challenge. The significant increase in particle size over time indicates a tendency for these emulsions to undergo coalescence and creaming, which may compromise the reproducibility and performance of the system. Thus, although multiple emulsions present high theoretical potential, stability-related limitations currently restrict their practical use.

Overall, these studies highlight that the efficiency of emulsion-based systems for curcumin delivery is strongly dependent on the choice of lipid phase and system architecture. Simpler solutions, based on suitable oils and conventional emulsions, may offer a better balance between stability and feasibility, while more complex systems allow superior functional control but at the cost of reduced stability and scalability. For a complete understanding of the results reported in the literature, essential parameters that influence the characteristics and performance of curcumin emulsions are presented in Table 7.

**Table 7:** Comparison between formulation parameters and physicochemical characteristics of emulsions used as curcumin delivery systems

Composition of oil phase	Composition of aqueous phase	Homogenization speed and time	Optimum pH	Zeta potential (mV)	Encapsulation efficiency (%)	Loading capacity/loading efficiency (%)	Particle size	Ref.
Extralight olive oil	WPI, sodium alginate, sodium azide	Ultrasonication for 60 s	5.0	−46 ± 7.2	~ 100	–	841 ± 13.4 nm	[158]
Canola oil	Protein isolate (WPI) gel particles	12,000 rpm, 3 min	5.0	~ 0	–	–	1–2 μm	[160]
Sunflower oil	CMC, lecithin	11,500 rpm for 20 min	–	–	–	–	392.5 ± 11.5 nm	[161]
Lecithin Ethanol	Debranched starch, Tween	10,000 rpm for 5 min	5.0	−17.01 ± 0.02	71.11	12.07	170.87 ± 4.70 nm	[166]
Corn oil	Pea protein nanoparticles, hydrolyzed rice glutelin fibrils, gluconic acid d-lactone, sodium azide	18,000 rpm for 3 min	7.0	–	–	–	1.02—1.42 μm	[167]
Triolein	Chitosan, *κ*-carrageenan and CMC, acetic acid, Tween 80, NaCl	12,000 rpm for 5 min	–	–	83.9–99.6	–	50–500 nm	[172]
Coconut oil	Carboxymethyl chitosan, polyglutamic acid	13,000 rpm for 3 min	–	–	82.3–91.3	–	24.8–32.2 μm	[173]
Canola oil	Colloidal silica solution, sodium azide	8000 rpm for 2 min	6.5	~ −60	–	–	0.44 μm	[174]
Soybean oil	Quaternized alkali lignin	11,000 rpm for 2 min	7.0	~ 0	50.08	21.11	–	[175]

It is obvious that emulsion-based delivery systems represent a solution to the hydrophobicity and high biodegradability of curcumin, the molecule being protected by encapsulation in an oil phase dispersed in water. Their effectiveness derives from the ability to significantly increase the apparent solubility and enhance the bioaccessibility (with reported values reaching about 60%). However, the stability of these systems is, by definition, kinetic, not thermodynamic, which implies a constant risk of coalescence and cremation (phase separation), a risk amplified in the case of O/W/O formulations. A solution to this instability lies in the development of nanoemulsions (particles below 200 nm) and, in particular, Pickering emulsions, which use solid nanoparticles for irreversible mechanical stabilization at the oil–water interface. The performance is influenced by the nature of the oil phase, where short-chain triglycerides offer superior protection against oxidative degradation compared with unsaturated oils. In addition, advanced formulations, such as multilayer cryogels, allow for controlled modulation of curcumin release, often based on pH variations, specifically targeting the gut. Although the use of emulsions is efficient and versatile, success depends on rigorous multifactorial optimization, including precise control of the zeta potential and selection of polyelectrolytes to ensure robust physical stability and targeted release kinetics.

Compared with microcapsule-based techniques such as spray drying, extrusion, or ionotropic gelation, emulsion-based systems generally provide faster release and higher initial bioaccessibility, but less long-term protection during storage. For this reason, emulsions are frequently coupled with secondary encapsulation strategies, such as spray drying or gelation, to produce hybrid systems that combine the advantages of liquid-state solubilization with solid-state stability.

At the molecular level, the performance of emulsion-based curcumin delivery systems is governed by the interactions between curcumin, lipid components, and interfacial stabilizers. Hydrophobic interactions, hydrogen bonding, and steric effects influence the localization of curcumin within the oil phase and at the oil–water interface, thereby affecting the stability and release. Molecular docking and molecular dynamics simulations can provide valuable insights into these interactions, supporting the rational selection of oils, emulsifiers, and interfacial modifiers to optimize the retention and bioaccessibility of curcumin.

Emulsion-based delivery systems are thus a flexible and effective strategy for curcumin encapsulation, particularly in applications where rapid dissolution and enhanced intestinal absorption are desired. Their integration with solid encapsulation techniques and predictive in silico tools offers promising opportunities for the development of advanced hybrid formulations with improved stability, bioavailability, and application-specific performance.

### Encapsulation in Cyclodextrins

**Fig. 15:**
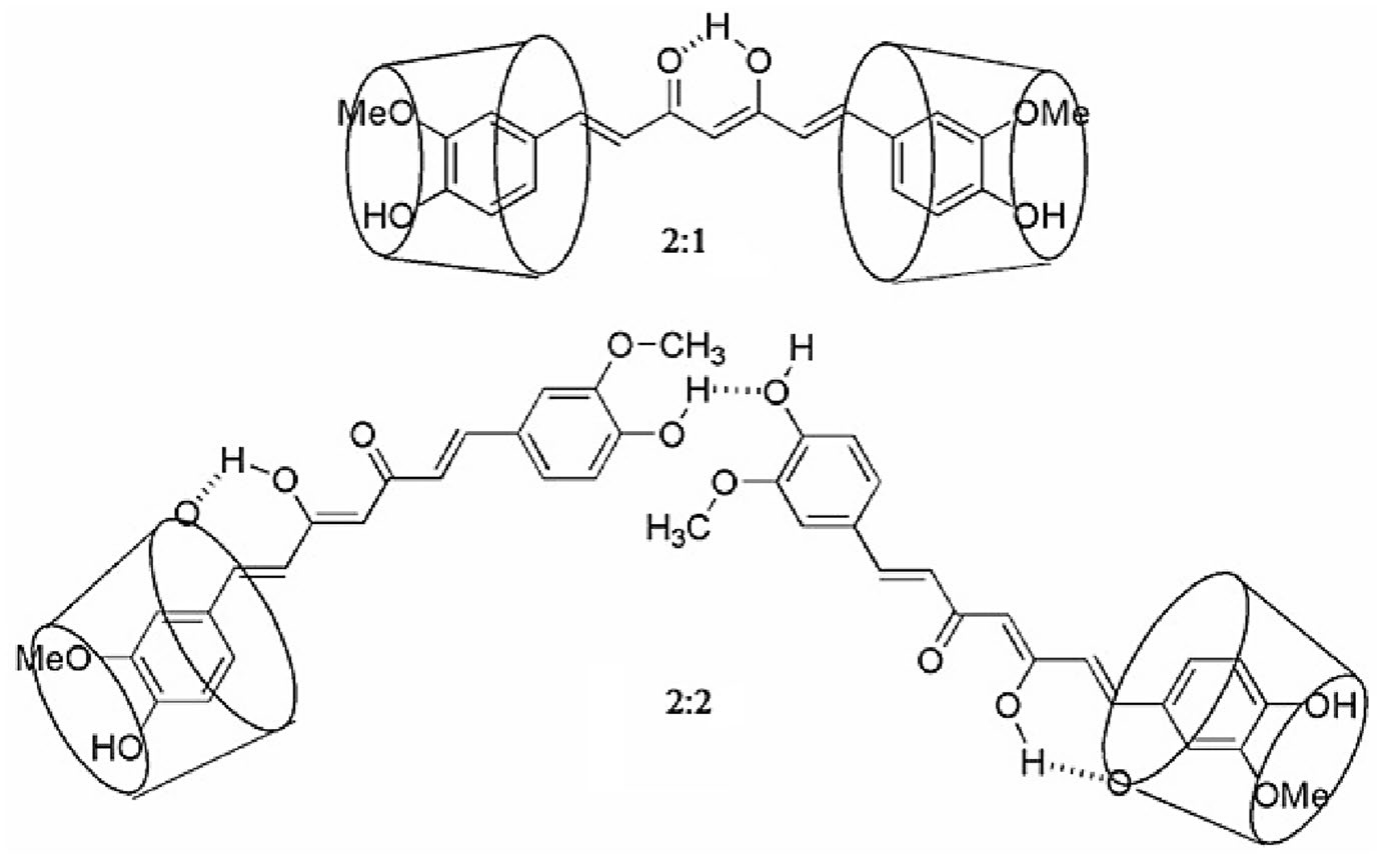
Curcumin–cyclodextrin complexes [12]

Complementary to emulsion-based systems, which enhance the solubility of curcumin through dispersion within the lipid phase, a distinct molecular-scale approach is complexation with cyclodextrins (CDs), which achieves solubilization via molecular inclusion interactions without requiring the formation of a particulate matrix. Cyclodextrins are natural, biodegradable, and biocompatible oligosaccharides, having a cylindrical structure with a hydrophobic interior and a hydrophilic exterior, which allows them to form inclusion complexes with hydrophobic molecules, such as curcumin, thus increasing its solubility in water and chemical stability. Formally, being a supramolecular reaction (without covalent bonds), this approach belongs to the field of host–guest chemistry. The complex formed between cyclodextrin and curcumin is based on molecular recognition and involves hydrophobic interactions, hydrogen bonds, and/or van der Waals forces, which ensure the stability of the complex. Research has proposed models with varying stoichiometries (2:1 and 2:2) and explored complex receptors (hosts), such as those in which two γ-CD units are covalently linked by bridges (–NH(CO)–CH_2_-CH_2_(CO)NH–), allowing study of the excited state of the contained curcumin (Fig. 15).

Cyclodextrins can be used in their natural monomeric form (β- and γ-CD), or as chemically modified derivatives (e.g., hydroxypropyl-β-CD), with physicochemical properties that can facilitate encapsulation [176]. Important parameters that determine the improvement of curcumin solubility and the effectiveness of cyclodextrins for encapsulation include the type of cyclodextrin used, the stoichiometric ratio between the host molecule and the guest molecule, and the method of preparation of the complex.

For example, β-cyclodextrin (β-CD) has been widely used for curcumin encapsulation by employing both coprecipitation and simple mixing methods [177]. Among these, simple mixing proved more practical owing to its ease of operation and lower resource requirements. Complexing curcumin with β-CD increased its water solubility by approximately 20-fold and improved its stability under unfavorable conditions, such as high temperatures and light exposure. In contrast, nanoprecipitation using ethanol as a crosslinking agent significantly enhanced the solubility, by over 200-fold compared with pure curcumin, by promoting rapid self-assembly of nanoparticles at the ethanol–water interface. However, this method produced relatively large particles (~ 200 nm) and slightly lower encapsulation efficiency compared with the simple mixing method [178].

An alternative approach involves adsorbing curcumin onto preformed nanoparticles cross-linked with tetrafluoroterephthalonitrile. This strategy provides superior long-term stability under light exposure, robust physical resistance, and rapid, selective inhibition of cancer cell growth, with HeLa cells being particularly sensitive [179].

γ-Cyclodextrin (γ-CD) offers a larger inclusion cavity (427 Å^3^ compared with 262 Å^3^ for β-CD) and higher water solubility, making it particularly suitable for food and pharmaceutical applications [180]. Conjugation with proteins such as lysozyme or bovine serum albumin enhances the nanoparticle stability, reduces cyclodextrin aggregation, and better protects curcumin [181, 182]. Further chemical modifications, for example, with succinic acid, allow ionic self-assembly with polymers such as chitosan, resulting in nanoparticles capable of controlled curcumin release in the small intestine [183].

Metal–organic frameworks (MOFs) provide a porous crystalline network that can accommodate curcumin and, when integrated with cyclodextrins, ensure biocompatibility and safety for nutraceutical applications. For instance, ultrasound-assisted solvothermal synthesis of γ-CD-based MOFs allowed precise nanoparticle size control and curcumin encapsulation, increasing the solubility by nearly 2600-fold and maintaining antioxidant stability under UV exposure [184, 185]. Further functionalization with l-glutamine enables tumor-specific targeting, achieving high loading capacity and encapsulation efficiency, while gelatin coatings provide pH-sensitive, controlled release in the acidic tumor microenvironment [186].

While β-CD complexes offer an effective and straightforward enhancement of curcumin solubility and stability, more advanced strategies, such as γ-CD-protein conjugates or CD-based MOFs, provide superior functional control, targeted delivery, and long-term protection, albeit with greater preparation complexity.

To highlight the main characteristics of curcumin–cyclodextrin complexes, Table 8 summarizes relevant data reported in the literature on the properties of these systems.

**Table 8:** Comparison of various CD and derivative-based curcumin microencapsulation systems, highlighting preparation methods and compound delivery efficiency

Cyclodextrin modification/functionalization	Curcumin–cyclodextrin complex preparation method	Loading capacity/loading efficiency (%)	Encapsulation efficiency (%)	Particle size	Yield (%)	Ref.
β-Cyclodextrin	Coprecipitation at different times of magnetic stirring, simple mixing	14%	72 95	–	72 97	[177]
β-Cyclodextrin	Dissolution in 99.7% anhydrous ethanol	–	23.97	–	–	[178]
β-Cyclodextrin was covalently crosslinked with tetrafluoroterephthalonitrile	Curcumin was absorbed into the crosslinked cyclodextrin nanoparticles	43.2 wt%	85–90	20–30 nm	> 60	[179]
g-Cyclodextrin was grafted with lysozyme using epichlorohydrin as the cross-linking agent	Mixing and ultrasound treatment	12.52%	76.74	206.30 nm	–	[182]
β-Cyclodextrin was chemically modified with succinic acid and chitosan	Ionic self-assembly	0.36 mg/g	–	500 nm	–	[183]
γ-Cyclodextrin-metal–organic frameworks	Simultaneous dissolution of crystalline γ-CD-MOFs and curcumin in methanol	30.97 ± 0.71%	67.31 ± 2.25	500–900 nm	82.50 ± 3.47%	[184]
g-Cyclodextrin was used to form a metal–organic framework with K ^+^ ions	Solvothermal method	30.97 ± 0.71 (for 2 mg/mL) turmeric	82.82 ± 3.09 (for 1 mg/mL) turmeric	~ 235.43 nm	–	[185]
β-Cyclodextrin metal–organic frameworks with gelatin and glutamine	Mixing, evaporation, and drying	55.63%	83.45	293 ± 5 to 301 ± 7 nm	–	[186]
Succinic acid-modified cyclodextrin (SACD)	Host–guest interactions between SACD and curcumin	10 mg/g	> 80	–	–	[187]

Although the encapsulation of curcumin in cyclodextrins represents a highly promising strategy for increasing its solubility, stability, and even bioavailability, the choice of the cyclodextrin type and preparation method remain essential factors that need to be adjusted depending on the intended application and the characteristics of the host and guest molecules. It is remarkable that, by chemical modification and functionalization of CDs, the systems can be “designed” for specific targeting, controlled release, and even adaptation to the tumor microenvironment, thus enhancing the therapeutic potential of curcumin.

However, there are also clear limitations: not all encapsulation methods lead to high efficiencies and optimal particle sizes for pharmaceutical or food applications. For example, the nanoprecipitation method is attractive owing to its speed and high degree of solubilization, but it can generate larger particles and reduced encapsulation efficiency. Also, some types of natural cyclodextrins have limited solubility in water and may require additional derivatization, which involves additional costs and chemical steps.

In the long term, the key feature of this technique lies in its versatility, being able to integrate other functionalities (including metal–organic frameworks, MOFs) for advanced biomedical applications and smart curcumin release. However, for industrial-scale transfer and clinical use, it is necessary to balance the parameters of efficiency, safety, and cost, as well as develop rigorous protocols to evaluate the real bioaccessibility and bioavailability in relevant biological models.

Compared with other encapsulation strategies discussed in this section, cyclodextrins offer a relatively simple and well-defined molecular system, with the advantage of precise host–guest interactions. However, cyclodextrin-based systems may provide less long-term protection than polymeric or inorganic matrices and often require relatively high cyclodextrin-to-curcumin ratios to achieve effective encapsulation. In addition, the stability of inclusion complexes can be influenced by competing hydrophobic compounds, which may limit the performance in complex formulations. Cyclodextrins are frequently combined with other delivery systems, such as emulsions, nanoparticles, or polymeric matrices, to create hybrid formulations that leverage the solubilization capacity of cyclodextrins alongside the structural stability of larger carriers. Such combinations can enhance the encapsulation efficiency, improve stability during storage, and provide additional control over release and bioaccessibility.

At the molecular level, the formation and stability of cyclodextrin–curcumin inclusion complexes depend on the geometric compatibility, binding affinity, and dynamic behavior within the host cavity. Molecular docking and molecular dynamics simulations are particularly well suited to investigate these interactions, enabling the prediction of binding modes, orientation, and stability of curcumin within different types of cyclodextrin. These in silico tools can thus support the rational selection of cyclodextrin derivatives and guide the design of optimized inclusion complexes.

Overall, cyclodextrin-based encapsulation represents an effective molecular strategy for improving the solubility and stability of curcumin, complementing the matrix-based and particulate systems discussed in previous sections. Their integration with advanced encapsulation techniques and computational modeling approaches may provide promising opportunities for the development of multifunctional curcumin delivery systems tailored to specific food, pharmaceutical, and biomedical applications.

### Comparative Analysis of Curcumin Encapsulation Technologies with Respect to Performance, Limitations, and Translational Feasibility

Despite the wide methodological diversity reported for curcumin encapsulation, a comparative analysis reveals major differences among technologies, not only at the level of underlying principles, but, more importantly, in the balance between stability, release control, bioavailability enhancement, and feasibility for industrial-scale application. For this reason, these technologies must be evaluated relatively, in direct comparison with one another rather than in isolation. From an industrial feasibility perspective, conventional spray drying remains superior to most mild encapsulation methods owing to its operational robustness and ease of scale-up. However, this advantage is achieved at the cost of reduced protection of curcumin compared with ionotropic gelation or coacervation, particularly at elevated temperatures and within porous matrices. Nevertheless, when formulated as powders, spray-dried systems often provide stability that is comparable to or even superior to that of liquid emulsions.

Regarding chemical and thermal protection, layered double hydroxide (LDH)-based systems and well-stabilized coacervates outperform classical spray drying and hot-melt extrusion, owing to their effective structural barrier against thermal, photochemical, and oxidative degradation. However, LDH systems are less versatile and more difficult to integrate into food-related applications, while coacervation is more sensitive to variations in pH and ionic strength than ionotropic gelation. Release control is generally superior in ionotropic gelation and complex coacervation systems compared with spray drying and conventional emulsions, particularly through pH-responsive mechanisms or matrix degradation. Ionotropic gelation offers more predictable and reproducible release profiles owing to its relatively simple electrostatic mechanism, whereas coacervation enables finer interfacial control, albeit within a narrower processing window. Emulsions, although capable of rapidly increasing bioaccessibility, are predominantly characterized by fast release kinetics and limited long-term control.

From a bioavailability standpoint, emulsions and cyclodextrin-based systems are generally more effective than spray drying and ionotropic gelation, as they act directly on the apparent solubility of curcumin. This advantage, however, is often transient, being limited by the kinetic instability of emulsions and by hydrophobic competition effects in cyclodextrin complexes. In contrast, solid systems obtained via spray drying or extrusion tend to provide more modest, but reproducible, bioavailability enhancements. Hot-melt extrusion occupies an intermediate position between mild laboratory-scale methods and industrially robust technologies, offering greater reproducibility than electrospraying and coacervation, but reduced thermal protection and formulation flexibility. Its primary advantage lies in the generation of amorphous solid dispersions, which may outperform spray-dried systems in terms of dissolution rate, albeit with a risk of recrystallization in the absence of adequate stabilization.

Overall, none of the analyzed technologies simultaneously optimizes all the performance axes: spray drying excels in industrial feasibility but is inferior in protection. Ionotropic gelation and coacervation provide superior release control but are limited in scalability. Emulsions and cyclodextrins maximize solubilization yet suffer from stability issues. LDH-based systems ensure exceptional protection at the cost of increased complexity and restricted applicability. A comparative synthesis of the main curcumin encapsulation technologies, considering stability, release behavior, bioavailability enhancement potential, and translational–industrial feasibility, is presented in Table 9.

**Table 9:** Performance, limitations, and translational potential of curcumin encapsulation technologies

Encapsulation technology	Curcumin stability (processing + storage)	Release control	Bioavailability enhancement potential	Translational feasibility (scalability, robustness)	Technical advantages	Technical limitations
Conventional spray drying	Moderate; risk of thermal and oxidative degradation, matrix-dependent	Limited; typically rapid release	Moderate; dependent on dispersibility	Very high	Industrially established process, high yield, low cost, high reproducibility, compatibility with multiple matrices	High processing temperatures, porous particles, limited microstructural control; nozzle clogging risk
Nano-/vacuum spray drying	Better than conventional spray drying; reduced thermal stress	Moderate–good	Good; submicron particle formation	Medium	Lower thermal stress, finer particles, narrower size distribution	Complex optimization, reduced yield and throughput; higher equipment complexity
Electrospraying	Very high; solvent-based, non-thermal process	Good; homogeneous particles	Good–very good	Low–medium	Mild processing conditions, narrow particle size distribution, good size control	Jet instability at high voltages (> 15 kV); high sensitivity to viscosity, tip–collector distance, and voltage; difficult scale-up
Ionotropic gelation	Very high during processing; storage stability dependent on drying	Very good (pH-dependent)	Moderate	Medium	Mild conditions, wide range of biocompatible materials, pH-responsive release	Difficulty in achieving uniform particle distribution due to colloidal aggregation; sensitivity to gastrointestinal pH
Complex coacervation	Good–very good when adequately stabilized	Very good; fine interfacial control	Moderate	Medium–low	High encapsulation efficiency, precise interfacial control	Requires strict control of pH, temperature, and concentration to maintain phase separation
Extrusion (HME/PGSS)	Moderate; controllable thermal risk	Good; sustained release via matrix	Good (amorphous dispersions)	Medium–high (especially pharmaceutical sector)	Solvent-free, robust process, industrially established (pharmaceuticals)	Thermal degradation risk, limited drug loading, recrystallization potential; relatively slow production
Layered double hydroxides (LDH)	Excellent; strong chemical and thermal protection	Good; often pH-triggered	Moderate	Low	Outstanding structural protection, high chemical and thermal stability, pH-responsive systems	Potential burst release under acidic conditions; complex synthesis and structural optimization; regulatory uncertainty
Emulsions/nanoemulsions	Low–moderate	Limited; rapid release	Very good	Medium	Rapid increase in bioaccessibility, relatively simple formulation	Risk of coalescence, creaming, lipid oxidation
Pickering emulsions	Better than conventional emulsions	Moderate	Very good	Medium	Reduced need for surfactants, improved physical stability	Complex interfacial control, long-term stability challenges
Cyclodextrins	Good (molecular complexation)	Limited; host–guest equilibrium	Very good (solubilization-driven)	High	Efficient solubilization, biocompatible materials, simple formulation	High CD:curcumin ratios, hydrophobic competition; higher cost compared with simple polysaccharides
Hybrid systems (e.g., emulsion + solidification, CD + spray drying)	Very good	Good–very good	Good–very good	Medium–high	Combination of complementary advantages; high formulation flexibility	Increased formulation complexity; multifactorial optimization required

This comparative analysis supports the notion that hybrid strategies represent the most promising direction for development, as they allow the combination of complementary advantages offered by multiple technologies. For example, integrating nanoemulsification or cyclodextrin complexation with a subsequent spray-drying or gelation step can transform a system with high bioavailability but limited stability into a solid, stable, and scalable product. Consequently, the selection of an encapsulation technology should not be regarded as a choice between competing methods, but rather as a multicriteria optimization process, dependent on the intended application and translational–industrial constraints.

## Molecular Docking and Molecular Dynamics Simulations of Curcumin Encapsulation

### Rationale for In silico Approaches in Curcumin Delivery Research

The design of effective curcumin delivery systems is intrinsically challenging owing to the compound’s structural flexibility, hydrophobicity, and chemical instability. Experimental formulation approaches, while indispensable, are often based on empirical optimization and trial-and-error processes that can be time-consuming and resource-intensive. In this context, in silico modeling tools, particularly molecular docking and molecular dynamics (MD) simulations, have emerged as powerful complementary approaches for elucidating curcumin–carrier interactions at the molecular level and guiding rational formulation design.

Curcumin microencapsulation techniques have improved the stability, solubility, and controlled release of this bioactive compound, thus facilitating its use in food, nutraceutical, or pharmaceutical formulations. Curcumin has demonstrated anticancer properties by modulating several molecular pathways involved in tumor cell proliferation and survival. By inhibiting pro-inflammatory transcription factors such as NF-κB and activator protein-1 (AP-1), as well as protein kinases that regulate the cell cycle, curcumin induces apoptosis and reduces cell invasion [188–190].

To further understand how encapsulated curcumin molecularly interacts with biological targets, molecular docking methods and molecular dynamics simulations provide a complementary perspective. These computational methods allow the analysis of specific interactions between microencapsulated curcumin and target proteins, thus contributing to the elucidation of molecular mechanisms and the optimization of the therapeutic potential of the compound. Therefore, molecular docking and molecular dynamics simulations performed on this bioactive compound from turmeric represent a computational approach to understand how this compound interacts with various target proteins to evaluate the potential of curcumin as a drug or therapeutic molecule [191, 192].

These computational methods play an essential role in understanding the molecular interactions, the system stability, and how curcumin is retained in the carrier matrix (such as micelles, nanoparticles, or liposomes). Furthermore, molecular dynamics simulations allow the observation of curcumin–carrier material (e.g. lipids, polymers) interactions, the stability of the encapsulation over time, and the dynamics of curcumin release. From various studies in the literature, it can be observed that the results of molecular dynamics simulations can be successfully integrated with experimental validation, thus confirming the predictive capacity of these models in encapsulation performance [193]. Moreover, molecular simulations methods combined with various experimental data (Fourier-transform infrared (FTIR) analysis, DSC, UV–Vis, isothermal titration calorimetry, fluorescence spectroscopy, etc.), allow a complete understanding of the encapsulation process and facilitate the optimization of nanocarriers.

### Molecular Docking: Predicting Curcumin–Carrier Interactions

Most of the research on the encapsulation mechanism of curcumin in different polymeric nanoparticles (poly(lactic-*co*-glycolic acid) (PLGA) or chitosan), cyclodextrins, liposomes, micelles, proteins (albumin), or nanoemulsions has focused on studying the interaction and structural changes that occur during the complexation between curcumin and these compounds. To investigate in detail these interactions and structural changes in curcumin based-encapsulation systems, most often the experimental results are correlated with those obtained from molecular docking. Molecular docking is a complex computational method used to predict how a small molecule (ligand) binds to a macromolecule (receptor), such as proteins or nucleic acids. The main goal of this method is to determine the optimal binding position and the energy associated with this interaction, thus suggesting the potential biological activity of the ligand [194]. The computational tools that are most widely used to perform molecular docking simulations of curcumin carriers or curcumin based-encapsulation systems are AutoDock Tools, AutoDock Vina, Biovia Discovery Studio, or Molecular Operating Environment (MOE). Two main steps are required in performing the docking process, namely sampling the ligand and using a scoring function. From this multitude of computational tools, AutoDock Vina (an open-source docking program) is the most used software, owing to its appropriate scoring function for docking simulations. AutoDock Vina uses an empirical scoring function to provide repeated analysis of linear fits of a prepared set of complex structures using protein–ligand complexes with known binding affinities, which include functional groups and a specific type of interaction [194, 195]. Lately, MOE software has proven to be the most suitable molecular docking software to study the molecular interactions and conformational changes of new encapsulation formulations of curcumin. This software uses a variety of scoring functions, such as empirical functions, force-field, or more detailed empirical functions.

The interaction of curcumin with various target protein compounds, such as a receptor (cyclin-dependent kinase 2 (CDK2)) [196] or an enzyme (cyclooxygenase (COX) and lipoxygenase (LOX)) [197] was investigated through molecular docking simulations. Thus, it was observed that curcumin revealed efficient binding due to the hydrophobic and polar interactions. These molecular docking results were supported by molecular dynamics results, which indicated a high stability of the curcumin–protein complexes, even if a series of minor but acceptable deviations were recorded.

Table 10 summarizes some key molecular docking simulations of curcumin carriers or curcumin-based encapsulation systems, specifying what insights were obtained through these simulations. At the same time, the close correlation of the experimental data analyzed with different experimental techniques (such as, UV–VIS spectroscopy, steady-state fluorescence, time-resolved fluorescence, and circular dichroism spectroscopy) with those obtained from molecular docking simulations is highlighted.

**Table 10:** Molecular docking simulations of curcumin carriers or curcumin-based encapsulation systems

Encapsulation system/carrier	Docking software	Key binding interactions	Binding affinity/binding energy	Correlation of molecular docking data with curcumin encapsulation	Ref.
Curcumin–β-cyclodextrin complex inclusion	Biovia Discovery Studio (DS) 2020	More favorable binding interactions were identified in the 1:1 complex due to the presence of hydrogen bonds	Binding energies: −19.5 to −16.98 kcal/mol (1:2 complex ratio); −23.5 to −20.2 kcal/mol (1:1 complex ratio)	Molecular docking simulations showed that 1:1 inclusion complex can provide a stronger binding than 1:2 complex, thus ensuring favorable encapsulation	[198]
Pea protein isolate–curcumin (PPI–Cur) nanoparticles	Discovery Studio 2016	Legumin, one of the main components of pea protein isolate, interacted with curcumin through eight hydrogen bonds and some hydrophobic interactions	CDOCKER interaction energy: 41.9489 kcal/mol (legumin–curcumin)	Molecular docking simulations of PPI-Cur complex revealed that the binding of curcumin to pea protein isolate was mainly achieved through hydrogen bonds and hydrophobic interactions. According to these simulations data, pea protein isolate can be considered a suitable encapsulation material for hydrophobic compounds	[199]
Chitosan (CS)–curcumin nanoparticles	AutoDock Vina	Strong CS–curcumin interactions due to formation of hydrogen bonds	Binding affinity: −4.3 kcal/mol	Molecular docking simulations predicted a proper CS–curcumin interaction, showing new insights into the stability and release of curcumin from nanoparticles	[200]
Curcumin–β-cyclodextrin (Cur–β-CD) inclusion complex	AutoDock Vina	Formation of multiple suitable non-covalent interactions as consequence of hydrogen bonds presence	Binding affinity: −6.3 kcal/mol (Cur-βCD)	Molecular docking simulations of human angiogenin (hAng) with the inclusion complex of Cur–βCD showed an appropriate affinity for the ribonucleolytic as well as to the nuclear translocation site. This interaction ensures a change following encapsulation within the β-cyclodextrin (βCD) cavity	[201]
Curcumin-loaded chitosan–PVA hydrogels	Molecular Operating Environment (MOE)	Curcumin formed three hydrogen bonds with specific residues from protein target casein kinase-1 (CK1); meanwhile in the case of the protein target glycogen synthase kinase-3β (GSK3B) only one hydrogen bond was identified	Binding energies: −6.9875 kcal/mol (with CK1); −6.5164 kcal/mol (with (GSK3B)	Molecular docking simulations of curcumin with the two target proteins (CK1 and GSK3B) pointed to the fact that the formulations retain curcumin binding to targets. So, the wound healing (WH) activity of the developed curcumin-based hydrogel system can be demonstrated	[202]
Curcumin nanoparticles loaded with carboxymethyl cellulose (CMC-nano-CUR)	Molecular Operating Environment (MOE)	CMC–nano-CUR forms more than five hydrogen bonds with the residues of α-amylase	Binding energies: −9.97210789 kcal/mol (α-amylase enzyme); −8.07682037 kcal/mol (α-glucosidase)	Molecular docking simulations showed that curcumin-encapsulated loaded on carboxymethyl cellulose can be used as α-amylase and α-glucosidase inhibitors for diabetes mellitus treatment	[203]
β-Lactoglobulin–chlorogenic acid–curcumin (LG–CA–Cur) complexes	AutoDock Tools (version 1.5.6, Scripps Institute, La Jolla, CA, USA)	Cur forms hydrogen bonds with the amino acid residues of both α-amylase and α-glucosidase, thus ensuring the stability of the formed complex	Binding affinities: −7.9 kcal/mol (α-amylase); −6.1 kcal/mol (α-glucosidase)	Molecular docking simulations revealed that the LG–CA–Cur complex significantly inhibited the enzymatic activity of α-amylase and α-glucosidase. These findings indicated the potential of LG-CA complexes as an efficient carrier for Cur delivery	[204]
β-Cyclodextrin–curcumin (β-CD–CUR) and DNA–curcumin (DNA–CUR) complex	Biovia Discovery Studio 2021	A stable β-CD–Cur complex was formed owing to the interaction of curcumin with only one side of two β-cyclodextrins via hydrogen bonds, while the DNA–Cur complex was characterized by an increased stability as a result of the formation of carbon hydrogen bonds	CDOCKER interaction energy: 37,327 kcal/mol (DNA-CUR); 31.9449 kcal/mol (β-CD:CUR (1:1)); 27.0974 kcal/mol β-CD:CUR (2:1))	Molecular docking simulations of DNA-CUR complex have demonstrated that the interaction between curcumin and DNA seems to have the best result in terms of stability and strength of interaction. On the basis of these simulation data, the therapeutic value of curcumin can be developed in a wide range of applications, such as the elimination of peptide nanofibers or fluorescent probes	[205]
Alpha zein–curcumin complex	AutoDock Vina	Curcumin formed four hydrogen bonds with alpha zein, thus obtaining a stable complex	Binding affinity: −7.7 kcal/mol	According to this molecular docking simulation, the interaction and structural changes that occurred during complexation between zein and curcumin are key points in the curcumin encapsulation process providing an improvement in the colloidal stability of the encapsulated system	[206]
SPI–curcumin (SPI–Cur) nanocomposites	Discovery Studio v2019	A stable complex was formed due to multiple hydrogen bonds as a result of the interaction between the hydrogen bond acceptors (O−) of curcumin in alkaline medium with the donor groups (N–H) of amino acids of the two proteins (β-conglycinin (7S) and soybean glycinin (11S))	CDOCKER interaction energy for 7S: 35.88 kcal/mol at pH 3; 35.52 kcal/mol at pH 7	The interaction mechanism between curcumin and SPI at different pH-determined stages studied using molecular docking simulations, indicates that SPI-Cur can be used as a delivery system for hydrophobic neutraceuticals	[207]
*N*-acetylglucosaminyl transferase (*IcaD*)-niosomal curcumin complex	AutoDock Vina	The optimal interaction between curcumin and *IcaD* was due to hydrogen bond, hydrophobic, and van der Waals interactions	Binding affinity: −8.3 kcal/mol (*IcaD*)	According to molecular docking analysis, *N*-acetylglucosaminyl transferase (*IcaD*) is negatively regulated by niosomal curcumin and showed high binding capacity	[208]
Soybean protein isolate hydrolysates (SPIH)–curcumin nanoparticles	Discovery Studio v2019	The interaction of Cur with the two subunits of SPIH (7S and 11S) was achieved through hydrogen bonds, carbon–hydrogen bonds, and hydrophobic interactions	Binding affinity: −10.84 kcal/mol (7S–Cur complex); −10.43 kcal/mol (11S–Cur complex)	Binding affinity: −10.84 kcal/mol (7S–Cur complex); −10.43 kcal/mol (11S–Cur complex)	[209]
Globular protein ovalbumin (OVA)–Cur nanocomplexes	Discovery Studio v2019	In all simulated pH cases, hydrogen bonds and van der Waals forces were recorded between Cur and OVA	CDOCKER interaction energy: 55.64 kcal/mol at pH 10 (binding site 1); 63.75 kcal/mol at pH 11 (binding site 1) 50.99 kcal/mol at pH 12 (binding site 2)	The interaction mechanism of OVA with curcumin confirmed by molecular docking simulations performed at different pH steps, highlighted the factors affecting the effectiveness of the pH-determined method, and helped refine the application in hydrophobic delivery systems of active substances	[210]

For instance, Mashaqbeh et al. [198] studied the stability of curcumin complexation and solubilization with β-cyclodextrin and β-cyclodextrin-based nanosponges. First, the experimentally prepared complexes were characterized physicochemically (powder X-ray diffraction (PXRD), FTIR, nuclear magnetic resonance (NMR), and DSC analyses), observing the optimal formation of the inclusion complex of curcumin with the cyclodextrin polymer. To investigate in more detail the complexation mechanism of curcumin with cyclodextrin, molecular docking simulations were performed using Biovia Discovery Studio (DS) 2020. Thus, the formation of hydrogen bonds during molecular docking could be correlated with the displacement of the O–H or C=O bands in FTIR analysis. The binding energy values ​​for the complexes (1:1 complex ratio and 1:2 complex ratio) recorded from the molecular docking indicated increased stability of the curcumin–cyclodextrin complex. This was also confirmed by DSC analysis, where disappearance of the curcumin endothermal peak was observed in the DSC thermogram of the curcumin complex with cyclodextrin. However, a series of additional experimental studies are needed to demonstrate how curcumin can be encapsulated and released from prepared formulations.

For a more solid validation of the results obtained from molecular docking, it is necessary to carry out molecular dynamics studies that must be correlated with those obtained experimentally using the ultraviolet–visible spectroscopy, circular dichroism spectroscopy, or isothermal titration calorimetry (ITC) technique. For example, Ren et al. [199] prepared pea protein isolate nanoparticles using a pH-based method to encapsulate curcumin. The encapsulation propensity and interaction mechanism of pea protein isolates with curcumin and colchicine were comparatively evaluated by structural characterization, molecular dynamics simulations, and molecular docking. To evaluate the structural stability of these nanoparticles, molecular dynamics simulations at 298.15 K for 40 ns and Gibbs free energy calculations were performed. Molecular docking simulations showed that the binding of curcumin and colchicine to pea protein isolate mainly occurs through hydrogen bonds and hydrophobic interactions. These molecular simulation results were very well corroborated with the experimental ones obtained using circular dichroism spectroscopy and ultraviolet–visible spectroscopy, highlighting the existence of a complexation between pea protein isolate and curcumin or colchicine. In conclusion, the formation of a nanoparticle from curcumin and pea protein isolate was observed, characterized by a smaller particle size, a lower Gibbs free energy of binding, and a stable structure. Therefore, pea protein isolate proved to be a suitable encapsulation material for hydrophobic compounds.

**Fig. 16:**
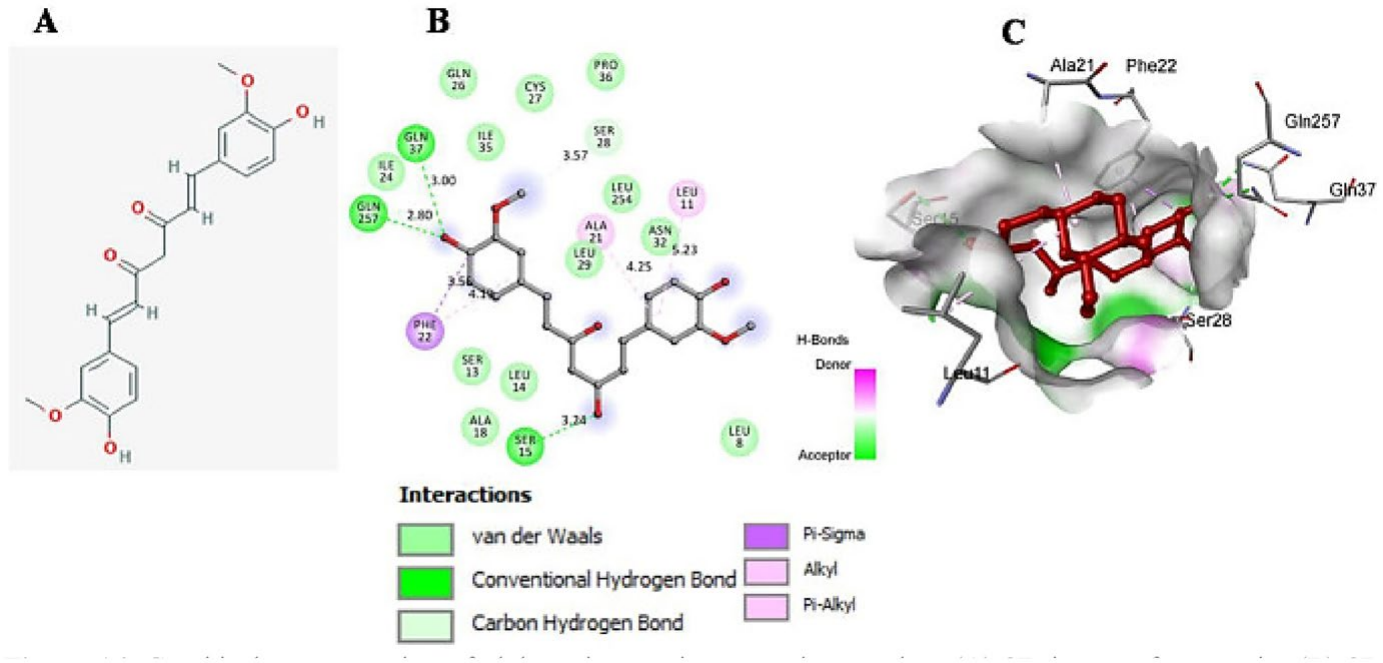
Graphical representation of alpha zein protein–curcumin complex: **A** two-dimensional (2D) image of curcumin. **B** 2D representation of interacting residues. **C** three-dimensional (3D) donor–acceptor view of binding residues [206]

The complexation process between curcumin and a series of polymers or proteins constitutes a key point in the development of formulations with the potential to encapsulate curcumin. For example, Tiwari et al. [206] studied the interaction and structural changes during complexation between zein and curcumin using spectroscopic techniques (UV–Vis, steady-state fluorescence, time-lapse fluorescence, and circular dichroism spectroscopy) and molecular docking. To obtain a good correlation with the experimental results (performed at pH 4) in molecular docking simulations, the keto form of curcumin was considered. The strong binding affinity between zein and curcumin was due to various interactions, such as van der Waals, hydrogen bonds, π-alkyl, and π-sigma, as shown in Fig. 16. All these molecular docking results are consistent with the results obtained experimentally using fluorescence spectroscopy, highlighting that the hydrophobic interaction was mainly responsible for the interaction between zein and curcumin.

**Fig. 17:**
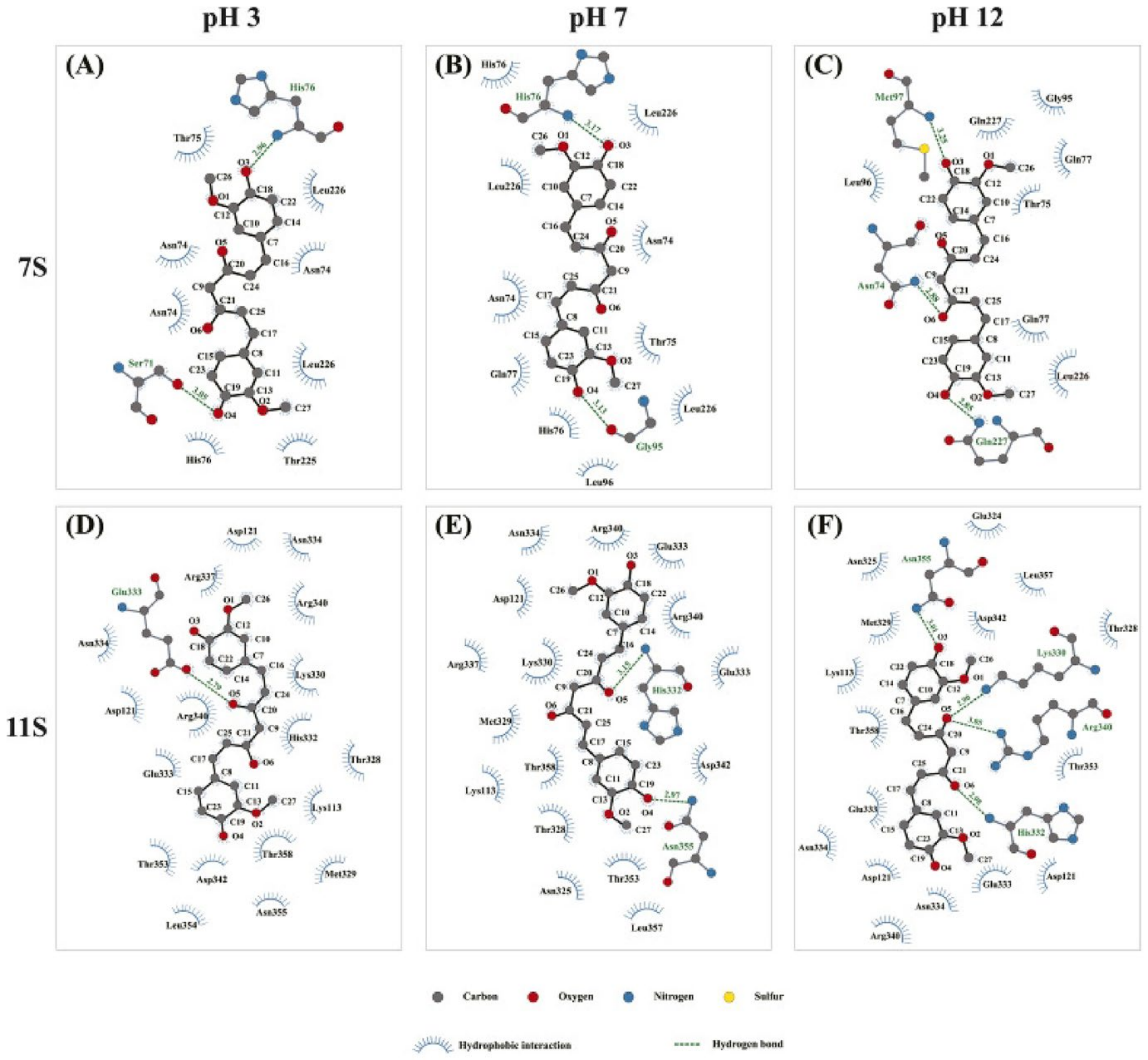
Optimal binding conformations of curcumin complexed with β-conglycinin (7S) and soybean glycinin (11S) protein molecules obtained by molecular docking. **A–C** Schematic diagram of 7S protein incubated with curcumin at pH 3, 7, and 12. **D–F** Schematic diagram of 11S protein incubated with curcumin at pH 3, 7, and 12 [207]

Meanwhile, in the case of encapsulation of curcumin by various proteins, both electrostatic interactions and pH variations play an important role in obtaining stable encapsulation formulations. Thus, Li et al. [207] investigated the fundamental mechanism of action of soybean protein isolate (SPI) with curcumin using various spectroscopic techniques (UV–Vis and fluorescence spectroscopy) and molecular docking. According to these molecular docking simulations, curcumin molecules were complexed with the two proteins (β-conglycinin (7S) and soybean glycinin (11S)) mainly through hydrogen bonds and hydrophobic interactions, which were modified at different pH-determined stages as shown in Fig. 17. The results obtained at pH 3 and 7 were close owing to the fact that curcumin was uncharged in this range. The only disadvantage of these molecular docking simulations was that it could not be demonstrated that 7S and 11S have the same affinity for curcumin at pH 3 and 7. This is due to the fact that molecular docking could not simulate the modified structure and exposure of the hydrophobic regions of the SPI molecules at different pH values. However, despite these disadvantages, the molecular docking results could be correlated with the experimental ones performed at pH 7 and 3. Curcumin reached a maximum encapsulation efficiency of 80.84% at pH 7. According to fluorescence spectroscopy experiments, hydrophobic interactions promoted the formation of nanocomposites, and curcumin had a strong fluorescence static effect on soy protein isolate.

**Fig. 18:**
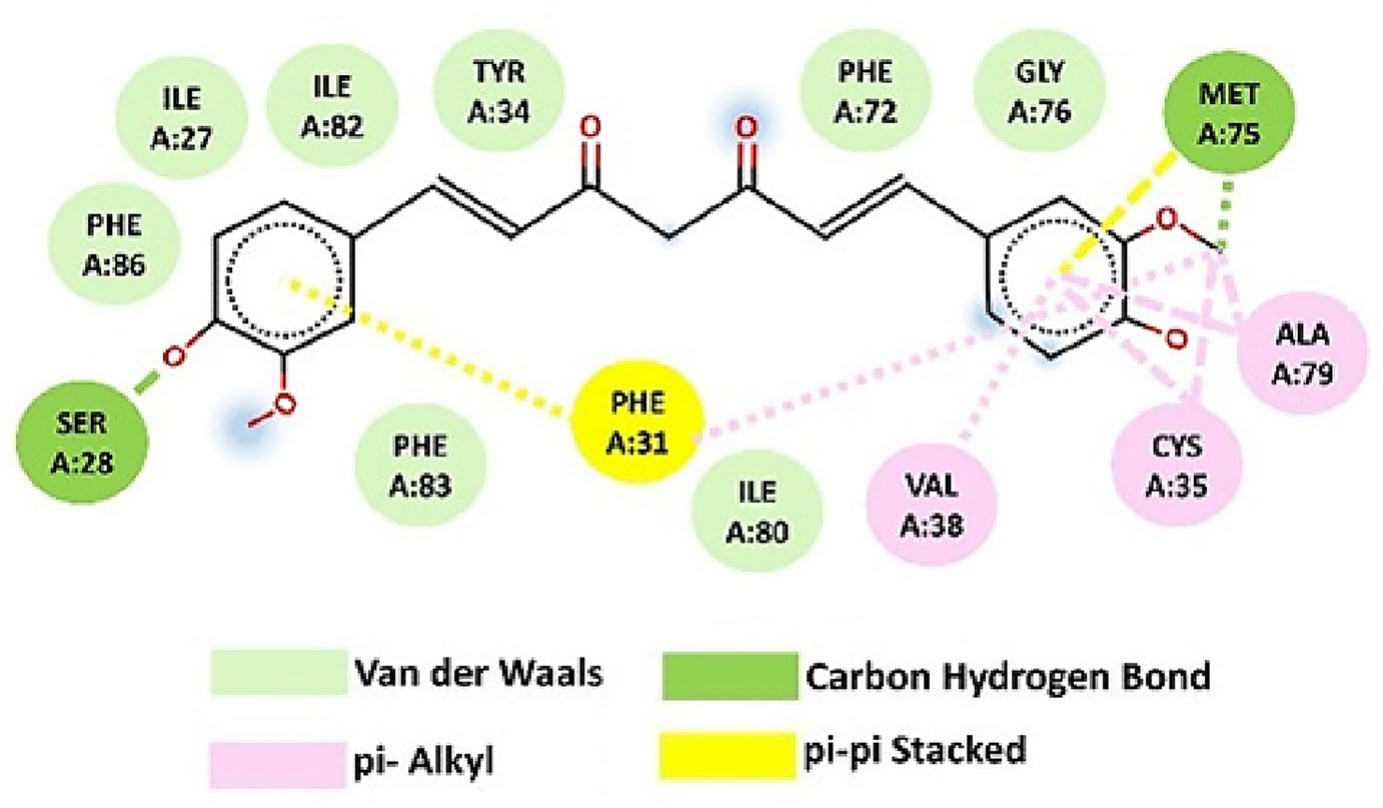
Interaction between curcumin and *N*-acetylglucosaminyl transferase (*IcaD*) [208]

Moreover, to achieve an optimal curcumin encapsulation process, it is necessary to use versatile and stable nanocarriers that can significantly increase the bioavailability and therapeutic efficacy of the encapsulated formulas. The most widely used nanocarrier in the curcumin encapsulation process is niosome. The unique bilayered structure of niosomes, formed by the self-assembly of nonionic surfactants in aqueous media, provides protection of the encapsulated compounds against premature degradation and ensures their controlled release. As such, Khaleghian et al. [208] explored the anti-biofilm action of curcumin encapsulated in niosomes by performing molecular docking simulations using Autodock Vina 1.1.2 software. Thus, in these molecular docking simulations, *N*-acetylglucosaminyl transferase (*IcaD*) was used, which is negatively regulated by niosomal curcumin. The molecular docking analysis consisted of identifying the role of *IcaD* based on its highest binding affinity (−8.3 kcal/mol) with an ideal root-mean-square difference (RMSD) of 0 and molecular interactions with curcumin (Fig. 18). These predicted interactions between curcumin and biofilm were subsequently confirmed by empirical studies examining the anti-biofilm effects of curcumin-containing niosomes and their effect on the expression of biofilm *IcaD* genes in multi-drug resistant (MDR) *Staphylococcus aureus* strains, respectively.

**Fig. 19:**
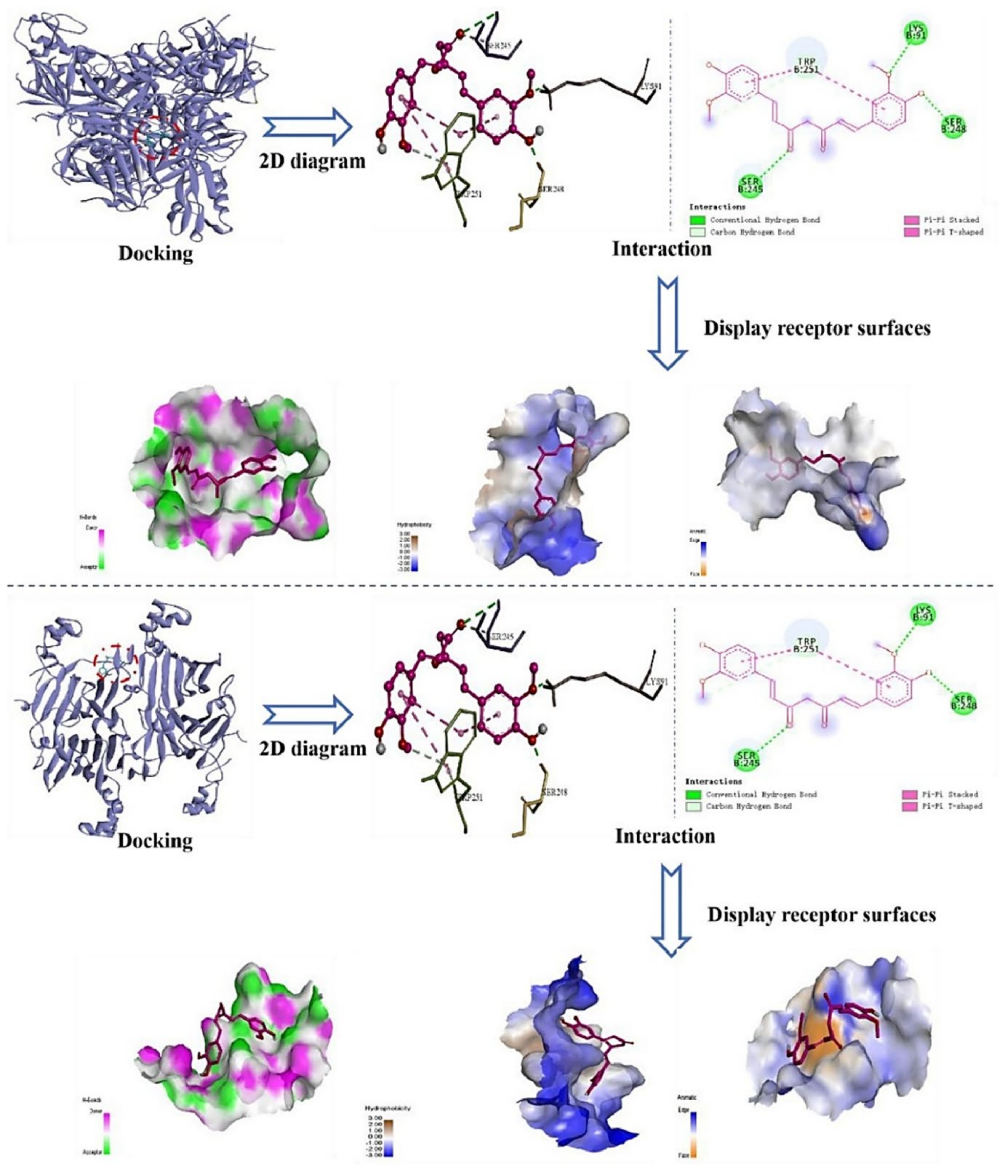
Schematic diagram of the binding process of 7S and 11S proteins to curcumin [209]

The stability, solubility, and bioavailability of curcumin in the encapsulation process can be significantly enhanced by using soy protein isolate hydrolysates (SPIH), which act as efficient, natural, and biodegradable carriers for encapsulating this bioactive compound. Thus, Ma et al. [209] analyzed the interaction modes of curcumin (Cur) with the two subunits (7S and 11S) of soybean protein isolate hydrolysates (SPIH) using molecular docking simulation. During this simulation, numerous hydrogen bonds, carbon–hydrogen bonds, and hydrophobic interactions were involved in the interaction of Cur with 7S and 11S, respectively (Fig. 19). The absolute binding energies of curcumin with SPIH exceeded 7 kcal/mol, indicating spontaneous binding of curcumin to soybean protein isolate hydrolysates. These molecular docking results were confirmed by FTIR data and fluorescence spectra, highlighting that hydrophobic interactions and hydrogen bonds are essential in the interaction between curcumin and SPIH. So, molecular docking results provide a starting point for the design of new SPIH–Cur nanoparticles owing to the increased potential of SPIH to encapsulate and stabilize lipophilic bioactive substances.

**Fig. 20:**
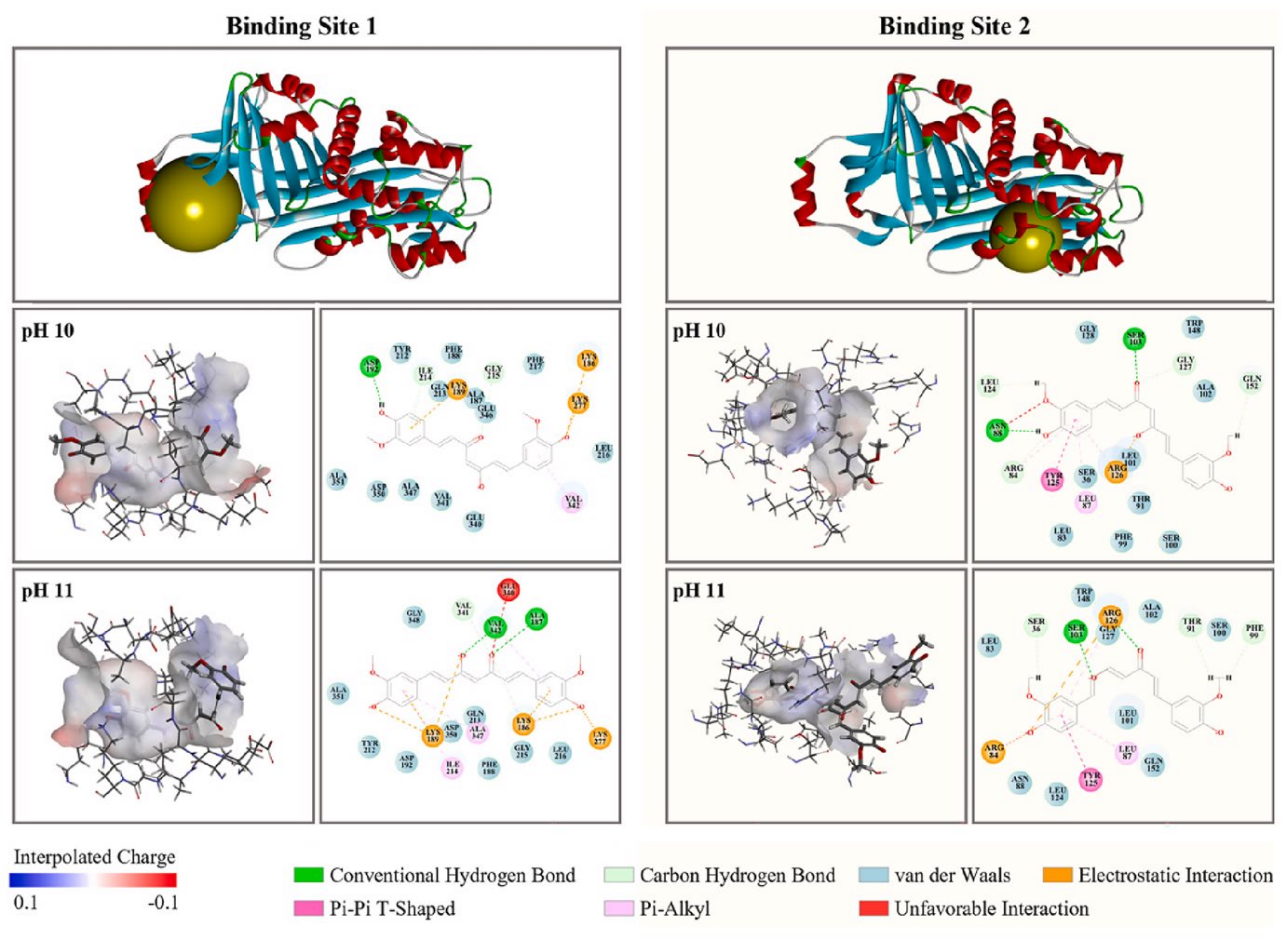
Impact of pH on molecular docking results of OVA and curcumin, including the location of binding sites, the charge state of amino acids, and 2D interaction diagrams [210]

One of the most effective methods for encapsulating curcumin has been shown to be the pH-based encapsulation technique. This method is an environmentally friendly, organic solvent-free, and effective method to encapsulate hydrophobic bioactive compounds (e.g., curcumin) in carriers such as proteins or liposomes. To simulate the pH-based encapsulation technique of curcumin with the hydrophilic globular protein ovalbumin (OVA), Li et al. [210] deprotonated the protein precisely at pH levels of 7.0, 10.0, 11.0, and 12.0. The interaction modes of this protein with curcumin were investigated using molecular docking. According to the results of this molecular simulation, two binding sites, binding site 1 and binding site 2, were identified on the basis of the receptor cavities, as shown in Fig. 20. Both binding sites produced successfully docked ligand conformations owing to the constraints related to the size of the ligand molecule and the surrounding amino acid environment. In addition, both binding site 1 and binding site 2 showed binding energy values  < −5 kcal/mol, indicating that OVA may encapsulate curcumin via a pH-driven mechanism (Fig. 20).

These molecular docking results complement the experimental results that explained the changes in the interactions between curcumin and OVA observed in fluorescence spectroscopy. Unlike these structural changes observed in fluorescence spectroscopy, those identified in the FTIR spectrum of proteins appear suddenly after a critical pH of 10, when a change in the interactions between curcumin and OVA is observed. Therefore, the interaction mechanism has shifted from being dominated by electrostatic interactions to hydrophobic interactions and a synergistic combination of both, with a transformation of the secondary structure of OVA and an increase in the curcumin solubility being observed.

### Molecular Dynamics Simulations: Assessing Stability and Release Behavior

Molecular dynamics simulations complement molecular docking studies of encapsulated curcumin in various systems, providing more detailed information about the structural behavior and stability of these formulations. Typically, molecular dynamics (MD) simulations are used to characterize the receptor–ligand interaction. The main objective of such simulations is to describe the stability, dynamics, and conformational changes of the receptor–ligand complexes over time under physiological conditions [211]. Depending on the complexity of the polymer matrix, molecular dynamics simulations can last from 1 to 100 ns, with dimensions of the polymer system that can vary from tens to several hundred nanometers. Two main software packages (GROMACS and AMBER) are used to perform molecular dynamics simulations of encapsulated curcumin in specific matrices, but each has its own particularities. Among these software packages, GROMACS ensures efficient flexibility and precision in reproducing molecular interactions [193, 212]. The most common force fields for molecular dynamics (MD) simulations of curcumin-based encapsulation systems are the Generalized Amber Force Field (GAFF), amber99SB force field, and CHARMM General Force Field (CgenFF).

Most molecular dynamics simulations of curcumin are focused on micelle or aggregate formation [213], curcumin–solvent interactions [214], and the effect of pH and temperature on the stability [215]. In recent years, some molecular dynamics simulations of curcumin encapsulation and drug delivery have been developed. Thus, various molecular dynamics simulations have been carried out with the aim of studying the binding energy and encapsulation efficiency, the release mechanisms, and structural stability of the encapsulated curcumin in certain carriers (cyclodextrins, liposomes, or polymeric nanoparticles). Therefore, molecular dynamics simulations allow the quantification of binding affinities, the identification of favorable interaction conformations, and the assessment of energetic stability, which constitutes a rational basis for the optimization of various curcumin-based encapsulation systems. Recently, several researchers have integrated computational predictions with experimental validation, demonstrating that free energy calculations based on molecular dynamics simulations can be accurately correlated with the encapsulation efficiency and functional performance. Thus, the number of trial-and-error experiments can be reduced and the efficient design of various curcumin-based delivery systems is ensured.

**Fig. 21:**
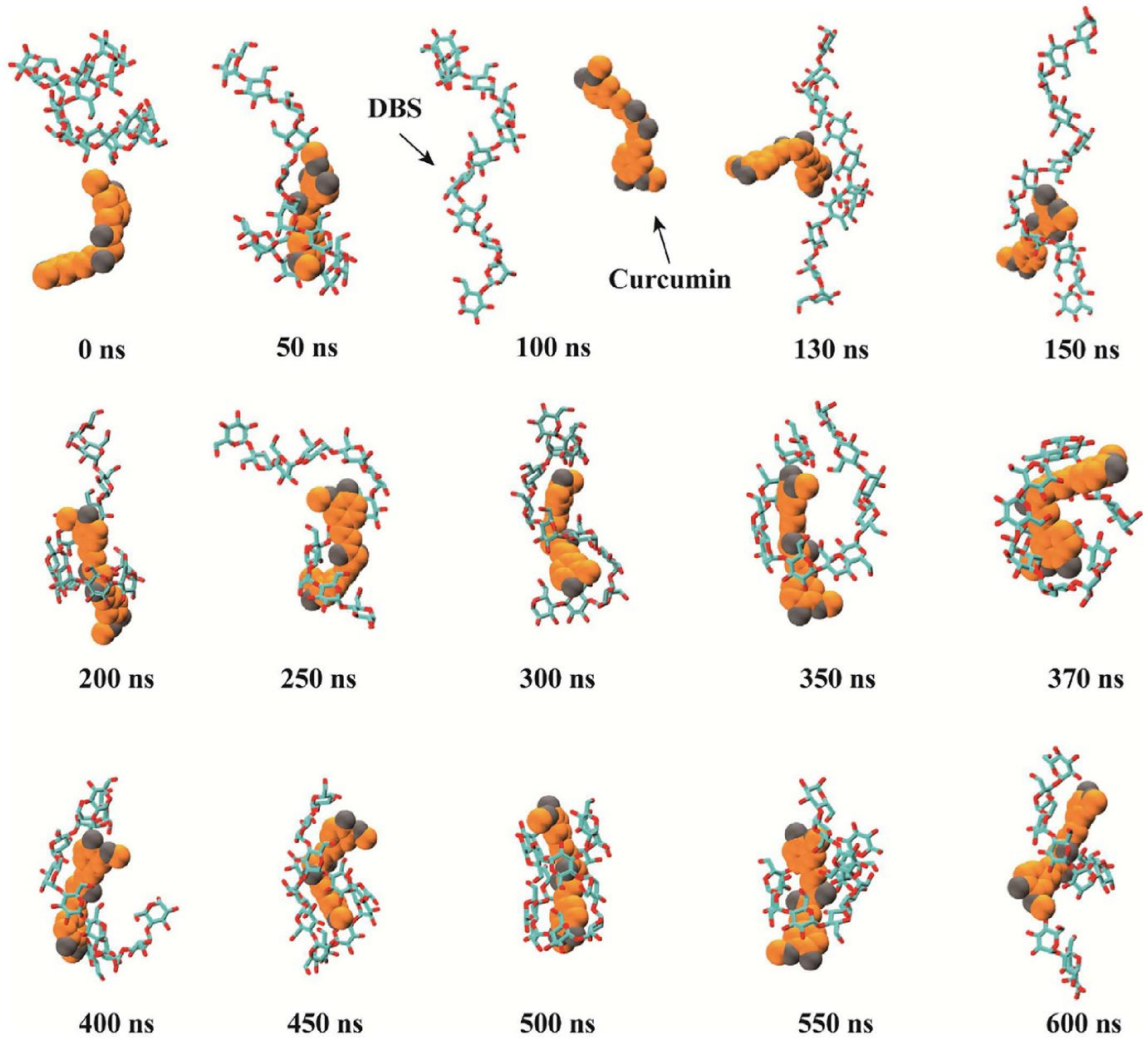
Snapshots of the conformational transitions from the initial conformation at 0 ns to a final one at 600 ns in the system of DBS in the presence of curcumin aqueous solution [166]

Feng et al. [166] analyzed the interaction mechanisms between debranched starch (DBS) and curcumin using molecular dynamics (MD) simulations performed with the AMBER 14 software suite for 600 ns, considering two force fields: GLYCAM-06 (for DBS) and GAFF (for curcumin). The difference between the two force fields lies in the purpose for which they were parameterized, in the way they treat saccharides/biomolecules, and in the accuracy of the parameters for different types of atoms/groups. GLYCAM-06 is a force field for carbohydrates, being a single set of parameters applicable to both α- and β-anomers, as well as to all sizes and conformations of monosaccharide rings. Meanwhile, GAFF is a generalist force field for small organic molecules and fragments that are not covered by other specific force fields (e.g. AMBER for proteins/DNA, GLYCAM for carbohydrates). This study consisted of analyzing the hydrogen bonds and water bridges formed between DBS and curcumin. A total of 22 stable hydrogen bonds were identified, which were formed in the system, and a hydrogen bond between water and curcumin molecules, having a proportion equal to 94.74%. All these simulation data suggest that the hydrogen bond presence was involved in the entire simulation process. Therefore, MD simulations in this study showed that the water bridge between debranched starch and curcumin may play an important role in the complexation process to obtain an emulsion-based delivery systems for curcumin, thus contributing to the better performance of this emulsion due to the increased stability and solubility of curcumin in an aqueous solution (Fig. 21). These molecular dynamics results are in agreement with the experimental results, in which the morphological examination using SEM and the thermal behavior of the particles in the emulsion by TGA analysis were followed. According to the particle morphologies resulting from SEM analysis, curcumin was encapsulated in the β-cyclodextrin cavities. This can also be supported by TGA analysis which varied the temperature from 20 to 104 °C to analyze the thermal behavior of the curcumin–DBS emulsion. Therefore, a very small weight loss was observed for curcumin, due to the loss of water, which means that this hydrophobic molecule is still present in the prepared emulsion.

**Fig. 22:**
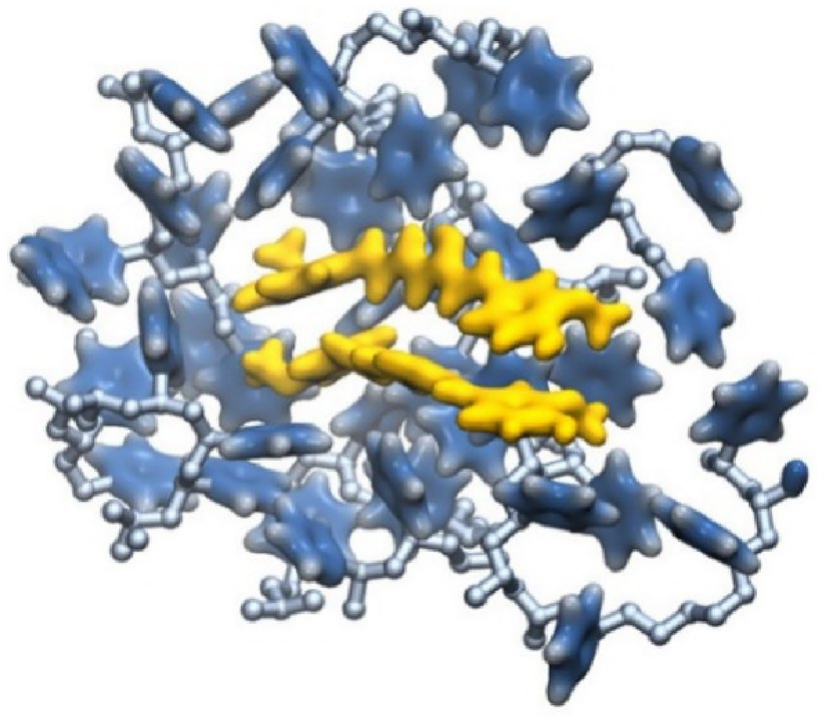
Snapshots of Cur incorporation into the PS matrix (where, Cur—Quick Surface representation in yellow and PS phenyl groups—Quick Surface representation in blue being at a distance of 1 nm from Cur) [216]

Another molecular dynamics study was conducted by Zatorska-Płachta et al. [216] to understand how curcumin molecules interact and organize within a polystyrene (PS) matrix. The main goal of these atomistic molecular dynamics simulations was to show how curcumin can be encapsulated in polystyrene-based nanoparticles. The molecular dynamics simulations were performed using GROMACS 2018 software for 500 ns to obtain a compact aggregate. MD simulations showed that the hydrophobic core of PS nanoparticles is formed by individually wrapped polymer chains, which adhere to each other. In addition, no specific interactions were identified between the PS forming core and curcumin. Therefore, according to these molecular dynamics results, it can be concluded that drugs can be incorporated into the PS matrix owing to its hydrophobicity, as shown in Fig. 22. This aggregation of curcumin observed in molecular dynamics simulations is consistent with the microscopic observations obtained using laser scanning confocal microscopy (LSCM). Indeed, LSCM images showed that, at higher drug concentrations, curcumin is distributed nonuniformly inside the microspheres.

Moreover, Rezaeisadat et al. [213] explored in detail the interaction of modified PNIPAAm-b-PEG block copolymer as a smart nanodrug delivery system with curcumin molecule as a drug, using molecular dynamics simulations. These simulations were performed using GROMACS software to investigate the behavior of the polymer in the presence of the drug in an aqueous solution. In these molecular simulations, the amber99SB force field was used to predict the polymer folding tendency, the formation of the globular structure with the decrease in the radius of gyration (*R*_g_), and the increase in interchain hydrogen bonding, respectively. In this study, a series of analyses were performed on the encapsulation behaviors of a drug, intermolecular interactions, structural properties of drug–polymer nanomicelles, and the loading capacity of the drug in the micelle. Thus, it was observed that the phase change temperature of the PNIPAAm-b-PEG polymer is in the range of 300–305 K, and at a concentration of 9% of the polymer, at 310 K, the effective radius of the formed micelle is equal to 4.36 nm, ensuring an optimal hydrogel shape.

**Fig. 23:**
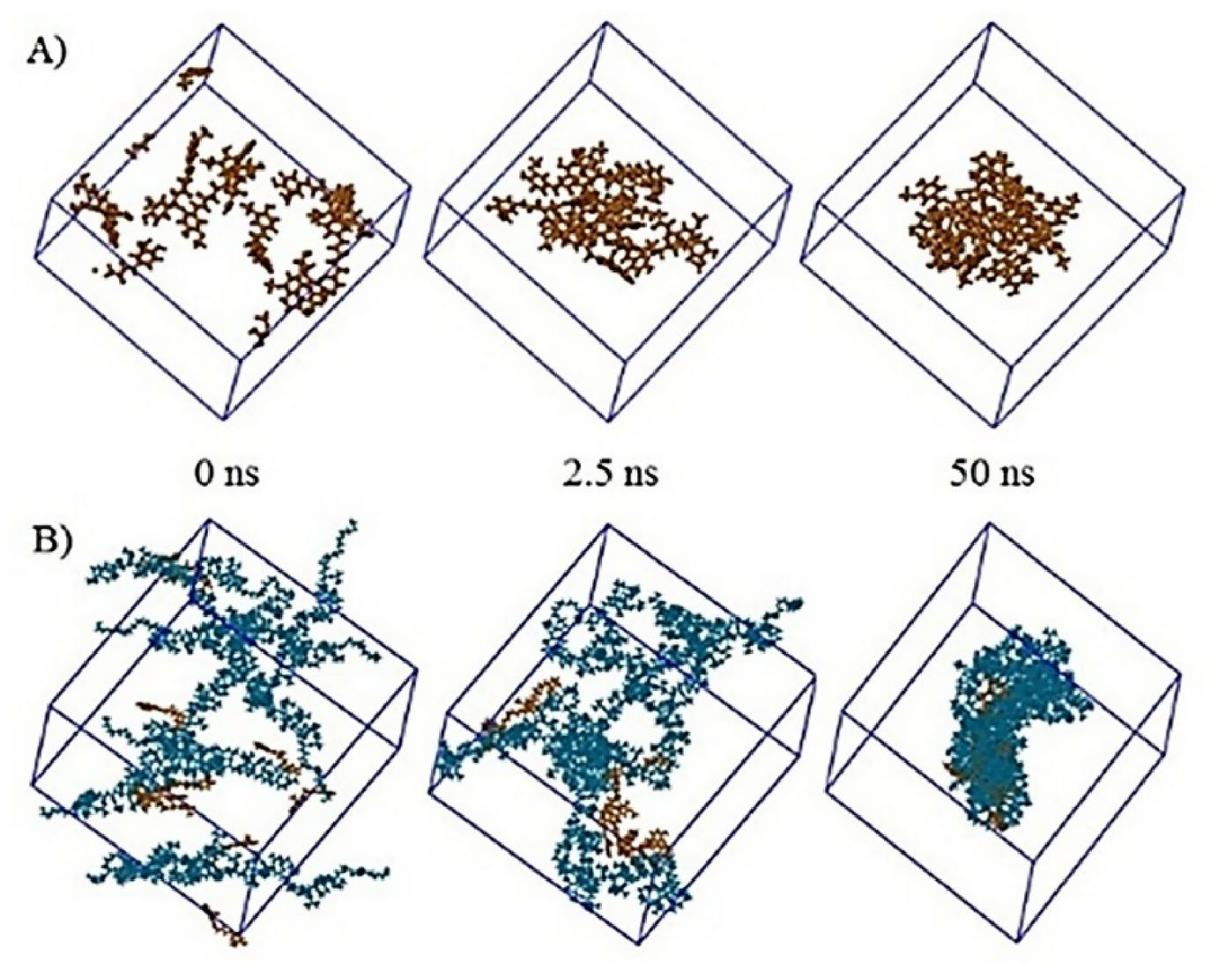
Snapshots of the **A** drug aggregation process and **B** drug encapsulation process **B** from 0 to 50 ns [213]

Furthermore, the interaction between curcumin and ten drugs that were randomly distributed in a simulation box at 310 K was simulated for 50 ns. From these simulation data, it can be seen that, in less than 2.5 ns, curcumin aggregates were formed. So, after 50 ns of molecular dynamics simulation, both the drug aggregation and encapsulation processes have fully occurred, as shown in Fig. 23.

In conclusion, MD simulations can be used to predict the properties of drug delivery systems, but their validation by experimental methods is necessary to understand in more depth the processes that cannot be observed directly. Moreover, experimental methods can examine the effects of ions and pH in the environment and the drug release by reducing the local spot temperature.

Running molecular docking coupled with molecular dynamics (MD) simulations is one of the most efficient methods to study the encapsulation of curcumin in various systems (cyclodextrins, liposomes, polymeric nanoparticles or proteins, MOFs, etc.). This combination provides complementary information that each method alone cannot provide. Molecular docking combined with molecular dynamics simulations can provide rapid prediction, realistic validation, thermodynamic analysis, and understanding of the encapsulation mechanism, thus enabling the design of more efficient systems for curcumin delivery. However, these simulations still have several limitations, such as the release rate, long-term stability, or diffusion of curcumin in the matrix. Therefore, it is recommended that these molecular simulation data be complemented with various experimental analyses.

### In Silico Modeling: Integration with Encapsulation Strategies, Limitations, and Future Perspectives

The real value of molecular docking and MD simulations lies in their integration with experimental encapsulation approaches. When applied in isolation, computational studies provide limited predictive power. However, when combined with formulation data, they enable a more rational and mechanism-driven design process.

Across the encapsulation strategies discussed in Sect. 3, in silico modeling can support:

- Carrier selection, by identifying materials with strong and stable curcumin interactions
- Formulation optimization, by predicting the impact of chemical modification or compositional changes
- Stability assessment, by evaluating curcumin protection against environmental stress
- Release prediction, by linking interaction strength to diffusion or dissociation behavior

This integrated approach aligns with the conceptual framework outlined in the “Introduction,” whereby intrinsic curcumin properties guide encapsulation strategy selection, experimental studies assess stability and bioavailability, and in silico tools provide molecular-level feedback for rational formulation optimization.

Despite their increasing utility, molecular docking and MD simulations are subject to limitations that must be acknowledged. The accuracy of docking results depends on the scoring functions and structural models, while MD simulations are constrained by force-field quality, simulation timescales, and computational cost.

The accuracy of the force field is a critical factor in molecular dynamics simulations used to investigate the encapsulation mechanism of curcumin in different matrices (micelles, cyclodextrins, polymeric nanoparticles, liposomes, etc.). The force field determines how intra- and intermolecular interactions are described, directly influencing the stability of the complex, the binding energy, and the dynamics of the encapsulation process. In many studies, curcumin parameters are obtained by semi-empirical methods or by fitting based on density functional theory (DFT) calculations, which introduces a significant dependence on the parameterization method. Nonpolarizable force fields can underestimate the effects of electronic induction, especially in heterogeneous environments, such as the hydrophobic–hydrophilic interface.

Thus, the accuracy of simulations of the curcumin encapsulation mechanism is limited by the approximations inherent in classical force fields. Validation of the results by comparison with experimental data (binding energy, complex dimensions, and thermodynamic stability) or by using hybrid quantum mechanics/molecular mechanics (QM/MM) methods is essential to increase confidence in the conclusions reached.

A major obstacle in molecular simulations of curcumin encapsulation in different systems is the sampling limitations. These limitations affect both the description of molecular mechanisms and the accuracy of thermodynamic and kinetic estimates. The use of coarse-grained MD can partially alleviate these problems but does not completely eliminate the constraints imposed by the time scales and complexity of the studied systems.

Molecular dynamics simulations performed with established software packages such as GROMACS and AMBER provide a robust framework for investigating curcumin encapsulation in supramolecular systems. However, regardless of the software implementation used, the description of the encapsulation process is limited by fundamental sampling constraints, which are only partially mitigated by the algorithms and methods available in these platforms. In all three packages, conventional atomistic MD simulations are restricted to timescales of nanoseconds to hundreds of nanoseconds, even under advanced parallelization. The processes relevant to curcumin encapsulation, including desolvation of the molecule, crossing the host cavity barrier, and structural reorganization of the system, often occur on longer time scales, leading to incomplete sampling of the free energy landscape. As a result, trajectories generated with GROMACS or AMBER tend to get stuck in local minima, and rare transitions between the free and encapsulated states are not observed spontaneously. This limitation affects the convergence of thermodynamic properties and compromises the mechanistic interpretation of the encapsulation process.

Furthermore, it remains challenging to accurately model complex building environments consisting of multicomponent systems and biological fluids.

Nevertheless, ongoing advances in computational power, force-field development, and multiscale modeling are continuously expanding the applicability of in silico approaches. The integration of docking and MD simulations with experimental formulation studies holds significant promise for reducing trial-and-error experimentation and accelerating the development of efficient curcumin delivery systems.

Molecular docking and molecular dynamics simulations have evolved as indispensable tools to understand and optimize curcumin encapsulation strategies. By providing molecular-level insight into curcumin–carrier interactions, these approaches complement experimental techniques and enable a predictive, mechanism-driven formulation paradigm. Their integration with encapsulation technologies represents an important step toward the rational design of stable, bioavailable, and application-specific curcumin delivery systems.

## Pharmacokinetic Profile and In Vivo Absorption

In the context of the formulation strategies discussed above, the evaluation of in vivo absorption and pharmacokinetic implications is essential for assessing the effectiveness of curcumin release from its dosage form. Improvements in oral bioavailability are typically supported by the analysis of key pharmacokinetic parameters, such as maximum plasma concentration (*C*_max_), half-life (*t*_1/2_), and the area under the concentration–time curve (AUC), which collectively reflect both the rate and extent of systemic exposure.

Overall, the analyzed in vivo studies highlight a wide range of pharmacokinetic approaches used to evaluate curcumin-based formulations, reflecting both the diversity of encapsulation strategies and the methodological limitations associated with each approach.

In this context, the proliposomal formulations evaluated by Adel et al. [88] demonstrate efficient encapsulation, as evidenced by increased *C*_max_ and AUC values, and a reduced *t*_max_, indicating faster and more extensive absorption compared with free curcumin powder. Moreover, prolonged pulmonary retention is reflected by the increased mean residence time (MRT) values. Similarly, Madhavi et al. [217] investigated the pharmacokinetics of Eudragit S100-based curcumin microspheres, obtained by O/O solvent evaporation, after oral administration to rats. Compared with free curcumin, the microencapsulated formulation resulted in increased systemic exposure, as evidenced by higher *C*_max_ (1.82 versus 1.02 μg/mL) and AUC (23.18 versus 7.94 μg h/mL). The delayed *t*_max_ (8 h versus 3 h), along with reduced clearance and increased *t*_1/2_, suggest slow absorption, delayed release, and prolonged systemic persistence, consistent with a colonic targeting profile.

A comparable level of pharmacokinetic robustness is observed in studies based on solid dispersions obtained by hot-melt extrusion. Both the the Eudragit^®^ EPO-based system [145] and amorphous polymeric formulation [146] resulted in substantial increases in systemic exposure, reflected by AUC values approximately 1.5–2.2-fold higher than those of free curcumin. The delayed *t*_max_ and prolonged half-life suggest a slow, but sustained absorption profile, characteristic of modified-release systems. These findings confirm that the molecular dispersion of curcumin within an amorphous matrix represents one of the most effective strategies for enhancing oral absorption, even in the absence of true nanostructuring.

On the other hand, several studies provide indirect evidence of improved bioavailability, without reporting classical pharmacokinetic parameters. For example, the shellac–locust bean gum-based CESL-NP formulation [218] was evaluated in vivo in a murine model of diabetic nephropathy and demonstrated significantly superior therapeutic efficacy at a fivefold lower dose compared with the free curcumin–EGCG combination, suggesting enhanced functional oral bioavailability. Although these findings are promising, the absence of pharmacokinetic parameter data limits mechanistic interpretation and direct comparison with other delivery systems.

A particular case is represented by lipid nanoparticles based on glyceryl monooleate [219], where extreme differences in *C*_max_ and prolonged plasma persistence following intravenous administration indicate a major contribution to protection against degradation and controlled release. However, the clinical relevance of these results for oral administration remains limited, as the route of administration bypasses the gastrointestinal barriers that are critical determinants of curcumin absorption.

Similarly, curcumin–Eudragit^®^ EPO nanoparticles obtained by one-step hot-melt extrusion [144] demonstrated a 1.68-fold increase in *C*_max_ following oral administration in rats compared with free curcumin, along with significant improvements in the *C*_max_ and AUC of curcumin metabolites. These findings highlight that optimization of absorption is not always directly reflected in the plasma levels of the parent compound alone, but also in subsequent metabolic dynamics. This observation is particularly important for the interpretation of pharmacokinetic studies based exclusively on intact curcumin.

The kinetics of curcumin degradation when pH changes occur over a wider range (from 3 to 10) has been the subject of research for a long time [220], revealing the increased concern of researchers regarding this challenge. Thus, curcumin has also been investigated in pH-sensitive injectable systems, in the form of inorganic X-LDH/CRC nanoparticles obtained by loading onto exfoliated LDH [150]. Although classical pharmacokinetic parameters were not reported, in vivo evaluations using an A549 xenograft model demonstrated a significant reduction in tumor mass (2.03 ± 0.58 g versus ~ 3.8 g for free curcumin), in the absence of systemic toxicity. The pH-dependent release profile (~ 96% at pH 5 versus ~ 41% at pH 7.4) and good hemocompatibility (< 5% hemolysis) suggest increased local availability of curcumin. However, the lack of a complete pharmacokinetic study limits extrapolation to systemic behavior.

A distinct example is the multilayer S/O/W emulsion system [173], which does not aim to increase systemic exposure, but rather to analyze gastrointestinal distribution. The preferential distribution of curcumin in the small intestine, together with the colonic retention of fucoxanthin (the co-encapsulated compound), demonstrates that bioavailability can also be conceptualized as targeted local availability, rather than exclusively as total plasma exposure. This type of approach is particularly relevant for nutritional applications or intestinal pathologies, but cannot be directly compared with classical pharmacokinetic studies.

Complementarily, studies that quantify serum levels following oral administration at fixed time points or over limited intervals provide useful indications of in vivo absorption. However, such data remain insufficient for complete pharmacokinetic characterization, as they do not allow reliable estimation of AUC or clearance. In this context, Rivera-Pérez et al. [68] evaluated absorption following intragastric administration and observed a maximum serum signal at approximately 2 h, while both the nanoemulsion and the microencapsulated formulation generated significantly higher serum levels than curcumin administered in water.

Comparative analysis of these studies indicates that formulations for which key pharmacokinetic parameters are reported provide the most robust evidence for improved systemic bioavailability of curcumin. Nevertheless, the relatively limited number of in vivo studies that include rigorous pharmacokinetic characterization highlights an important limitation of the current literature and offers opportunities for further investigation. Solid dispersions obtained by HME and polymer-based micro- or nanoencapsulated systems stand out for their favorable balance between enhanced absorption and controlled release. In contrast, studies relying on pharmacodynamic indicators or local tissue distribution should be interpreted as indirect evidence, relevant in specific contexts but limited in terms of direct comparison. A critical direction for future research is the expansion of in vivo studies with harmonized pharmacokinetic methodologies, enabling rigorous and comparable evaluation of different formulation strategies and avoiding overestimation of bioavailability based on incomplete indicators.

## Conclusions and Future Perspectives

Curcumin is a remarkable natural compound with broad therapeutic potential, supported by compelling evidence of its antioxidant, anti-inflammatory, anticancer, and neuroprotective activities. Despite this promise, its practical implementation in food, pharmaceutical, and biomedical applications is severely hampered by intrinsic physicochemical and pharmacokinetic limitations, including poor aqueous solubility, chemical instability under physiological conditions, and extremely low oral bioavailability. These limitations explain why conventional formulations often fail and provides strong evidence that microencapsulation is not simply a formulation improvement, but an essential strategy to support the effective use of curcumin.

This review highlights that microencapsulation plays an important role in transforming curcumin from an unstable bioactive compound into a viable functional component. Encapsulation strategies provide physicochemical protection, enhance apparent solubility, and enable controlled and targeted release, thereby improving the bioaccessibility and functional performance. A wide range of techniques, including conventional methods such as spray drying, ionotropic gelation, and complex coacervation, as well as advanced methods including electrospraying, nano-spray drying, extrusion-based technologies, and inorganic or hybrid delivery systems, have been critically examined. The collective evidence indicates that the formulation performance is strongly controlled by the carrier composition, encapsulation architecture, and processing conditions, with protein-based and amphiphilic polymeric systems often providing increased stabilization through specific molecular interactions with curcumin.

At the same time, the comparative analysis provides clear evidence that no single encapsulation technique can be considered universally optimal. Each method presents inherent trade-offs related to thermal stress, sensitivity to process parameters, scalability, long-term stability, and release control. As a result, the rational selection of encapsulation strategies should follow a fit-for-purpose paradigm, in which technological feasibility, cost-effectiveness, and industrial scalability are balanced against the functional requirements of the intended application, whether nutritional, pharmaceutical, cosmetic, or biomedical.

A central and distinctive contribution of this review is the integration of experimental microencapsulation strategies with molecular docking and molecular dynamics simulations. By elucidating curcumin–carrier interactions at the molecular level, in silico approaches provide predictive insights into binding affinity, conformational stability, and environmental response. When combined with experimental formulation data, these tools support the shift from empirical trial-and-error development toward rational, mechanism-driven formulation design, supporting informed carrier selection and targeted optimization of stability and release behavior.

Despite the significant progress presented in this review, several limitations should be acknowledged. Direct and systematic comparisons of in vivo pharmacokinetic parameters across different curcumin delivery systems remain scarce, which limits the quantitative assessment of bioavailability enhancement beyond qualitative trends or in vitro observations. In addition, the scalability, long-term stability, and safety of certain carrier materials, particularly complex hybrid or inorganic systems, require further validation before translation beyond laboratory-scale studies can be considered. From a computational perspective, molecular docking and molecular dynamics simulations provide valuable mechanistic insight into curcumin–carrier interactions, but remain constrained by force-field accuracy, sampling limitations, and the need for consistent experimental validation, especially in heterogeneous and multicomponent encapsulation environments.

Future research should focus on the development of hybrid and multifunctional delivery systems that combine the scalability of established encapsulation techniques with the precision and adaptability of advanced materials. Greater attention should be paid to application-specific design, particularly systems tailored for gastrointestinal targeting, site-specific release, and responsive behavior triggered by pH, enzymes, or pathological microenvironments. In parallel, advances in multiscale computational modeling, improved force fields, and long-run simulations are expected to further increase the predictive power of in silico tools, enabling more accurate modeling of complex, multicomponent delivery systems under realistic biological conditions. Furthermore, the integration of encapsulation design with translational considerations, including regulatory compliance, sustainability of materials, industrial feasibility, and long-term stability during storage, should also be prioritized in future studies. The combination of green processing technologies, biocompatible carriers, and predictive computational frameworks represents a particularly promising direction to accelerate the translation of curcumin-based formulations from laboratory research to commercial products.

In conclusion, the convergence of microencapsulation technologies with molecular-level computational modeling provides a robust and integrative framework for the rational development of stable, bioavailable, and application-specific curcumin delivery systems. This integrated approach would be expected to play a key role in advancing curcumin from a scientifically compelling molecule to a consistently effective ingredient in next-generation food, pharmaceutical, and biomedical products.

## Data Availability

No datasets were generated or analyzed during the current study.
